# Global Burden of cardiomyopathy and myocarditis in the older adults from 1990 to 2019

**DOI:** 10.3389/fpubh.2022.1018385

**Published:** 2022-09-23

**Authors:** Kexin Zhang, Xuebing Cheng, Na Qu, Hongwei Song, Youhong Luo, Tongtong Ye, Qian Xu, Hongzhan Tian, Chengxia Kan, Ningning Hou

**Affiliations:** ^1^Department of Endocrinology and Metabolism, Affiliated Hospital of Weifang Medical University, Weifang, China; ^2^Clinical Research Center, Affiliated Hospital of Weifang Medical University, Weifang, China; ^3^Department of Pathology, Affiliated Hospital of Weifang Medical University, Weifang, China

**Keywords:** aging, chronic disease, cardiomyopathy, myocarditis, older adults, incidence

## Abstract

**Background:**

Cardiomyopathy and myocarditis (CM-MC) are common chronic diseases causing heart failure in older adults. We aimed to analyze the burden of CM-MC in older adults aged 60–89 years at the global, regional, and national levels in 204 countries from 1990 to 2019.

**Methods:**

Detailed data on CM-MC from 1990 to 2019 were analyzed from the Global Burden of Diseases Study 2019, including incidence, mortality, disability-adjusted life years (DALYs) and the proportion of deaths caused by different risks factors. All results are presented as numbers, age-standardized rates per 100,000 person-years and estimated annual percentage change (EAPC) with an uncertainty interval of 95%.

**Results:**

Globally, there were 475,458 (339,942–638,363) incidence cases from CM-MC in 2019; with an age-standardized incidence rate (ASIR) of 16 (13–19.3) per 100,000 person-years. And there were 185,308 (154,610–200,448) deaths, with the age-standardized mortality rate (ASMR) being 4.4 (3.7–4.8). CM-MC resulted in 3,372,716 (2,931,247–3,693,622) DALYs, with an age-standardized DALYs rate (ASDR) of 114.8 (98.7–126.1). Estimated annual percentage change (EAPCs) for ARIS, ARMS, and ARDS has decreased. At the national level, the United States of America had the highest mortality [21,372 (18,924–24,241)] and disability-adjusted life years [407,712 (370,234–470,165)]. And China had the highest number of incident cases [122, 266 (85,925–166,095)]. Globally, high systolic blood pressure and alcohol consumption were the top two risk factors for the proportion of CM-MC deaths.

**Conclusion:**

CM-MC is still an important cause of early death and chronic disability in older adults. Based on this study, public health agencies should seek more effective methods to prevent and treat CM-MC.

## Introduction

Cardiovascular diseases are primary chronic diseases and global health problems characterized by high morbidity, disability and mortality (1, 2). Among cardiovascular diseases, cardiomyopathy and myocarditis (CM-MC) commonly cause heart failure, imposing a substantial disease burden, especially on older adults (1, 3). Cardiomyopathy can cause the heart muscle to weaken and reduce the heart's ability to pump blood, which in severe cases can lead to progressive heart failure or cardiovascular death (4). Myocarditis is caused by inflammation or viral infection. And prolonged inflammatory changes can cause irreversible damage to the myocardium, leading to cardiomyopathy (5–10). The world's population is aging rapidly, and multimorbidity, especially cardiovascular disease, is prevalent in older adults (11). This rapid increase in age in the world population requires special attention for older adults; cardiovascular risk factors play a vicious role in the development of CM-MC in older adults (12). Notably, the onset of CM-MC is relatively different in older patients compared to young and middle-aged patients since older adults usually have weaker organs, poorer perception, and less pronounced symptoms.

The Global Burden of Disease (GBD) study reports more than 350 diseases and injuries burden in 204 countries and territories from 1990 to 2019. The updated GBD 2019 provides the most recent data available for CM-MC. The study published in GBD 2017 showed that there were 1.8 million cases of myocarditis, 1.62 million cases of alcoholic cardiomyopathy and 4.21 million cases of other cardiomyopathies worldwide in 2017 (13). Globally, 4,648,688,890 and 233,159 people died from myocarditis, alcoholic cardiomyopathy and other cardiomyopathies, respectively. Between 1990 and 2017, the age-standardized rates of myocarditis, alcoholic cardiomyopathy and other cardiomyopathies decreased by 6.9, 26.2, and 15.5% (13).

This study clearly showed the CM-MC burden for 2017. However, this study did not address risk factors for CM-MC; and few studies have assessed the changing patterns of CM-MC among older people globally. Additionally, data in the GBD are constantly being updated due to the aging of the population. Thus, the current study aimed to provide incidence, deaths, and disability-adjusted life years (DALYs) for CM-MC in older adults aged 60–89 years in 204 countries and territories from 1990 to 2019.

## Methods

### Data sources

Data on the burden of disease due to CM-MC were drawn from the Global Health Data Exchange query tool (http://ghdx.healthdata.org/gbd-results-tool) created by the GBD collaborators. Detailed methodologies of GBD 2019 have been described before (14, 15). The disease burden for CM-MC in older adults (60–89 years) for 204 countries and territories was assessed systematically by deaths, incidence and DALYs according to sex and region were classified into 5 regions based on sociodemographic index (SDI) quintiles between 1990 and 2019. The SDI reflected the social and economic conditions of a region with five quintiles (low SDI, low-middle SDI, middle SDI, high-middle SDI and high SDI quintiles).

Additionally, the world was also divided into 21 geographical areas. The relationship of EAPCs between ASRs and human development index (HDI) was also analyzed to determine the influencing factors of EAPCs from Human Development Reports (https://hdr.undp.org//en/composite/HDI).

### Statistical analysis

The burden of CM-MC is presented as numbers, age-standardized incidence rate (ASIR), age-standardized mortality rate (ASMR), age-standardized DALYs rate (ASDR), and estimated annual percentage change (EAPC) with 95% uncertainty intervals. ASRs are calculated by standardizing the global age structure, which is vital when populations are compared in different locations over different periods (16). The EAPC was calculated as 100 × (exp(β)-1), and the 95% CI was also acquired from the linear regression model. An increasing ASR trend was defined as an EAPC estimation and minimum lower limit of the 95% CI being both >0. Conversely, decreased ASR was defined as an EAPC estimation, with the maximum upper limit of the 95% CI being < 0. Statistical analysis was performed using R-Studio version 4.1.2, and *P* < 0.05 was considered statistically significant. The detailed analysis methods used in the study have been previously reported (17–19).

## Results

### Global Burden of CM-MC

**Figure 1:**
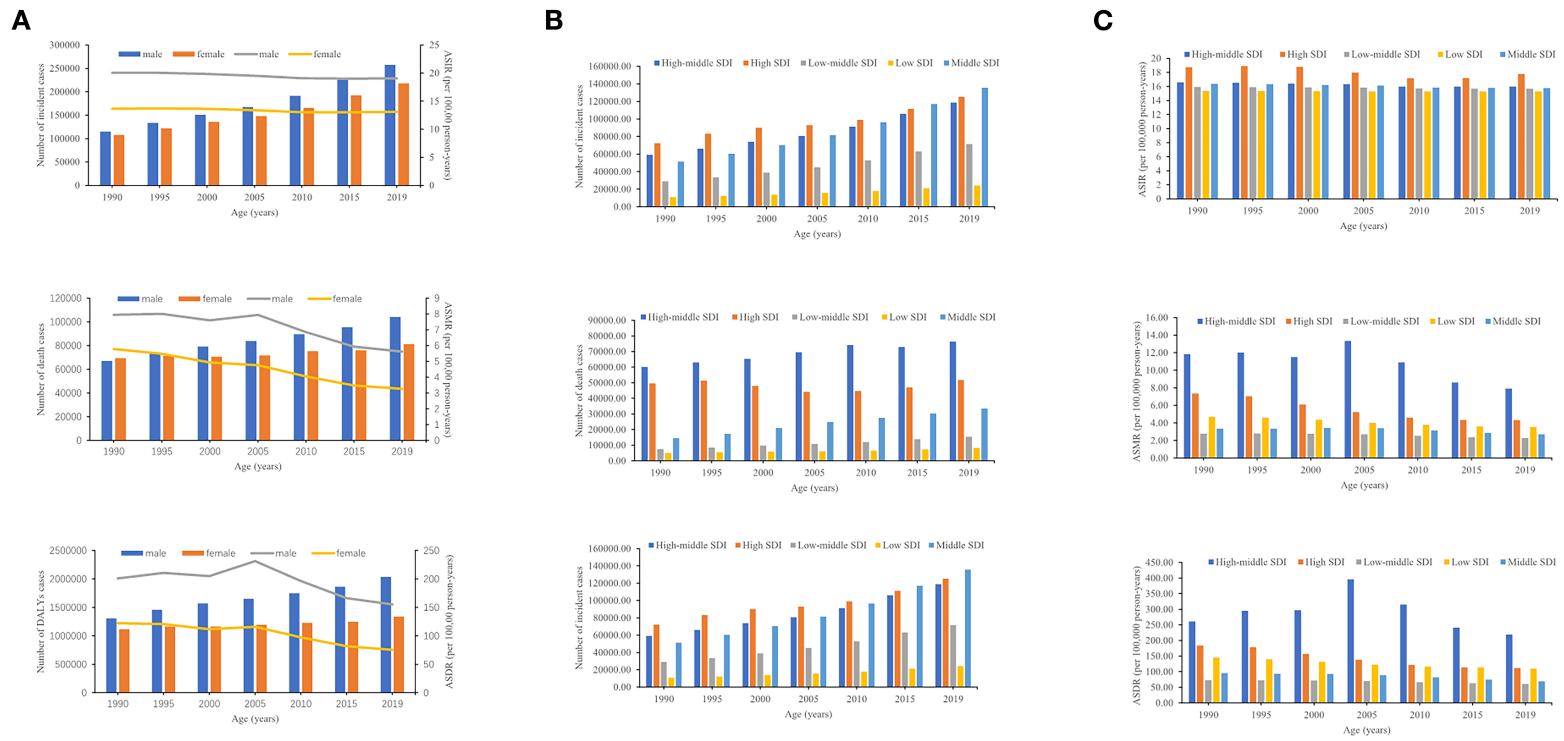
The number of cases and ASRs of CM-MC from 1990 to 2019. **(A)** The number of cases of CM-MC caused by global, from 1990 to 2019, **(B)** The number of cases of CM-MC caused by SDI regions, from 1990 to 2019, **(C)** The ASRs of CM-MC caused by SDI regions, from 1990 to 2019. SDI, sociodemographic index.

Globally, a total of 475,458 (339,943–638,363) cases of CM-MC occurred in 2019. The incidence rate increased by 1.1% from 222,997 (158,611–302,867) in 1990. However, the ASIR at the global level has decreased by 0.23% from 16.7 (13.5–20.3) in 1990 to 16 (13–19.3) in 2019. Similarly, the mortality in both sexes increased by 0.4% from 136,568 (108,539–146,288) in 1990 to 185,308 (154,610–200,448) in 2019. In 1990, the global ASMR for CM-MC was 6.9 (5.7–7.4) per 100,000 population, which decreased to 4.4 (3.7–4.8) in 2019. CM-MC caused 3,372,716 (2,931,247–3,693,622) DALYs in 2019, which was a 0.4% increase from 2,424,586 (2,073,563–2,600,458) in 1990. The ASDR showed a decrease of 1.2%, from 161.2 (146.7–185.6) in 1990 to 114.8 (98.7–126.1) in 2019 per 100,000 population (Table 1; Figure 1A).

**Table 1:** Incident cases, death, and DALYs of cardiomyopathy and myocarditis and ASRs per 100,00 population from 1990 and 2019 by Global Burden of Disease.

**Characteristics**	**1990**		**2019**		**1990–2019**	**1990–2019**
	**Incidence cases** **No. (95% UI)**	**ASIR per 100,000** **No. (95% UI)**	**Incidence cases** **No. (95% UI)**	**ASIR per 100,000** **No. (95% UI)**	**Percentage change** **No. (95% CI)**	**EAPC** **No. (95% CI)**
**Overall**	222,997.3 (158,611.1–302,866.6)	16.7 (13.5–20.3)	475,458.1 (339,942.5–638,363.1)	16 (13–19.3)	1.1% (1.1–1.2)	−0.23 (−0.26 to −0.21)
**Socio-demographic index**						
Low	10,902.6 (7,773.9–14,821.4)	15.3 (12.4–18.7)	24,200.3 (17,313.1–32,588.4)	15.3 (12.4–18.6)	1.1% (1.1–1.2)	−0.02 (−0.02 to −0.01)
Low-middle	28,993.4 (20,739.3–39,287.8)	15.9 (12.9–19.3)	71,636 (51,137.1–97,244.5)	15.7 (12.7–19)	1.5% (1.4–1.5)	−0.06 (−0.07 to −0.06)
Middle	51,448.8 (36,306.6–70,136.8)	16.4 (13.3–20)	135,590.8 (95,814.5–183,590.5)	15.8 (12.8–19.1)	1.4% (1.4–1.5)	−0.16 (−0.17 to −0.14)
High-middle	59,286 (42,129.2–80,971.8)	16.6 (13.3–20.2)	118,754 (84,520.7–160,137)	16 (12.9–19.3)	1% (0.9–1.1)	−0.16 (−0.18 to −0.14)
High	72,258.9 (50,196.8–98,339.6)	18.7 (15.1–22.8)	125,059.1 (89,750.9–165,820.2)	17.8 (14.7–21.2)	0.7% (0.7–0.8)	−0.41 (−0.49 to −0.34)
**Regions**						
Andean Latin America	1,017.6 (721.7–1,362.7)	14.1 (11.4–17.1)	2,969.2 (2,092.2–4,011)	14.1 (11.4–17.1)	1.9% (1.9–2)	0.01 (0–0.02)
Australasia	1,431 (1,000.7–1,945.7)	16.5 (13.3–20)	3,019 (2,101.2–4,078.5)	16.4 (13.3–19.8)	1.1% (1.1–1.2)	−0.02 (−0.04 to −0.01)
Caribbean	1,413.1 (985.4–1,911.1)	14.2 (11.5–17.2)	2,795.5 (1,946.8–3,782)	14.2 (11.5–17.2)	1% (1–1)	0 (0–0)
Central Asia	2,108.5 (1,496.4–2,845.1)	14.8 (11.9–17.9)	3,003 (2,122.7–4,048.4)	14.9 (11.9–18)	0.4% (0.4–0.5)	0.01 (0.01–0.01)
Central Europe	7,646.7 (5,391.1–10,322.4)	16.3 (13.1–19.8)	12,170.7 (8,484.1–16,402.3)	16.5 (13.3–20)	0.6% (0.6–0.6)	0 (−0.04–0.03)
Central Latin America	4,330.7 (3,080.5–5,864.7)	15.1 (12.2–18.3)	12,955.8 (9,137.6–17,599.3)	15 (12.1–18.2)	2% (2–2)	−0.02 (−0.02 to −0.02)
Central Sub-Saharan Africa	953.6 (668.8–1,304.8)	14.6 (11.7–17.8)	2,134.9 (1,520.9–2,887.5)	14.5 (11.7–17.7)	1.2% (1.2–1.3)	−0.02 (−0.02 to −0.01)
East Asia	47,442.6 (33,102.5–64,904.7)	17.9 (14.5–22)	125,940.4 (88,474.2–171,088.4)	16.9 (13.7–20.4)	1.7% (1.5–1.8)	−0.28 (−0.32 to −0.23)
Eastern Europe	16,257.5 (11,643.9–22,093)	17.6 (14.2–21.4)	21,318.4 (15,416.6–28,978.1)	17.7 (14.3–21.5)	0.3% (0.3–0.4)	0.02 (0.01–0.02)
Eastern Sub-Saharan Africa	3,460.7 (2,471.3–4,701.6)	15.3 (12.3–18.6)	7,312.2 (5,233.6–9,888.2)	15.2 (12.3–18.6)	1.1% (1.1–1.2)	−0.01 (−0.01 to −0.01)
High-income Asia Pacific	12,953 (9,066.6–17,640.3)	20.7 (16.8–25)	29,830.8 (20,687.7–40,299.4)	20.1 (16.4–24.1)	1.3% (1.1–1.5)	−0.2 (−0.24 to −0.17)
High-income North America	28,478.8 (19,637.5–38,964.4)	19.9 (16–24.3)	44,062.4 (32,938.2–56,722.2)	18.2 (15.4–21.4)	0.5% (0.4–0.7)	−0.83 (−1 to −0.66)
North Africa and Middle East	6,233.9 (4,370.8–8,427.7)	12 (9.7–14.6)	15,867.8 (11,168.7–21,292.8)	12 (9.7–14.7)	1.5% (1.5–1.6)	0.02 (0.02–0.02)
Oceania	123.2 (85.6–169.5)	15.3 (12.3–18.5)	281.4 (197.1–387.6)	15.3 (12.3–18.5)	1.3% (1.3–1.3)	0 (0–0)
South Asia	27,011.3 (19,273.8–36,803.6)	16.3 (13.2–19.7)	75,466.7 (53,955.2–102,649.1)	16.2 (13.1–19.6)	1.8% (1.7–1.9)	−0.02 (−0.02 to −0.02)
Southeast Asia	11,204 (7,999.5–15,009)	15.1 (12.3–18.4)	27,687.3 (19,909.4–37,031.8)	15.1 (12.3–18.4)	1.5% (1.4–1.5)	0 (−0.01–0)
Southern Latin America	2,777.8 (1,970–3,732.3)	15.3 (12.4–18.6)	5,172.3 (3,653.6–6,938.8)	15.4 (12.4–18.7)	0.9% (0.8–0.9)	0.01 (0–0.02)
Southern Sub-Saharan Africa	1,403.1 (1,009.1–1,889)	15.7 (12.7–19.1)	2,868.8 (2,074.4–3,873.6)	15.7 (12.7–19.1)	1% (1–1.1)	0 (0–0.01)
Tropical Latin America	4,917.1 (3,500.1–6,745.1)	15.8 (12.7–19.2)	14,030.5 (9,950–19,052.9)	15.7 (12.7–19.1)	1.9% (1.8–1.9)	−0.01 (−0.01 to −0.01)
Western Europe	37,558.4 (26,207.6–51,043.3)	17.6 (14.2–21.3)	58,046.7 (40,184.5–78,240.3)	17.5 (14.2–21.1)	0.5% (0.5–0.6)	−0.06 (−0.09 to −0.03)
Western Sub-Saharan Africa	4,274.7 (3,060.5–5,790.1)	15.5 (12.5–18.9)	8,524.5 (6,114.6–11,482.4)	15.4 (12.5–18.7)	1% (1–1)	−0.03 (−0.03 to −0.02)
**Characteristics**	**1990**		**2019**		**1990-2019**	**1990-2019**
	**Deaths cases** **No. (95% UI)**	**ASMR per 100,000** **No. (95% UI)**	**Deaths cases** **No. (95% UI)**	**ASMR per 100,000** **No. (95% UI)**	**Percentage change** **No. (95% CI)**	**EAPC** **No. (95% CI)**
**Overall**	136,567.7 (108,539.4–146,287.7)	6.9 (5.7–7.4)	185,307.7 (154,609.6–200,448.3)	4.4 (3.7–4.8)	0.4% (0.3–0.5)	−1.63 (−1.82 to −1.44)
**Socio-demographic index**						
Low	4,952.6 (3,192.9–6,556.3)	4.7 (3–6.2)	8,188 (6,156.5–10,354.3)	3.5 (2.7–4.4)	0.7% (0.3–1.2)	−1.11 (−1.16 to −1.06)
Low-middle	7,381.2 (6,039.3–8,725.1)	2.8 (2.2–3.3)	15,429.3 (12,333.5–17,487.3)	2.3 (1.9–2.6)	1.1% (0.8–1.4)	−0.73 (−0.85 to −0.62)
Middle	14,496.9 (12,622.8–18,103.4)	3.4 (2.9–4.3)	33,420.7 (26,353.4–37,625)	2.7 (2.1–3.1)	1.3% (0.9–1.7)	−0.74 (−0.91 to −0.57)
High-middle	60,107.6 (44,975.6–64,627.4)	11.9 (9.1–13)	76,394.9 (58,737.7–84,110.1)	7.9 (6–8.8)	0.3% (0.2–0.4)	−1.36 (−1.79 to −0.93)
High	49,557.9 (37,568.6–53,159.8)	7.3 (5.7–7.8)	51,771.1 (45,566.4–59,744.2)	4.3 (3.8–4.9)	0% (−0.1–0.3)	−2.22 (−2.37 to −2.06)
**Regions**						
Andean Latin America	209.4 (154.9–257)	2.2 (1.6–2.6)	466 (357.8–617.9)	1.6 (1.2–2)	1.2% (0.7–1.9)	−0.91 (−1.09 to −0.73)
Australasia	994.7 (692.4–1,094.2)	7.4 (5.2–8.1)	1,077.9 (927.5–1,335.7)	3.6 (3.2–4.4)	0.1% (−0.1–0.6)	−2.56 (−2.87 to −2.25)
Caribbean	685.7 (581.7–873.5)	5.1 (4.2–6.6)	1,472.4 (1,239.3–1,739.1)	5.4 (4.4–6.6)	1.1% (0.7–1.6)	0.43 (0.26–0.6)
Central Asia	999.3 (857.6–1,291.3)	5.2 (4.5–7.2)	3,174.8 (1,887.6–3,791.4)	11 (6.5–13)	2.2% (0.7–3)	3.95 (2.85–5.06)
Central Europe	16,649.1 (12,044.8–18,475.5)	17.8 (13.4–19.6)	21,039.9 (16,046.5–24,082.2)	14.1 (10.4–16.2)	0.3% (0.1–0.4)	−0.97 (−1.15 to −0.79)
Central Latin America	1,043.7 (899.8–1,130.2)	2.4 (2.1–2.6)	2,542 (2,102–3,037.6)	2 (1.6–2.4)	1.4% (1–1.9)	−1.12 (−1.32 to −0.93)
Central Sub-Saharan Africa	1,085.9 (603.8–1,626.6)	10.1 (5.6–14.9)	2,190 (1,266–3,579.9)	8.9 (5.3–14.2)	1% (0.5–1.8)	−0.48 (−0.5 to −0.45)
East Asia	6,151.5 (4,801.4–10,248.4)	2 (1.6–3.3)	17,227.1 (11,390.2–20,648)	1.8 (1.2–2.1)	1.8% (0.5–3)	0.02 (−0.27–0.31)
Eastern Europe	13,208.1 (11,381.5–20,093.2)	14.1 (11.9–20.7)	26,240.5 (17,561.6–30,642.4)	24.1 (17.6–28)	1% (0–1.5)	1.96 (0.92–3)
Eastern Sub-Saharan Africa	1,437.9 (1,029.2–1,788.4)	4.8 (3.3–6)	2,657.2 (1,881.6–3,340.8)	3.9 (2.8–5)	0.8% (0.4–1.4)	−0.72 (−0.79 to −0.64)
High-income Asia Pacific	5,455.3 (2,827.6–6,101.2)	5 (2.9–5.5)	6,423.2 (4,230.8–7,375.7)	2.2 (1.6–2.4)	0.2% (0–0.7)	−2.74 (−3.09 to −2.39)
High-income North America	18,161.4 (14,023.2–19,403.7)	8 (6.4–8.5)	22,421.9 (19,907.4–25,657.8)	5.9 (5.3–6.6)	0.2% (0.1–0.5)	−1.71 (−1.92 to −1.49)
North Africa and Middle East	2,839.1 (2,173.6–4,211.6)	3.8 (2.8–5.5)	5,281.8 (4,343–7,304.9)	2.6 (2.1–3.6)	0.9% (0.4–1.3)	−1.33 (−1.38 to −1.29)
Oceania	43.6 (29.6–62.6)	3.6 (2.4–5.1)	98.7 (66.5–131.6)	3.7 (2.4–5.1)	1.3% (0.8–1.9)	0.23 (0.17–0.29)
South Asia	1,865.4 (1,307.5–2,684)	0.7 (0.5–1)	4,495.7 (3,453.1–5,867.6)	0.6 (0.5–0.8)	1.4% (0.9–2.1)	−0.7 (−0.76 to −0.64)
Southeast Asia	4,964.6 (3,659.7–6,031.4)	4.2 (3.1–5.1)	11,707.9 (8,571.4–13,953.1)	3.9 (2.8–4.6)	1.4% (1–1.8)	−0.25 (−0.37 to −0.12)
Southern Latin America	3,796.3 (2,719.3–4,447)	12.8 (9.5–14.9)	5,051.5 (4,233.9–5,703.6)	8.8 (7.4–9.8)	0.3% (0.1–0.7)	−1.69 (−1.88 to −1.51)
Southern Sub-Saharan Africa	1,956.6. (1.493.2–2,376.3)	12.8 (9.9–15.3)	3,653.4 (3,039.3–4,619.4)	11.5 (9.5–14.5)	0.9% (0.6–1.2)	−0.46 (−0.74 to −0.17)
Tropical Latin America	7,754.5 (5,831.2–8,346.3)	15.6 (12.2–16.7)	13,308.9 (11,694.1–16,038.2)	9.3 (8.2–10.9)	0.7% (0.5–1.3)	−2.38 (−2.61 to −2.14)
Western Europe	43,305.3 (25,337.9–47,870.7)	10.6 (6.5–11.8)	29,438.8 (25,716.5–35,184.7)	4.5 (3.9–5.3)	−0.3% (−0.4–0.2)	−3.38 (−3.6 to −3.15)
Western Sub-Saharan Africa	3,960.1 (2,309.6–5,288.8)	9 (5.2–12.1)	5,338.3 (3,862.7–6,515.9)	6 (4.4–7.3)	0.3% (0–1)	−1.68 (−1.82 to −1.54)
**Characteristics**	**1990**		**2019**		**1990-2019**	**1990-2019**
	**DALYs cases** **No. (95% UI)**	**ASDR per 100,000** **No. (95% UI)**	**DALYs cases** **No. (95% UI)**	**ASDR per 100,000** **No. (95% UI)**	**Percentage change** **No. (95% CI)**	**EAPC** **No. (95% CI)**
**Overall**	2,424,586.4 (2,073,563.4–2,600,458.2)	161.2 (146.7–185.6)	3,372,716 (2,931,247.4–3,693,622.4)	114.8 (98.7–126.1)	0.4% (0.3–0.5)	−1.2 (−1.52 to −0.89)
**Socio-demographic index**						
Low	114,851.5 (78,312.7–147,326.4)	144.8 (94–187.9)	191,756.5 (150,091.6–235,172.6)	110 (86.2–134.9)	0.7% (0.4–1.1)	−1.02 (−1.07 to −0.97)
Low-middle	146,748.7 (121,193.9–172,264.4)	73 (57.6–93.3)	296,947.9 (245,156.9–339,725.6)	60.9 (50.2–70.1)	1% (0.8–1.3)	−0.67 (−0.75 to −0.58)
Middle	280,050.9 (246,573.5–352,490.4)	95.4 (80.8–133.6)	603,684.6 (502,239.7–704,347.8)	69.2 (59.6–83.2)	1.2% (0.8–1.4)	−1.12 (−1.26 to −0.99)
High-middle	1,012,265.2 (876,809.7–1,104,530.8)	260.7 (241.8–315.8)	1,365,628.8 (1,094,641.4–1,504,938.3)	219.2 (170.1–245.2)	0.3% (0.2–0.5)	−0.54 (−1.19–0.12)
High	869,432.8 (680,240.2–941,069)	184.1 (151.1–195.3)	912,799.3 (826,293–1,062,011.4)	111.8 (102.5–125.5)	0% (0–0.3)	−2.04 (−2.16 to −1.93)
**Regions**						
Andean Latin America	3,768 (2,815.4–4,573.2)	66.6 (45.9–84.2)	8,383 (6,490.2–10,954.9)	44.1 (34.1–56.3)	1.2% (0.7–1.9)	−1.33 (−1.54 to −1.12)
Australasia	19,434.1 (14,488.6–21,677.4)	195.8 (153.8–210)	20,971 (18,383–25,772.2)	99.5 (90.1–122.1)	0.1% (−0.1–0.5)	−2.42 (−2.63 to −2.2)
Caribbean	13,230.7 (11,210.9–16,712.6)	165.8 (128.2–231)	27,852.3 (23,409.6–33,107.4)	171.8 (130.6–228.6)	1.1% (0.7–1.5)	0.35 (0.2–0.51)
Central Asia	20,105.8 (17,330.7–27,067.2)	166.2 (142.4–241.8)	64,872.2 (39,263.1–77,584.4)	330.3 (204.4–393)	2.2% (0.7–3.1)	3.59 (2.51–4.68)
Central Europe	263,358.6 (201,499.7–288,636.8)	341.4 (284.2–366.1)	331,892.7 (256,841.4–379,220.1)	276.6 (204.7–320.9)	0.3% (0.1–0.4)	−0.79 (−0.87 to −0.71)
Central Latin America	19,529.5 (16,938.2–21,069.8)	64.6 (57.3–70.2)	46,103.5 (38,668.4–54,768.1)	54.6 (44–65.7)	1.4% (1–1.8)	−0.82 (−0.94 to −0.7)
Central Sub-Saharan Africa	24,855.7 (14,650.1–36,029.5)	288.4 (158–397.7)	48,586.3 (30,769.2–76,055)	242.2 (152.5–358.4)	1% (0.5–1.7)	−0.61 (−0.63 to −0.59)
East Asia	122,714.1 (98,083.5–194,682.5)	73.3 (57.7–125.2)	298,378 (212,246.5–354,635.8)	49.8 (36–58.1)	1.4% (0.4–2.3)	−1.13 (−1.36 to −0.89)
Eastern Europe	274,999 (229,461.8–458,560.5)	473.9 (393.8–731)	528,573.6 (399,923.7–618,923)	862.4 (662–998.7)	0.9% (0–1.5)	2.08 (0.93–3.24)
Eastern Sub-Saharan Africa	38,923.3 (29,214.9–48,106.4)	185.5 (127.4–232.8)	74,519.7 (56,281–93,225.8)	147.1 (105.1–190.2)	0.9% (0.6–1.3)	−0.79 (−0.85 to −0.73)
High-income Asia Pacific	89,404.2 (54,501.5–99,542)	118.5 (97.2–126.9)	106,001.9 (76,891.4–120,523)	58.3 (50.9–66.1)	0.2% (0.1–0.6)	−2.52 (−2.68 to −2.36)
High-income North America	356,296.8 (273,348–389,075.6)	237.5 (188.8–253.9)	429,928.4 (390,587.9–499,096)	166.2 (153.4–186.9)	0.2% (0.1–0.6)	−1.69 (−1.85 to −1.53)
North Africa and Middle East	55,697.7 (43,383.5–82,358.4)	129.9 (90.6–212.5)	100,897.3 (83,348.8–139,885.1)	79.5 (64.3–111.5)	0.8% (0.4–1.2)	−1.67 (−1.7 to −1.64)
Oceania	928.9 (624–1,332.2)	112.9 (71.2–171.1)	2,035.1 (1,397.6–2,757)	119.2 (71.7–181.8)	1.2% (0.7–1.8)	0.33 (0.25–0.4)
South Asia	37,048.1 (26,243.2–52,880.3)	18.8 (13.1–26.7)	84,433.2 (65,884.2–110,235)	16.2 (12.5–20.9)	1.3% (0.8–1.9)	−0.57 (−0.61 to −0.53)
Southeast Asia	92,939.8 (73,936.8–118,205.7)	100.6 (81–145.1)	214,038.7 (168,230.7–272,034.8)	89.8 (74–118.4)	1.3% (1–1.8)	−0.36 (−0.47 to −0.25)
Southern Latin America	67,185.2 (49,902.5–77,927.1)	290.8 (235.2–327.8)	84,610.9 (73,609.5–95,680.2)	184.5 (165.4–211)	0.3% (0.1–0.6)	−1.96 (−2.11 to −1.8)
Southern Sub-Saharan Africa	39,338.2 (31,114.4–47,070.4)	308.3 (248.3–358.9)	73,083.9 (60,933.2–91,059.6)	262.9 (219.6–323)	0.9% (0.6–1.2)	−0.66 (−0.85 to −0.46)
Tropical Latin America	146,342.1 (111,307.2–157,578.5)	391.4 (314.4–417.4)	242,491.4 (217,224–290,306.9)	234.2 (209.5–267.3)	0.7% (0.5–1.2)	−2.23 (−2.45 to −2.02)
Western Europe	652,494.4 (426,160.7–715,677.3)	193.8 (141.1–209.3)	463,425 (415,183–553,272.6)	91 (82.8–107.6)	−0.3% (−0.4–0.1)	−2.97 (−3.15 to −2.8)
Western Sub-Saharan Africa	85,992.4 (54,044.4–112,272.1)	231.5 (139.6–304.9)	121,637.9 (92,499.4–147,301.9)	161.2 (122.1–198.4)	0.4% (0.1–1)	−1.45 (−1.58 to −1.33)

ASIR, age-standardized incidence rate; ASMR, age-standardized mortality rate; ASDR, age-standardized DALYs rate; EAPC, estimated annual percentage change; CI, confidence interval; UI, uncertainty interval.

### Regional burden of CM-MC

The number of CM-MC cases rose at the regional level in the five SDI regions (Table 1; Figure 1B). From 1990 to 2019, the number of incidences of CM-MC increased most significantly in low-middle SDI. The number of deaths and DALYs in CM-MC increased most significantly in middle SDI (Table 1; Figure 1B). While the most significant decreases were noted in the high SDI, with ASIR (−0.41; 95% CI: −0.49–0.34), ASMR (−2.22; −2.37–2.06) and ASDR (−2.04; −2.16 to −1.93) of the EAPC (Table 1; Figure 1C; Supplementary Figure 1). The 2019 ASMR and ASDR of CM-MC in Eastern Europe were 24.1 (17.6–28) and 862.4 (662–998.7), respectively, ranking first among the 21 GBD regions in 2019 (Table 1; Supplementary Figures 2B,C). In addition, the highest ASIR of CM-MC per 100,000 population was found in High-income Asia Pacific [20.1 (16.4–24.1)] in 2019 (Table 1; Supplementary Figure 2A). Notably, the most significant increases in the ASMR and ASDR were shown in Central Asia, followed by Eastern Europe from 1990 to 2019. Moreover, the most significant increases in the ASIR were shown in Eastern Europe, North Africa, and the Middle East, followed by Andean Latin America, Central Asia and Southern Latin America. Over the past 30 years, Western Europe had the most significant decreases in the ASMR and ASDR; high-income North America had the most significant decreases in the ASIR (Table 1; Supplementary Figure 3).

### National burden of CM-MC

**Figure 2:**
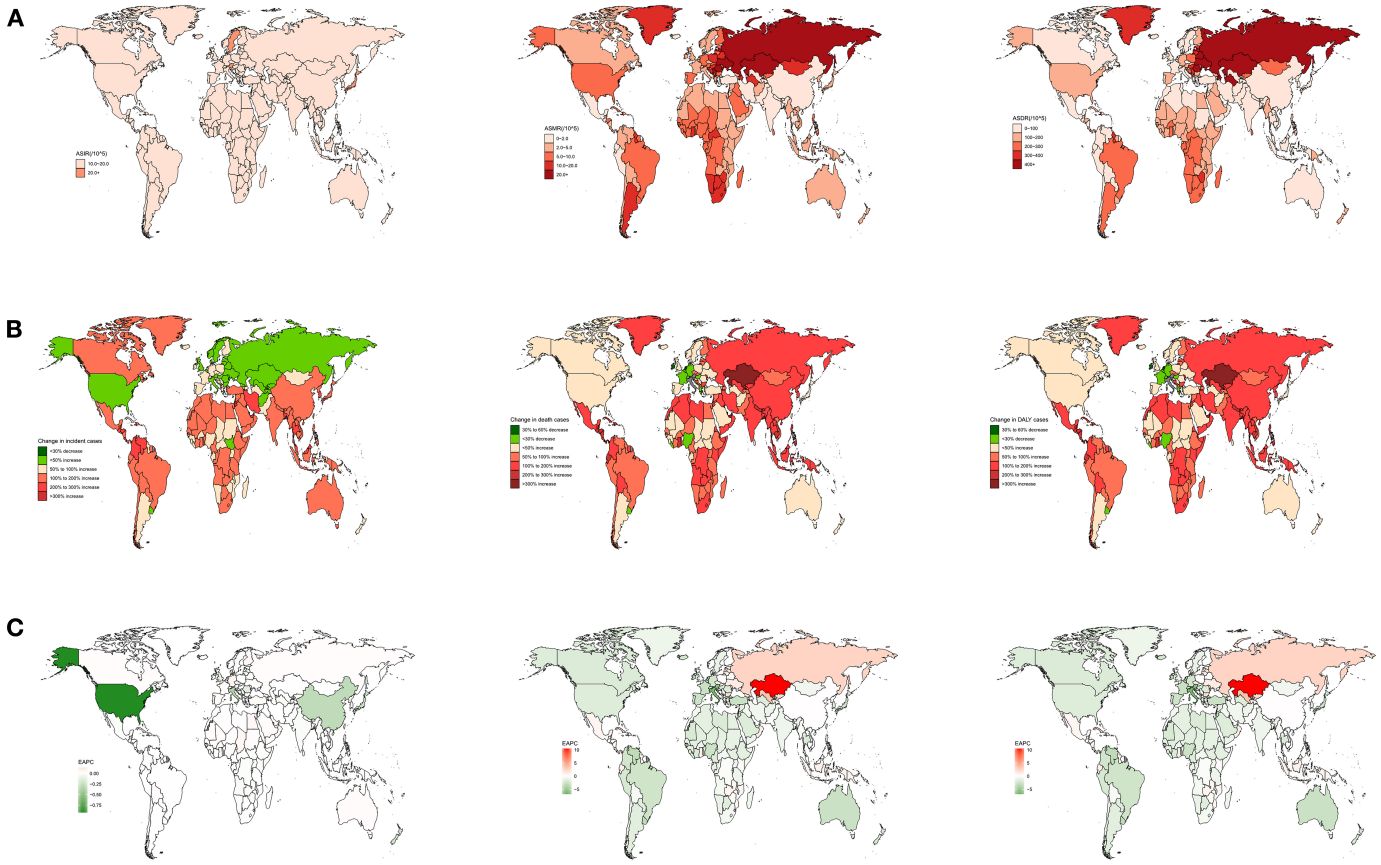
The national burden of CM-MC for both sexes in 204 countries and territories. **(A)** The relative ASRs of CM-MC between 1990 and 2019, **(B)** The relative changes of CM-MC between 1990 and 2019, **(C)** The relative EAPCs of CM-MC s between 1990 and 2019.

China had the highest number of incident cases [122,266 (85,925–166,095)], accounting for almost half of the global totals in 2019 (Supplementary Table 1). The United States of America exhibited the highest numbers of mortalities [21,372 (18,924–24,241)] and DALYs [407,712 (370,234–470,165)]. This accounted for nearly one-eighth of the global totals in 2019 (Supplementary Tables 2, 3). Montenegro owned the highest ASMR [30 (17–41)]; Austria had the highest ASIR [20.6 (17–24.8)]; Ukraine had the highest ASDR [864.5 (674.1–1,164.5)]. In contrast, India showed the lowest ASMR [0.6 (0.4–0.8)] and ASDR [15.3 (11.8–19.8)] due to CM-MC; Tunisia, Turkey, Palestine and Lebanon showed the lowest ASIR due to CM-MC (Supplementary Tables 1–3; Figure 2A). The most significant increase was detected in Qatar from 1990 to 2019, with an 8.4% increase in morbidity; the most significant increase was detected in Kazakhstan from 1990 to 2019, with an 11% increase in deaths and a 9.9% increase in disability-adjusted life years (Supplementary Tables 1–3; Figure 2B). Moreover, the most significant rises in the ASMR and ASDR over the past 30 years were in Kazakhstan; the most significant increases in the ASIR were in Qatar (Supplementary Tables 1–3; Figure 2C). Italy showed the most pronounced decreases in the ASMR and ASDR; the United States of America showed the most pronounced decreases in the ASIR (Supplementary Tables 1–3; Figure 2C). For India, Indonesia, and Pakistan with heavily populated countries, the EAPC of ASMR was [−0.85 (−0.91 to −0.79)], [0.74 (0.68–0.8)], and [−0.27 (−0.4 to −0.15)] from 1990 to 2019, respectively (Supplementary Tables 1–3).

### Influencing factors of EAPC

**Figure 3:**
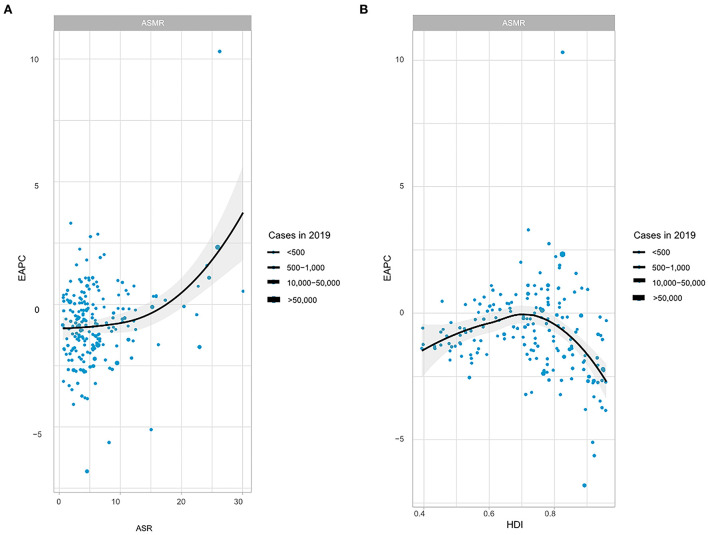
The correlation between EAPC and CM-MC ASMR in 2019 and HDI in 2019. **(A)** The correlation between EAPC and CM-MC ASMR in 2019, **(B)** The correlation between EAPC and HDI in 2019. EAPC, estimated annual percentage change; ASMR, age-standardized mortality rate; HDI, human development index.

A significant association between EAPC and ASR (2019) was observed between EAPC and HDI (2019). The ASMR represents total health life lost from disease occurrence to death, and the HDI can be regarded as an indicator of medical care level. A significant positive association was found between EAPC and ASMR. Surprisingly, a significant negative relation was detected between EAPC and HDI when the HDI was limited to below 0.7 (Figure 3).

### Association of ASRs with SDI

**Figure 4:**
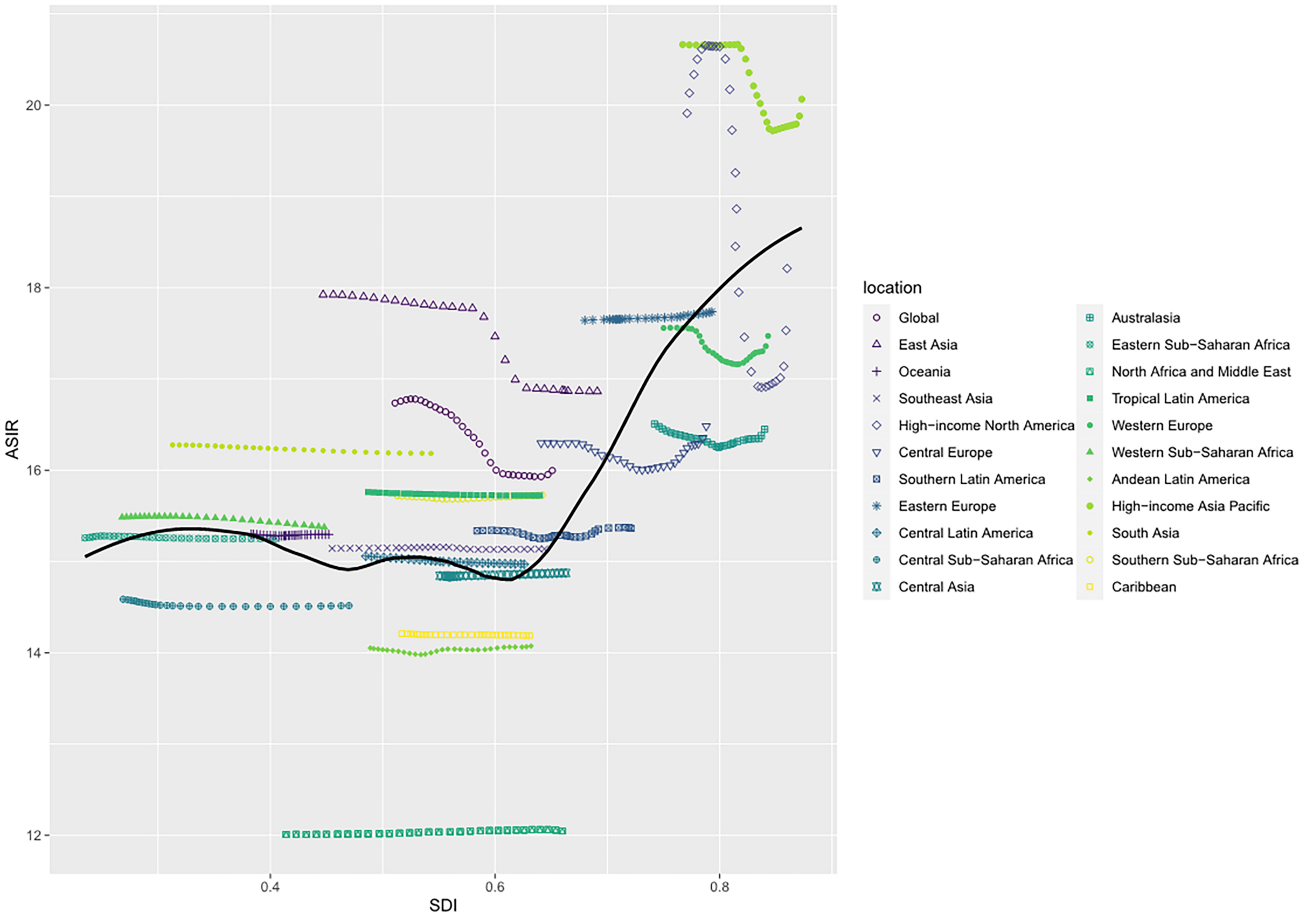
ASIR of cardiomyopathy and myocarditis in global and 21 regions by SDI, 1990–2019. ASIR, age-standardized incidence rate; SDI, sociodemographic index.

We observed a non-linear relationship between ASIR and SDI values. In the High-income Asia-Pacific region, ASIR was highest when the SDI value was 0.82. The lowest ASIR occurred in North Africa and Middle East, with an SDI value of 0.43. At the regional level, eight regions based on SDI, had higher ASIRs than that at the global level in 2019, including High-income Asia Pacific, High-income North America, Eastern Europe, Western Europe, East Asia, Central Europe, Australasia and South Asia (Figure 4). The highest ASMR and ASDR occurred in Eastern Europe when the SDI value was 0.73 (Supplementary Figures 4A,B).

**Figure 5:**
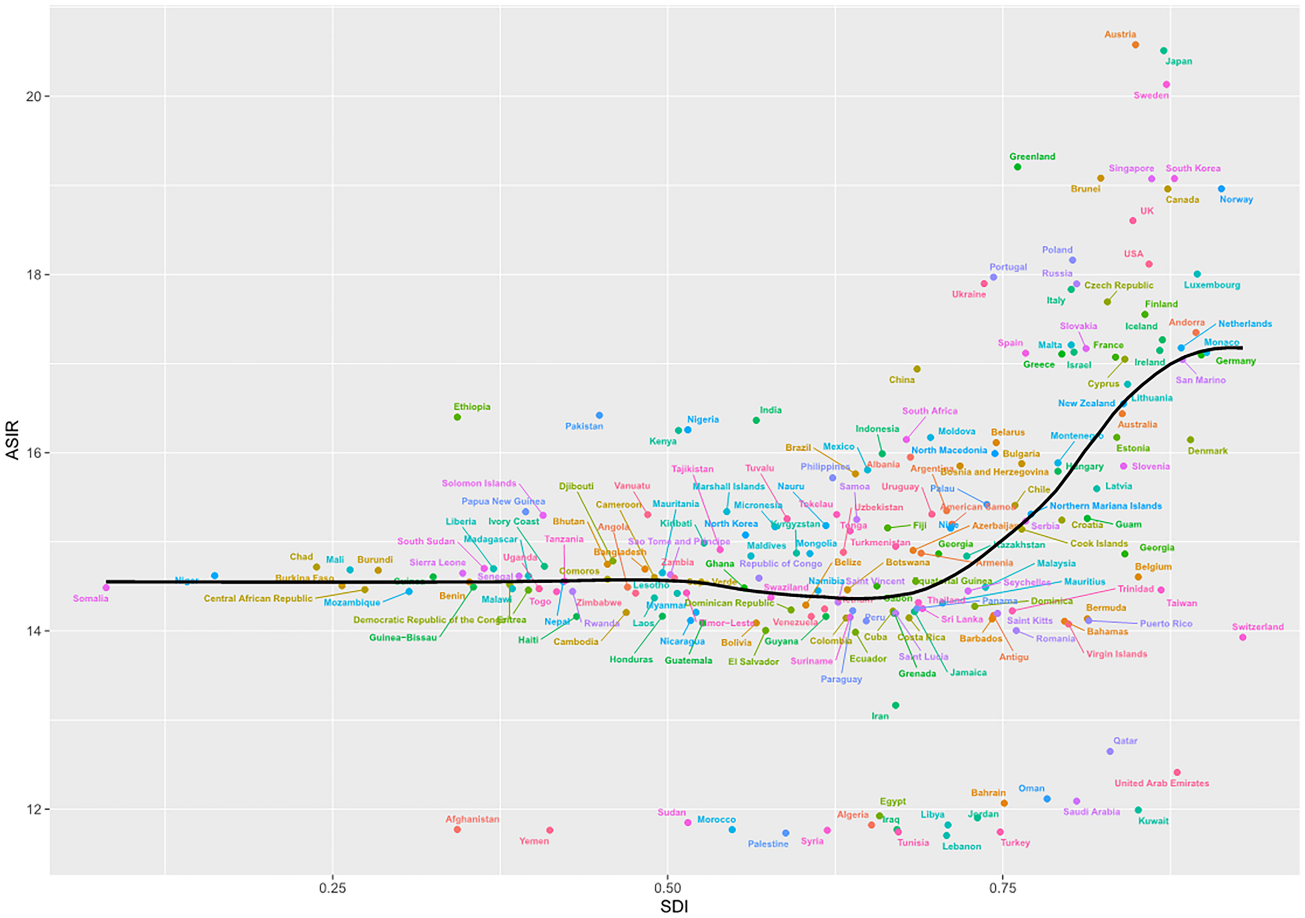
ASIR of cardiomyopathy and myocarditis in 204 countries by SDI, 1990–2019. ASIR, age-standardized incidence rate; SDI, sociodemographic index.

A non-linear relationship between ASIR and SDI values was observed at the national level in 2019. Based on SDI, Austria had the highest ASIR, followed by Japan and Sweden. The ASIR was higher than expected in most countries. While Afghanistan, Yemen, Palestine, Turkey, Lebanon, and many other countries had lower ASIR than the expected SDI (Figure 5). While based on SDI, Montenegro had the highest ASMR, followed by Kazakhstan and Russia, but most countries had lower ASMR than expected. Russia had the highest ASDR, followed by Ukraine and Latvia, but most countries had lower ASMR than expected. Interestingly, with the increase of SDI, ASIR gradually increased, but ASMR and ASDR decreased until SDI was about 0.75 (Supplementary Figures 5A,B).

### Risk factors of CM-MC

**Figure 6:**
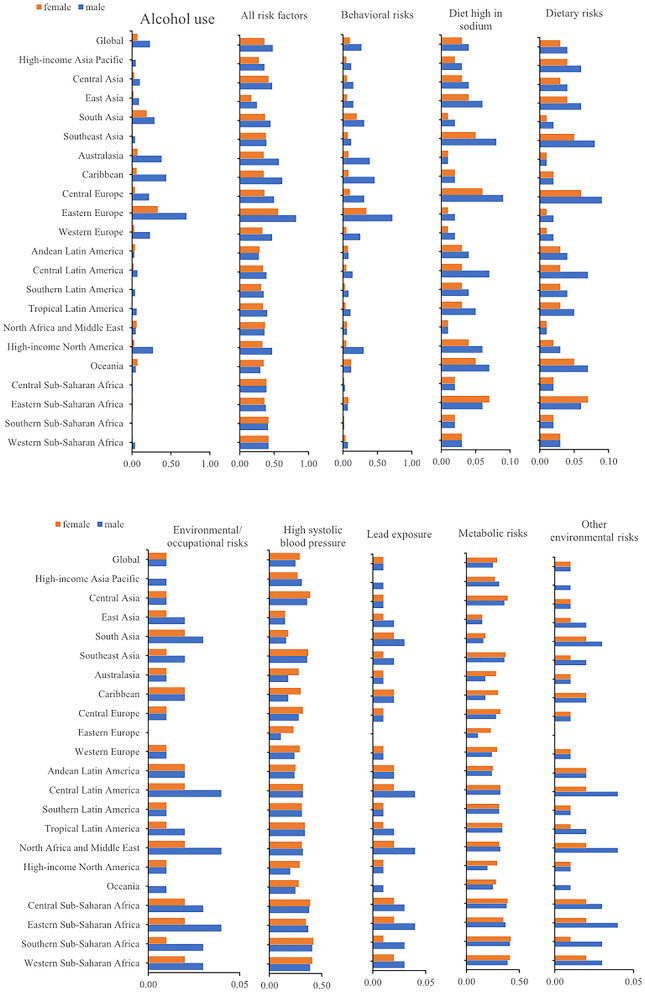
The age-standardized deaths attributable to risk factors.

Globally, there were 185,308 (154,610–200,448) deaths from CM-MC. The 2019 rates of CM-MC deaths could be attributed to the following GBD-estimated risk factors: alcohol use, behavioral risks, a diet high in sodium, dietary risks, environmental/occupational risks, high systolic blood pressure (SBP), lead exposure, metabolic risks and other environmental risks. These risk factors showed different proportions according to gender in each region. For example, in Southern Sub-Saharan Africa, males (41%) had the highest death rate due to high SBP. Meanwhile, East Asia had the lowest death rate due to high SBP in females (15%). In 2019, the highest proportion of deaths due to behavioral risks were in Eastern Europe (male, 72%; female, 34%). Regarding alcohol use, the highest percentage of deaths due to it was observed in Eastern Europe 70% for males and 33% for females (Figure 6).

## Discussion

CM-MC is a common chronic non-communicable disease, and about 12–17% of adults with CM-MC cause heart failure, with a mortality rate of about 8.4 deaths per 100,000 (20). And with the acceleration of population aging, unexplained myocardial degenerative lesions occur during the aging process. And CM-MC remains a significant burden on the elderly; we need to know more about the risk factors for CM-MC, which is particularly important for its prevention and treatment.

The current study estimated the number of CM-MC, morbidity, mortality, DALYs, ASR, EAPC and risk factors in 204 countries and 21 regions from 1990 to 2019. Firstly, during this period, morbidity and mortality increased in different countries. The number of morbidities, mortality and DALYs in 2019 were 470 thousand, 180 thousand and 3 million, respectively. Secondly, the global ASMR of CM-MC decreased (EAPC was −1.63%); ASDR and ASIR declined less (−1.2% for EAPC; −0.23% for EAPC). An increase in the number of cases will inevitably increase the disease burden and affect the quality of care. Although the global ASIR, ASMR and ASDR were decreasing; however, the ASMR was still increasing in some countries, such as Kazakhstan, with an EAPC of 10.31%, indicating that the burden of CM-MC was still high in some countries.

In the above section, we analyzed the correlation between EAPC and HDI. HDI is an essential index to assess a country's socioeconomic development level (21, 22). In this paper, a non-linear correlation analysis was performed between EAPCs and HDI, and the results showed a positive correlation between EAPCs and HDI when HDI was restricted to < 0.7. On the contrary, when the HDI value was higher than 0.7, there was a negative correlation between EAPCs and HDI. Some factors may influence the above findings. Potential differences exist between populations in different regions, including living environment and dietary habits. Moreover, people with higher education and per capita income have higher health literacy and will pay more attention to their health (23–26). The analysis revealed certain patterns of morbidity, mortality, and DALYs varying across SDI regions. In 2019, morbidity and mortality were highest in high SDI regions or high-moderate SDI regions, while the opposite was true for low or low-moderate SDI regions. However, the most significant increases could be detected in low, medium and medium SDI regions. This finding suggests that CM-MC is associated with regional development. Prevention and diagnosis of CM-MC may become relatively difficult due to lack of medical resources, leading to increased mortality and national disease burden.

This study found that high SBP and alcohol consumption were major contributors to the burden of CM-MC. Mahfoud et al. (27) found that blood pressure is a predictor of cardiac death or heart transplantation in patients with myocarditis. With age, vascular wall compliance decreases and vascular resistance increases, making the body unable to buffer against blood pressure fluctuations (28, 29). All of these contribute to the predisposition of the elderly to hypertension with high SBP alone. To prevent hypertension, less salt intake and home blood-pressure monitoring should be routine in daily management. Moreover, long-term heavy alcohol consumption causes the heart and vascular smooth muscle to lose elasticity. And heavy alcohol consumption accounts for about one-third of all causes of non-ischemic dilated cardiomyopathy and is a common cause of atrial fibrillation (30). Considering the critical impact of high SBP and alcohol consumption as causative factors for cardiomyopathy and myocarditis, monitoring blood pressure and alcohol intake remains critical for the development of targeted CM-MC prevention strategies. By analyzing risk and mortality factors in each region, prevention and monitoring measures relevant to that region can then be developed. For example, in Eastern Europe, where two Eastern European countries had high mortality rates due to alcohol consumption, monitoring alcohol consumption and controlling alcohol intake can be used for CM-MC prevention in Eastern Europe. Regional and temporal differences exist due to differences in risk factors (20), and each region can tailor prevention to its own top risk factors.

Although this GBD analysis filled a gap where CM-MC burden data are scarce or unavailable, there are still some limitations. First, data availability is low in some countries, although statistically robust methods have been used to overcome data scarcity and address uncertainty. In addition, the quality of data collection and data sources varies from country to country, and delays and inaccuracies reported in this study are inevitable, although GBD has attempted to improve the quality of data reliability. Finally, diagnostic criteria may change over time.

In conclusion, CM-MC remains a significant cause of early death and chronic disability from 1990 to 2019 and negatively impacts the global disease burden. As such, CM-MC is a major global public health problem. Worryingly, despite advances in technical capacity to prevent and treat CVD, the decline in CM-MC mortality is not evident in many parts of the world. Moreover, the burden of all these diseases varies greatly between countries and regions. Based on this study, public health agencies should develop and implement more effective strategies to address and solve these problems and risk factors.

## Data availability statement

The original contributions presented in the study are included in the article/Supplementary material, further inquiries can be directed to the corresponding authors.

## Author contributions

KZ, XC, and NQ analyzed the data and performed the statistical analyses. KZ drafted the manuscript. NH and CK conceived and designed the study, participated in the revision, and final approval of the manuscript. HS, YL, TY, QX, and HT participated in partial data collection, data curation, and analysis. All authors contributed to the article and approved the submitted version.

## Funding

This study was supported by Grants from the National Natural Science Foundation of China (81870593 and 82170865), Natural Science Foundation of Shandong Province of China (ZR2020MH106), Quality Improvement of Postgraduate Education in Shandong Province (SDYAL19156) and Yuandu Scholars (2021).

## Conflict of interest

The authors declare that the research was conducted in the absence of any commercial or financial relationships that could be construed as a potential conflict of interest.

## Publisher's note

All claims expressed in this article are solely those of the authors and do not necessarily represent those of their affiliated organizations, or those of the publisher, the editors and the reviewers. Any product that may be evaluated in this article, or claim that may be made by its manufacturer, is not guaranteed or endorsed by the publisher.

## Supplementary material

Supplementary Figure 1 The EAPCs of CM-MC ASRs from 1990 to 2019, both sexes by SDI. SDI, sociodemographic index.

Supplementary Figure 2 The regional burden of CM-MC for both sexes in 2019. **(A)** ASIR in regions, 1990–2019; **(B)** ASMR in regions, 1990–2019; **(C)** ASDR in regions, 1990-2019. ASIR, age-standardized incidence rate; ASMR, age-standardized mortality rate; ASDR, age-standardized DALYs rate.

Supplementary Figure 3 The EAPCs of CM-MC at regional level, from 1990 to 2019.

Supplementary Figure 4 ASRs of CM-MC in global and 21 regions by SDI, 1990–2019. **(A)** ASMR in global and 21 regions, 1990–2019; **(B)** ASDR in global and 21 regions, 1990–2019. ASMR, age-standardized mortality rate; ASDR, age-standardized DALYs rate; SDI, sociodemographic index.

Supplementary Figure 5 ASRs of CM-MC in 204 countries by SDI, 1990–2019. **(A)** ASMR in 204 countries and territories, 2019; **(B)** ASDR in 204 countries and territories, 2019. ASMR, age-standardized mortality rate; ASDR, age-standardized DALYs rate; SDI, sociodemographic index.

Supplementary Table 1 The incidence of Cardiomyopathy and myocarditis between 1990 and 2019 at national level.

Supplementary Table 2 The deaths of Cardiomyopathy and myocarditis between 1990 and 2019 at national level.

Supplementary Table 3 The disability-adjusted life years (DALYs) of Cardiomyopathy and myocarditis between 1990 and 2019 at national level.
