# Supplementary figures and images for: Global Burden of cardiomyopathy and myocarditis in the older adults from 1990 to 2019
Source: Front Public Health. 2022 Sep 23;10:1018385. doi: 10.3389/fpubh.2022.1018385 (PMC9545016; doi:10.3389/fpubh.2022.1018385)

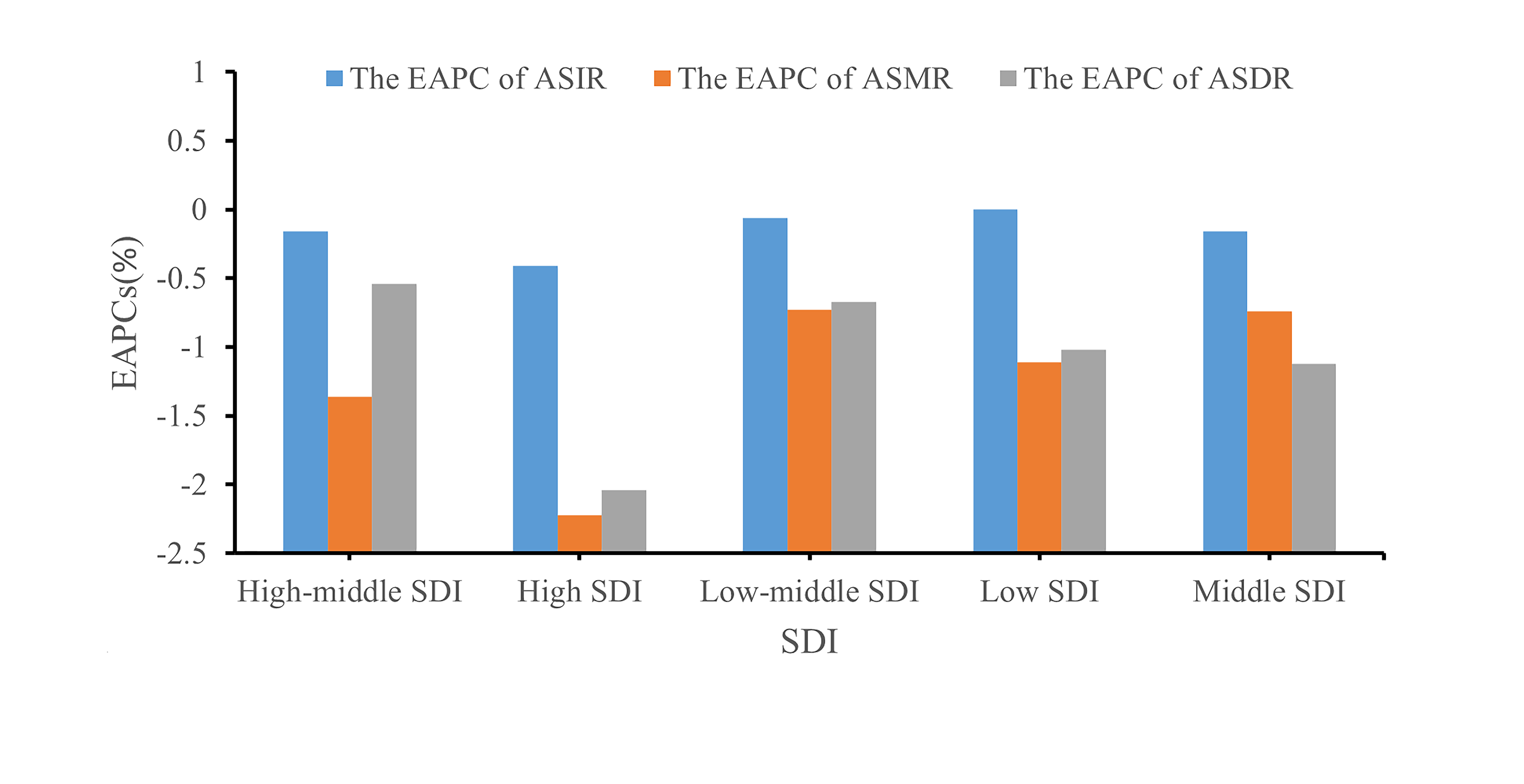

Supplement: Supplementary Figure 1 — The EAPCs of CM-MC ASRs from 1990 to 2019, both sexes by SDI. SDI, sociodemographic index. [file Image_1.TIF]

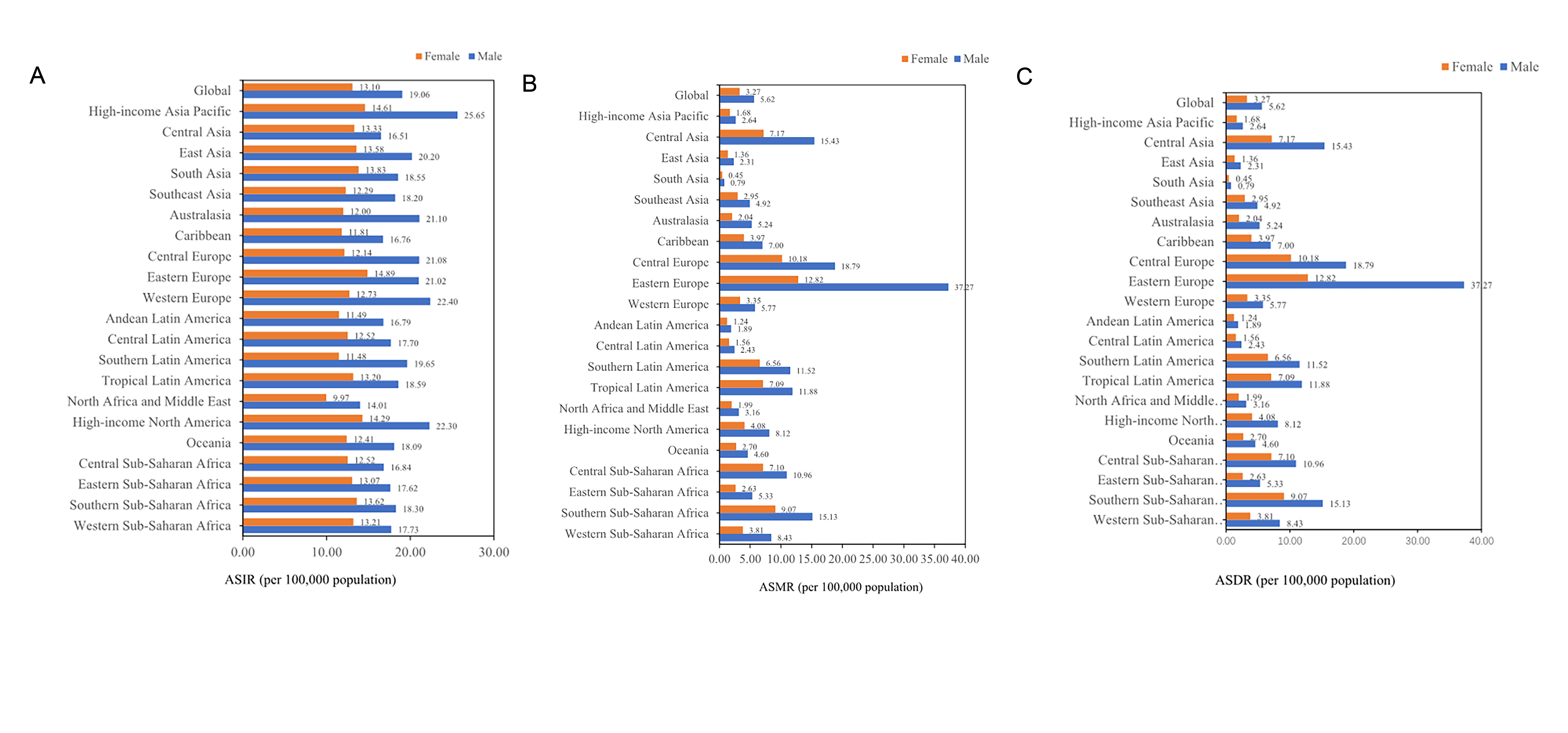

Supplement: Supplementary Figure 2 — The regional burden of CM-MC for both sexes in 2019. (A) ASIR in regions, 1990–2019; (B) ASMR in regions, 1990–2019; (C) ASDR in regions, 1990-2019. ASIR, age-standardized incidence rate; ASMR, age-standardized mortality rate; ASDR, age-standardized DALYs rate. [file Image_2.TIF]
