# Supplementary material for: Global Burden of cardiomyopathy and myocarditis in the older adults from 1990 to 2019
Source: Front Public Health. 2022 Sep 23;10:1018385. doi: 10.3389/fpubh.2022.1018385 (PMC9545016; doi:10.3389/fpubh.2022.1018385)
Supplement: Supplementary Table 1 — The incidence of Cardiomyopathy and myocarditis between 1990 and 2019 at national level. [file Table_1.docx]

**Supplementary Table 1. The incidence of Cardiomyopathy and myocarditis between 1990 and 2019 at national level.**

| location | Cases in 1990 | ASIR in 1990 | Cases in 2019 | ASIR in 2019 | Percentage change | EAPC (95%) |
| --- | --- | --- | --- | --- | --- | --- |
| Afghanistan | 272 (189.7-371.9) | 11.8 (9.5-14.3) | 355.3 (249.6-479.6) | 11.8 (9.5-14.4) | 0.3% (0.3-0.4) | -0.01 (-0.01-0) |
| Albania | 91 (63.6-122.8) | 15.9 (12.8-19.4) | 231.3 (160.1-311.4) | 16 (12.8-19.4) | 1.5% (1.4-1.7) | 0.01 (0-0.03) |
| Algeria | 459.1 (315.6-633.1) | 11.8 (9.5-14.4) | 1284.4 (911.5-1727.5) | 11.8 (9.5-14.4) | 1.8% (1.7-2) | 0.01 (0.01-0.01) |
| American Samoa | 1 (0.7-1.3) | 15.3 (12.3-18.4) | 2.2 (1.6-3) | 15.2 (12.2-18.4) | 1.3% (1.3-1.4) | -0.02 (-0.02 to -0.02) |
| Andorra | 3.3 (2.3-4.6) | 17.5 (14.2-21.3) | 8.3 (5.9-11.3) | 17.3 (14-21.1) | 1.5% (1.4-1.7) | -0.04 (-0.04 to -0.03) |
| Angola | 153.9 (108-209.9) | 14.6 (11.8-17.9) | 436.6 (309.5-593.2) | 14.5 (11.7-17.7) | 1.8% (1.8-1.9) | -0.04 (-0.04 to -0.03) |
| Antigua and Barbuda | 3.1 (2.2-4.2) | 14.1 (11.4-17) | 5.2 (3.7-7) | 14.2 (11.5-17.1) | 0.7% (0.6-0.8) | 0.03 (0.03-0.04) |
| Argentina | 1955.3 (1386.8-2627.4) | 15.3 (12.4-18.6) | 3359.6 (2370-4513) | 15.3 (12.4-18.6) | 0.7% (0.7-0.8) | 0 (0-0.01) |
| Armenia | 119 (84.6-159.5) | 14.9 (11.9-18) | 213.1 (149.9-285.3) | 14.9 (11.9-18) | 0.8% (0.7-0.9) | 0.01 (0-0.01) |
| Australia | 1181.2 (825.5-1600.3) | 16.4 (13.1-19.8) | 2564.2 (1781.4-3473.4) | 16.4 (13.2-19.9) | 1.2% (1.1-1.2) | 0.02 (0.02-0.02) |
| Austria | 923.7 (658.3-1216.5) | 20.8 (17.3-24.7) | 1302.5 (933.8-1707.1) | 20.6 (17-24.8) | 0.4% (0.3-0.5) | -0.06 (-0.08 to -0.04) |
| Azerbaijan | 211.6 (150.6-283.4) | 14.8 (11.8-17.9) | 376.5 (266.9-507.6) | 14.9 (11.9-18) | 0.8% (0.7-0.8) | 0.03 (0.02-0.03) |
| Bahamas | 7.4 (5.2-10.1) | 14.1 (11.4-17) | 19 (13.6-25.5) | 14.1 (11.4-17.1) | 1.6% (1.5-1.6) | 0.01 (0.01-0.02) |
| Bahrain | 5.4 (3.8-7.4) | 12 (9.6-14.6) | 29.7 (20.5-40.7) | 12.1 (9.7-14.7) | 4.5% (4.2-4.9) | 0.04 (0.03-0.05) |
| Bangladesh | 2208.1 (1580.3-2962.5) | 14.8 (11.9-18.1) | 6685.1 (4771.6-8999.8) | 14.7 (11.8-18) | 2% (2-2.1) | -0.03 (-0.04 to -0.03) |
| Barbados | 17.8 (12.3-24) | 14 (11.4-17) | 28.2 (19.8-38) | 14.1 (11.4-17.1) | 0.6% (0.5-0.7) | 0.02 (0.02-0.02) |
| Belarus | 695.8 (493.7-942.4) | 16.1 (12.9-19.4) | 885.5 (629.2-1198.7) | 16.1 (13-19.5) | 0.3% (0.3-0.3) | 0.01 (0.01-0.01) |
| Belgium | 838.1 (580.1-1129.3) | 14.5 (11.7-17.6) | 1242.6 (858-1670.7) | 14.6 (11.8-17.7) | 0.5% (0.4-0.5) | -0.01 (-0.06-0.04) |
| Belize | 5 (3.5-6.7) | 14.3 (11.6-17.3) | 13.2 (9.4-17.8) | 14.3 (11.6-17.3) | 1.7% (1.6-1.7) | 0 (0-0) |
| Benin | 97.5 (69.6-131.7) | 14.6 (11.7-17.8) | 213 (152.2-286.6) | 14.5 (11.7-17.7) | 1.2% (1.1-1.2) | -0.01 (-0.01-0) |
| Bermuda | 3.3 (2.3-4.5) | 14.1 (11.4-17) | 7.5 (5.2-10.2) | 14.1 (11.4-17.1) | 1.3% (1.2-1.3) | 0.02 (0.01-0.02) |
| Bhutan | 10.2 (7.2-13.9) | 14.7 (11.8-17.9) | 27.8 (19.8-37.4) | 14.7 (11.8-18) | 1.7% (1.6-1.9) | 0.03 (0.02-0.03) |
| Bolivia (Plurinational State of) | 155 (110.1-211.1) | 14 (11.3-17.1) | 454.3 (322.2-618.4) | 14.1 (11.4-17.2) | 1.9% (1.9-2) | 0.02 (0.02-0.02) |
| Bosnia and Herzegovina | 174.5 (123.2-234.7) | 15.9 (12.7-19.3) | 319 (221.3-430) | 15.9 (12.8-19.3) | 0.8% (0.8-0.9) | 0.01 (0-0.02) |
| Botswana | 25.9 (18.3-35.1) | 14.4 (11.6-17.6) | 57.8 (41.2-78.1) | 14.5 (11.6-17.6) | 1.2% (1.2-1.3) | 0 (0-0) |
| Brazil | 4805.4 (3421.3-6600.5) | 15.8 (12.7-19.2) | 13748.2 (9749-18676.8) | 15.8 (12.7-19.2) | 1.9% (1.8-1.9) | -0.01 (-0.01 to -0.01) |
| Brunei Darussalam | 4.4 (3.1-6) | 19.1 (15.5-23.1) | 13.7 (9.6-18.5) | 19.1 (15.4-23.1) | 2.1% (2-2.2) | 0 (0-0.01) |
| Bulgaria | 642.9 (447.6-875.2) | 15.9 (12.8-19.4) | 818.5 (569.5-1104.5) | 15.9 (12.8-19.4) | 0.3% (0.2-0.4) | 0 (0-0.01) |
| Burkina Faso | 203.8 (143.7-276.4) | 14.5 (11.7-17.7) | 394.4 (283-529.1) | 14.5 (11.7-17.7) | 0.9% (0.9-1) | -0.01 (-0.01 to -0.01) |
| Burundi | 109.1 (77.7-147.8) | 14.5 (11.7-17.7) | 191.4 (135.9-258.3) | 14.7 (11.8-17.9) | 0.8% (0.7-0.8) | 0.04 (0.04-0.04) |
| Cabo Verde | 13 (9.1-17.5) | 14.4 (11.6-17.6) | 20.3 (14.4-27.1) | 14.5 (11.7-17.7) | 0.6% (0.5-0.6) | 0.03 (0.03-0.04) |
| Cambodia | 180.1 (127.4-244.3) | 14.1 (11.4-17.1) | 498.4 (354.5-674) | 14.2 (11.4-17.2) | 1.8% (1.7-1.8) | 0.02 (0.02-0.02) |
| Cameroon | 197.4 (141.8-265) | 14.6 (11.7-17.8) | 510.2 (366.8-684.5) | 14.6 (11.7-17.8) | 1.6% (1.6-1.6) | 0 (0-0) |
| Canada | 2437.5 (1720.6-3308.9) | 18.9 (15.3-23) | 5266.2 (3709.8-7119.6) | 19 (15.4-23.1) | 1.2% (1.1-1.2) | 0.01 (0.01-0.02) |
| Central African Republic | 46.3 (32.1-63.4) | 14.5 (11.7-17.7) | 79.1 (55.6-107.9) | 14.5 (11.6-17.6) | 0.7% (0.7-0.7) | -0.01 (-0.01 to -0.01) |
| Chad | 139.6 (99.1-189.5) | 14.6 (11.7-17.8) | 251.3 (180.2-337.4) | 14.7 (11.8-18) | 0.8% (0.7-0.8) | 0.04 (0.04-0.04) |
| Chile | 573.7 (407.5-770.7) | 15.3 (12.5-18.6) | 1469 (1039.6-1965.9) | 15.4 (12.5-18.7) | 1.6% (1.5-1.6) | 0.02 (-0.02-0.05) |
| China | 46009.7 (32075.7-62947.5) | 18 (14.6-22.1) | 122265.5 (85925.2-166094.9) | 16.9 (13.8-20.5) | 1.7% (1.5-1.8) | -0.29 (-0.33 to -0.24) |
| Colombia | 847.1 (603.6-1141.2) | 14.2 (11.5-17.2) | 2838.4 (1986.8-3813) | 14.1 (11.4-17.1) | 2.4% (2.2-2.5) | -0.02 (-0.02 to -0.02) |
| Comoros | 10.8 (7.7-14.6) | 14.6 (11.7-17.8) | 23.1 (16.5-31.1) | 14.6 (11.7-17.8) | 1.1% (1.1-1.2) | 0 (-0.01-0) |
| Congo | 46.8 (32.9-63.4) | 14.5 (11.7-17.7) | 105.8 (75.8-142.1) | 14.6 (11.7-17.8) | 1.3% (1.2-1.3) | 0.03 (0.03-0.03) |
| Cook Islands | 0.6 (0.4-0.8) | 15.3 (12.4-18.6) | 1.3 (0.9-1.8) | 15.1 (12.2-18.3) | 1.2% (1.1-1.3) | -0.05 (-0.05 to -0.04) |
| Costa Rica | 90.4 (63.4-121.9) | 14.2 (11.5-17.2) | 272.1 (192.1-365.8) | 14.1 (11.5-17.1) | 2% (2-2) | -0.02 (-0.02 to -0.02) |
| Croatia | 312.9 (215-422.6) | 15.3 (12.1-18.6) | 481.4 (338-650.1) | 15.2 (12.4-18.4) | 0.5% (0.4-0.7) | -0.07 (-0.14-0) |
| Cuba | 581.3 (404.3-789.2) | 14.3 (11.6-17.3) | 1043.7 (728-1413.3) | 14.2 (11.5-17.2) | 0.8% (0.8-0.8) | -0.01 (-0.01 to -0.01) |
| Cyprus | 50 (34.7-68.6) | 17.1 (13.9-20.9) | 123.5 (86-167.4) | 17.1 (13.9-20.8) | 1.5% (1.4-1.6) | 0.03 (-0.01-0.07) |
| Czechia | 795.8 (547.2-1071.5) | 17.5 (14.1-21.4) | 1262.6 (868.4-1715.3) | 17.7 (14.2-21.6) | 0.6% (0.5-0.6) | 0.01 (-0.01-0.02) |
| C么te d'Ivoire | 159.5 (112.2-216.8) | 14.8 (11.9-18) | 435.1 (311.2-585.2) | 14.7 (11.8-18) | 1.7% (1.7-1.8) | 0 (-0.01-0) |
| Democratic People's Republic of Korea | 651 (465.7-884.6) | 14.8 (12-18) | 1587.3 (1110.3-2134) | 15.1 (12.2-18.3) | 1.4% (1.3-1.6) | 0.06 (0.06-0.06) |
| Democratic Republic of the Congo | 671.4 (469.7-920.5) | 14.6 (11.7-17.8) | 1447.3 (1038.3-1952.7) | 14.5 (11.7-17.7) | 1.2% (1.1-1.3) | -0.02 (-0.02 to -0.01) |
| Denmark | 483.7 (334.9-650.8) | 16 (13-19.3) | 691.7 (479.2-929.7) | 16.1 (13.1-19.5) | 0.4% (0.4-0.4) | 0.03 (-0.01-0.06) |
| Djibouti | 5.2 (3.6-7.1) | 14.7 (11.8-18) | 24 (17-32.6) | 14.8 (11.9-18.1) | 3.6% (3.6-3.7) | 0.01 (0.01-0.02) |
| Dominica | 4.3 (3-5.9) | 14.1 (11.4-17) | 5.1 (3.6-6.9) | 14.3 (11.5-17.3) | 0.2% (0.2-0.2) | 0.06 (0.06-0.06) |
| Dominican Republic | 186.9 (132-252.4) | 14.2 (11.5-17.2) | 481.6 (338.2-648.6) | 14.2 (11.5-17.2) | 1.6% (1.5-1.6) | 0.01 (0-0.01) |
| Ecuador | 270 (192.8-358) | 13.9 (11.3-16.8) | 799 (568.6-1075.4) | 14 (11.4-16.8) | 2% (1.8-2.1) | 0.04 (0.01-0.06) |
| Egypt | 1028.4 (719.1-1398.9) | 11.8 (9.5-14.4) | 2255.8 (1575-3068.8) | 11.9 (9.6-14.5) | 1.2% (1.1-1.2) | 0.04 (0.04-0.04) |
| El Salvador | 149.9 (105.7-202) | 14.1 (11.5-17.1) | 319.6 (223-432.1) | 14 (11.3-16.9) | 1.1% (1.1-1.2) | -0.04 (-0.04 to -0.03) |
| Equatorial Guinea | 8.4 (6-11.4) | 14.4 (11.6-17.6) | 20.1 (14.4-26.9) | 14.6 (11.7-17.7) | 1.4% (1.3-1.5) | 0.04 (0.03-0.05) |
| Eritrea | 34.1 (23.8-46.7) | 14.4 (11.6-17.6) | 99.9 (70.7-135.6) | 14.5 (11.6-17.6) | 1.9% (1.9-2) | 0.01 (0.01-0.02) |
| Estonia | 112.1 (79.4-152.1) | 16.1 (12.9-19.4) | 155.6 (108.6-210.6) | 16.2 (13-19.5) | 0.4% (0.3-0.4) | 0.03 (0.02-0.03) |
| Eswatini | 12.4 (8.8-16.7) | 14.4 (11.6-17.5) | 25.3 (17.8-34.2) | 14.4 (11.5-17.5) | 1% (1-1.1) | 0 (-0.01-0) |
| Ethiopia | 943.5 (666.6-1302.3) | 16.3 (13.2-19.8) | 2082 (1488.2-2831) | 16.4 (13.3-19.8) | 1.2% (1.1-1.4) | 0.01 (0-0.01) |
| Fiji | 14.3 (10-19.6) | 15.2 (12.3-18.4) | 32.7 (22.9-44.8) | 15.2 (12.2-18.3) | 1.3% (1.3-1.3) | -0.01 (-0.02 to -0.01) |
| Finland | 441.6 (313.5-599) | 17.3 (14-20.8) | 801.9 (560.5-1084) | 17.6 (14.2-21.2) | 0.8% (0.8-0.9) | 0.03 (-0.02-0.08) |
| France | 5234.9 (3685-7129.6) | 17 (13.7-20.7) | 8517.4 (5983.2-11542) | 17.1 (13.8-20.8) | 0.6% (0.6-0.7) | 0.02 (0.02-0.02) |
| Gabon | 26.7 (19-36.1) | 14.5 (11.7-17.7) | 46.1 (33.1-61.6) | 14.5 (11.7-17.7) | 0.7% (0.7-0.8) | 0 (0-0) |
| Gambia | 15.8 (11.2-21.4) | 14.7 (11.8-17.9) | 44.6 (31.8-60) | 14.6 (11.7-17.8) | 1.8% (1.7-2) | -0.02 (-0.03 to -0.02) |
| Georgia | 300.9 (213.2-407.9) | 14.8 (11.8-17.9) | 314.8 (223.1-425.5) | 14.9 (12-18.1) | 0% (0-0.1) | 0.01 (0.01-0.01) |
| Germany | 7723.8 (5398.9-10379.7) | 16.8 (13.7-20.5) | 12214.1 (8561.3-16560.1) | 17.1 (13.9-20.8) | 0.6% (0.5-0.6) | 0.04 (0.04-0.05) |
| Ghana | 270.7 (192.3-364.7) | 14.6 (11.7-17.8) | 703.9 (505.8-946.3) | 14.5 (11.7-17.7) | 1.6% (1.6-1.6) | -0.03 (-0.03 to -0.03) |
| Greece | 960.5 (679.4-1312.6) | 17.1 (13.9-20.8) | 1534.1 (1072.9-2074.8) | 17.1 (13.9-20.8) | 0.6% (0.5-0.7) | 0 (-0.01-0) |
| Greenland | 1.9 (1.3-2.6) | 19.2 (15.6-23.5) | 4.6 (3.3-6.3) | 19.2 (15.6-23.5) | 1.4% (1.4-1.5) | 0 (-0.01-0) |
| Grenada | 4.3 (3-5.8) | 14.1 (11.4-17) | 5.6 (4-7.7) | 14.2 (11.5-17.2) | 0.3% (0.2-0.4) | 0.02 (0.01-0.03) |
| Guam | 3.3 (2.3-4.5) | 15.3 (12.4-18.6) | 9.5 (6.7-12.9) | 15.3 (12.3-18.5) | 1.9% (1.7-2.1) | -0.02 (-0.03 to -0.01) |
| Guatemala | 171.2 (120.7-231.1) | 14.3 (11.6-17.3) | 582.9 (404.9-788.1) | 14.1 (11.4-17) | 2.4% (2.2-2.7) | -0.05 (-0.05 to -0.04) |
| Guinea | 165.6 (118-223.3) | 14.6 (11.8-17.9) | 259.1 (184.9-348) | 14.6 (11.7-17.8) | 0.6% (0.5-0.6) | 0 (-0.01-0) |
| Guinea-Bissau | 18.2 (12.8-24.6) | 14.6 (11.7-17.8) | 29.5 (20.9-39.7) | 14.5 (11.7-17.7) | 0.6% (0.6-0.6) | -0.02 (-0.02 to -0.01) |
| Guyana | 18.3 (13.1-24.7) | 14.2 (11.5-17.2) | 29.8 (21.2-40.2) | 14.2 (11.5-17.1) | 0.6% (0.6-0.7) | -0.01 (-0.01-0) |
| Haiti | 151.2 (106.7-205) | 14.2 (11.4-17.1) | 319.3 (226.7-430.8) | 14.2 (11.5-17.1) | 1.1% (1.1-1.2) | 0 (0-0.01) |
| Honduras | 101 (71.6-136) | 14.2 (11.5-17.2) | 303.2 (214.5-410.7) | 14.2 (11.5-17.1) | 2% (2-2) | -0.01 (-0.02 to -0.01) |
| Hungary | 771.5 (533.3-1036.4) | 15.8 (12.7-19.2) | 1056.2 (734.3-1423.4) | 15.8 (12.7-19.3) | 0.4% (0.3-0.4) | 0.01 (0.01-0.01) |
| Iceland | 17.7 (12.5-24.2) | 17.2 (13.9-20.9) | 34.4 (24.2-46.9) | 17.3 (14-21) | 0.9% (0.9-1) | 0.02 (0.02-0.03) |
| India | 21270.5 (15169.5-29093.9) | 16.5 (13.3-19.9) | 62331 (44677.4-85093.5) | 16.4 (13.3-19.8) | 1.9% (1.8-2.1) | -0.02 (-0.02 to -0.02) |
| Indonesia | 4341.4 (3068.8-5851.7) | 16 (12.9-19.4) | 9936.9 (7085.9-13348.9) | 16 (13-19.4) | 1.3% (1.3-1.3) | 0 (0-0) |
| Iran (Islamic Republic of) | 1002.1 (697-1388) | 13.2 (10.6-16.1) | 3043.8 (2182.4-4091.5) | 13.2 (10.6-16.1) | 2% (1.8-2.4) | 0 (-0.01-0) |
| Iraq | 284.7 (202.3-382.5) | 11.8 (9.5-14.3) | 778.2 (547.5-1053) | 11.8 (9.5-14.3) | 1.7% (1.6-1.8) | 0 (-0.01-0) |
| Ireland | 258.1 (181.1-351.5) | 17.1 (13.8-20.8) | 465.9 (327.6-635.3) | 17.1 (13.9-20.9) | 0.8% (0.8-0.8) | 0.02 (0.01-0.02) |
| Israel | 307.8 (215.9-418.2) | 17.1 (13.8-20.8) | 715.6 (506.6-978.5) | 17.1 (13.9-20.9) | 1.3% (1.2-1.4) | 0.01 (0.01-0.02) |
| Italy | 6210.1 (4342.4-8452.2) | 19 (15.3-23.2) | 8781.8 (6172.2-11749.6) | 17.8 (14.7-21.3) | 0.4% (0.3-0.5) | -0.34 (-0.42 to -0.27) |
| Jamaica | 102.5 (71.4-139) | 14.2 (11.5-17.1) | 158.9 (111.5-213.3) | 14.2 (11.5-17.2) | 0.5% (0.5-0.6) | 0.01 (0.01-0.02) |
| Japan | 11404.1 (7987.3-15522.5) | 21.2 (17.1-25.8) | 23888.4 (16369.3-32356.1) | 20.5 (16.7-24.6) | 1.1% (0.9-1.3) | -0.25 (-0.3 to -0.21) |
| Jordan | 42.8 (30.2-58.1) | 11.8 (9.5-14.4) | 218.6 (152.9-297.3) | 11.9 (9.6-14.5) | 4.1% (4-4.3) | 0.02 (0.01-0.02) |
| Kazakhstan | 567.5 (404.6-769.2) | 14.8 (11.9-17.9) | 761.3 (545.4-1032) | 14.8 (11.9-17.9) | 0.3% (0.3-0.4) | 0 (0-0) |
| Kenya | 420.5 (301.3-572.3) | 16.3 (13.2-19.8) | 1034.3 (739.1-1404.5) | 16.3 (13.1-19.7) | 1.5% (1.4-1.5) | -0.02 (-0.02 to -0.01) |
| Kiribati | 1.5 (1-2.1) | 15 (12.1-18.2) | 2.6 (1.8-3.6) | 15 (12.1-18.1) | 0.7% (0.7-0.8) | -0.01 (-0.01 to -0.01) |
| Kuwait | 18.2 (12.9-24.7) | 12.1 (9.7-14.7) | 77.3 (55.3-103) | 12 (9.7-14.6) | 3.2% (3.1-3.4) | -0.03 (-0.05 to -0.01) |
| Kyrgyzstan | 143.8 (101.2-192.8) | 14.8 (11.9-17.9) | 188 (134.8-250.1) | 14.9 (11.9-18) | 0.3% (0.3-0.3) | 0.01 (0.01-0.01) |
| Lao People's Democratic Republic | 87 (61.2-119.2) | 14.3 (11.5-17.3) | 178.2 (126.9-241) | 14.4 (11.5-17.4) | 1% (1-1.1) | 0.02 (0.02-0.03) |
| Latvia | 189.3 (133.9-254.4) | 15.5 (12.5-18.8) | 226.7 (156.8-305.7) | 15.6 (12.6-18.9) | 0.2% (0.2-0.3) | -0.08 (-0.15 to -0.01) |
| Lebanon | 86.4 (61.1-116.5) | 11.8 (9.5-14.3) | 216.3 (150.6-292.5) | 11.7 (9.4-14.2) | 1.5% (1.3-1.7) | -0.02 (-0.02 to -0.01) |
| Lesotho | 46.2 (32.9-62.3) | 14.4 (11.6-17.6) | 56.2 (39.8-76.1) | 14.4 (11.6-17.6) | 0.2% (0.2-0.2) | -0.01 (-0.01-0) |
| Liberia | 56.9 (40.6-77.2) | 14.7 (11.8-18) | 85.7 (61.5-114.9) | 14.7 (11.8-17.9) | 0.5% (0.5-0.6) | 0 (0-0) |
| Libya | 67.1 (47.8-89.7) | 11.9 (9.6-14.5) | 173.8 (123.2-233.6) | 11.8 (9.5-14.4) | 1.6% (1.5-1.7) | -0.01 (-0.02-0.01) |
| Lithuania | 257.7 (180.7-350.5) | 16.7 (13.5-20.3) | 351 (244.7-478.4) | 16.8 (13.5-20.4) | 0.4% (0.3-0.4) | -0.04 (-0.1-0.01) |
| Luxembourg | 34.4 (24-46.1) | 17.7 (14.3-21.4) | 62.5 (43.8-83.7) | 18 (14.6-21.7) | 0.8% (0.8-0.9) | -0.03 (-0.06-0.01) |
| Madagascar | 238.6 (171.2-319.4) | 14.7 (11.8-17.9) | 427.8 (302.4-579.2) | 14.6 (11.7-17.8) | 0.8% (0.7-0.8) | -0.02 (-0.02 to -0.02) |
| Malawi | 171.8 (121.4-233.1) | 14.6 (11.7-17.8) | 320.4 (228.1-431.3) | 14.5 (11.6-17.7) | 0.9% (0.8-0.9) | -0.03 (-0.03 to -0.03) |
| Malaysia | 390.8 (278.1-525.3) | 14.4 (11.6-17.4) | 1222.3 (868-1650.8) | 14.5 (11.6-17.5) | 2.1% (2.1-2.2) | 0.03 (0.02-0.03) |
| Maldives | 3.6 (2.5-4.9) | 14.7 (11.8-17.8) | 12.3 (8.7-16.4) | 14.8 (12-18) | 2.5% (2.2-2.8) | 0.03 (0.01-0.05) |
| Mali | 190.5 (135.2-256.9) | 14.6 (11.7-17.8) | 388.5 (278.9-521.4) | 14.7 (11.8-17.9) | 1% (1-1.1) | 0.02 (0.02-0.02) |
| Malta | 25.7 (18.3-34.8) | 17 (13.8-20.8) | 60.5 (42.5-82.3) | 17.2 (13.9-21) | 1.4% (1.3-1.4) | 0.03 (0.03-0.04) |
| Marshall Islands | 0.7 (0.5-1) | 15.2 (12.3-18.4) | 1.4 (0.9-1.9) | 15.3 (12.4-18.5) | 0.9% (0.7-1) | 0.04 (0.03-0.04) |
| Mauritania | 48 (34-65.1) | 14.6 (11.7-17.8) | 100.4 (71.4-134.5) | 14.7 (11.8-17.9) | 1.1% (1-1.2) | 0.02 (0.02-0.02) |
| Mauritius | 32.7 (23.2-44.3) | 14.3 (11.5-17.3) | 83.8 (60-113) | 14.3 (11.5-17.3) | 1.6% (1.5-1.6) | 0.01 (0.01-0.01) |
| Mexico | 2348.3 (1668.4-3198.6) | 15.9 (12.8-19.3) | 6700.8 (4732.5-9136.8) | 15.8 (12.7-19.3) | 1.9% (1.8-1.9) | -0.01 (-0.01 to -0.01) |
| Micronesia (Federated States of) | 2.1 (1.5-2.9) | 15.2 (12.3-18.4) | 2.8 (2-3.9) | 15.2 (12.2-18.3) | 0.3% (0.3-0.4) | -0.01 (-0.02 to -0.01) |
| Monaco | 4.6 (3.2-6.3) | 17 (13.8-20.7) | 6 (4.2-8.2) | 17.1 (13.9-20.9) | 0.3% (0.3-0.3) | 0.02 (0.01-0.03) |
| Mongolia | 46 (32.2-62.5) | 14.9 (11.9-18) | 85.4 (60.9-114.9) | 14.9 (11.9-18) | 0.9% (0.8-0.9) | 0 (0-0) |
| Montenegro | 29.7 (20.7-39.9) | 15.9 (12.8-19.3) | 52.4 (36.4-70.8) | 15.9 (12.8-19.4) | 0.8% (0.7-0.8) | 0.01 (0-0.01) |
| Morocco | 504.7 (354.5-687) | 11.7 (9.5-14.3) | 1152 (810.3-1553.6) | 11.8 (9.5-14.3) | 1.3% (1.2-1.3) | 0.01 (0.01-0.02) |
| Mozambique | 262.9 (186.4-354.7) | 14.5 (11.7-17.7) | 462.9 (330.8-623.9) | 14.4 (11.6-17.6) | 0.8% (0.7-0.8) | -0.02 (-0.02 to -0.02) |
| Myanmar | 980.7 (693.1-1332.6) | 14.3 (11.5-17.3) | 1993.2 (1421.9-2688.3) | 14.2 (11.4-17.2) | 1% (1-1.1) | -0.02 (-0.02 to -0.02) |
| Namibia | 35.8 (25.3-48.7) | 14.5 (11.7-17.7) | 64.8 (46-87.3) | 14.5 (11.6-17.6) | 0.8% (0.8-0.9) | -0.02 to (-0.02 to -0.01) |
| Nauru | 0.1 (0.1-0.2) | 15.3 (12.3-18.5) | 0.1 (0.1-0.2) | 15.2 (12.2-18.3) | 0% (-0.1-0) | -0.03 (-0.04 to -0.02) |
| Nepal | 408.8 (290-553.4) | 14.7 (11.8-17.9) | 1095 (780.1-1478.4) | 14.6 (11.7-17.8) | 1.7% (1.6-1.8) | -0.03 (-0.03 to -0.03) |
| Netherlands | 1223.4 (863.4-1662.5) | 17 (13.8-20.8) | 2167.8 (1522.1-2950.1) | 17.2 (13.9-20.9) | 0.8% (0.8-0.8) | 0.03 (0.03-0.04) |
| New Zealand | 249.9 (171.3-340.5) | 17.2 (13.7-21.3) | 454.8 (319-599) | 16.6 (13.8-19.6) | 0.8% (0.7-1) | -0.23 (-0.33 to -0.13) |
| Nicaragua | 72.5 (51.6-97.6) | 14.1 (11.4-17.1) | 214.9 (152.8-290) | 14.1 (11.4-17.1) | 2% (2-2) | 0 (0-0) |
| Niger | 119.4 (84.3-162.4) | 14.7 (11.8-17.9) | 335.6 (237.2-453.1) | 14.6 (11.8-17.8) | 1.8% (1.8-1.9) | -0.01 (-0.02 to -0.01) |
| Nigeria | 2273.3 (1632.6-3101.7) | 16.4 (13.3-19.9) | 4091.1 (2930.7-5540) | 16.3 (13.2-19.7) | 0.8% (0.8-0.8) | -0.03 (-0.04 to -0.02) |
| Niue | 0.1 (0.1-0.2) | 15.1 (12.2-18.3) | 0.1 (0.1-0.2) | 15.2 (12.2-18.3) | 0% (-0.1-0) | 0.01 (0-0.01) |
| North Macedonia | 86.2 (60.3-116.6) | 15.9 (12.8-19.4) | 165.4 (115.5-224.4) | 16 (12.9-19.5) | 0.9% (0.9-0.9) | 0.01 (0.01-0.01) |
| Northern Mariana Islands | 0.6 (0.4-0.8) | 15.4 (12.4-18.6) | 2.2 (1.5-3.1) | 15.3 (12.3-18.5) | 3% (2.8-3.1) | -0.02 (-0.04 to -0.01) |
| Norway | 481.2 (332.7-659.1) | 18.8 (15.1-23) | 651 (451.4-886.1) | 19 (15.2-23.3) | 0.4% (0.3-0.4) | -0.01 (-0.03-0.01) |
| Oman | 21 (14.7-28.7) | 12 (9.7-14.6) | 48.4 (34-66.3) | 12.1 (9.8-14.7) | 1.3% (1.3-1.3) | 0.05 (0.03-0.07) |
| Pakistan | 3113.7 (2218.1-4232.8) | 16.5 (13.4-20) | 5327.8 (3815.2-7244.2) | 16.4 (13.3-19.9) | 0.7% (0.6-0.8) | -0.03 (-0.03 to -0.03) |
| Palau | 0.5 (0.3-0.6) | 15.3 (12.3-18.5) | 0.9 (0.6-1.3) | 15.4 (12.4-18.6) | 1% (0.8-1) | 0.03 (0.01-0.04) |
| Palestine | 32.4 (22.9-43.7) | 11.7 (9.4-14.2) | 78.1 (55-105.5) | 11.7 (9.5-14.3) | 1.4% (1.4-1.4) | 0.01 (0.01-0.01) |
| Panama | 77.3 (54.3-104.4) | 14.3 (11.6-17.3) | 222.4 (155-301.2) | 14.3 (11.5-17.2) | 1.9% (1.8-1.9) | -0.01 (-0.01 to -0.01) |
| Papua New Guinea | 76.1 (52.8-105.2) | 15.3 (12.3-18.5) | 181.4 (126.3-249.7) | 15.3 (12.3-18.6) | 1.4% (1.3-1.4) | 0.01 (0.01-0.01) |
| Paraguay | 111.7 (79-150.8) | 14.2 (11.5-17.2) | 282.3 (200.8-379.7) | 14.2 (11.5-17.2) | 1.5% (1.5-1.5) | 0 (0-0) |
| Peru | 592.6 (421.6-799.5) | 14.1 (11.4-17.2) | 1715.9 (1211.1-2325) | 14.1 (11.4-17.2) | 1.9% (1.8-2) | 0 (0-0) |
| Philippines | 1380.2 (982-1858.9) | 16 (13-19.5) | 3533.7 (2525.4-4792.1) | 15.7 (12.6-19.1) | 1.6% (1.4-1.7) | -0.09 (-0.1 to -0.07) |
| Poland | 2484.5 (1759.9-3365.3) | 18 (14.5-21.9) | 4273.6 (3035.3-5771.5) | 18.2 (14.5-22.1) | 0.7% (0.7-0.8) | 0 (-0.06-0.06) |
| Portugal | 900.6 (634.8-1215.5) | 17.7 (14.4-21.6) | 1603.8 (1129.6-2137.7) | 18 (14.6-21.7) | 0.8% (0.7-0.9) | 0.06 (0.05-0.07) |
| Puerto Rico | 206.8 (143.3-280.2) | 14.1 (11.4-17.1) | 418.3 (292.2-567.7) | 14.1 (11.4-17.1) | 1% (1-1.1) | 0 (-0.01-0) |
| Qatar | 2.5 (1.8-3.5) | 12.3 (9.9-14.9) | 23.8 (15.9-33.2) | 12.6 (10.2-15.4) | 8.4% (7.6-8.9) | 0.13 (0.12-0.15) |
| Republic of Korea | 1428.8 (1001.3-1932.9) | 18.7 (15.2-22.7) | 5466.4 (3801.1-7370.1) | 19.1 (15.4-23.1) | 2.8% (2.6-3.1) | 0.06 (0.05-0.06) |
| Republic of Moldova | 223.2 (158.4-306.1) | 16.1 (12.9-19.4) | 319.3 (226.6-431.9) | 16.2 (13-19.5) | 0.4% (0.4-0.5) | 0.02 (0.02-0.02) |
| Romania | 1269 (885.9-1709) | 14 (11.2-17) | 1882.6 (1297.9-2534.3) | 14 (11.2-17) | 0.5% (0.4-0.6) | -0.13 (-0.17 to -0.08) |
| Russian Federation | 10452.7 (7464.6-14220.4) | 17.8 (14.3-21.6) | 14555.5 (10550.3-19730.5) | 17.9 (14.4-21.7) | 0.4% (0.4-0.4) | 0.02 (0.01-0.02) |
| Rwanda | 129.7 (91.6-175.3) | 14.5 (11.7-17.7) | 259 (184.5-351) | 14.4 (11.6-17.6) | 1% (1-1) | -0.02 (-0.02 to -0.01) |
| Saint Kitts and Nevis | 2.3 (1.6-3.2) | 14.1 (11.4-17) | 3.2 (2.3-4.3) | 14.2 (11.5-17.2) | 0.4% (0.3-0.5) | 0.03 (0.03-0.03) |
| Saint Lucia | 4.9 (3.4-6.6) | 14.1 (11.4-17) | 11.4 (8-15.4) | 14.2 (11.5-17.2) | 1.3% (1.3-1.4) | 0.03 (0.03-0.04) |
| Saint Vincent and the Grenadines | 4 (2.8-5.5) | 14.1 (11.4-17.1) | 7.5 (5.3-10.1) | 14.3 (11.6-17.3) | 0.8% (0.8-0.9) | 0.05 (0.05-0.05) |
| Samoa | 4.1 (2.9-5.6) | 15.3 (12.3-18.4) | 6.7 (4.7-9) | 15.3 (12.3-18.4) | 0.6% (0.6-0.7) | 0 (0-0) |
| San Marino | 2.1 (1.5-2.9) | 17.1 (13.9-20.9) | 4 (2.8-5.4) | 17 (13.8-20.7) | 0.9% (0.8-1) | -0.01 (-0.02 to -0.01) |
| Sao Tome and Principe | 3.3 (2.3-4.4) | 14.6 (11.7-17.8) | 4.6 (3.3-6.1) | 14.6 (11.8-17.9) | 0.4% (0.4-0.4) | 0.02 (0.02-0.02) |
| Saudi Arabia | 196.8 (138.7-266.9) | 12 (9.7-14.7) | 483.6 (338.7-659) | 12.1 (9.8-14.7) | 1.5% (1.3-1.6) | 0.01 (0.01-0.02) |
| Senegal | 152.9 (109.2-206.1) | 14.6 (11.8-17.9) | 347.5 (249-465.7) | 14.6 (11.7-17.8) | 1.3% (1.2-1.3) | 0 (0-0) |
| Serbia | 533.9 (379.2-713.4) | 15.2 (12.2-18.5) | 869.7 (605.6-1172.6) | 15.2 (12.2-18.6) | 0.6% (0.5-0.8) | -0.09 (-0.13 to -0.06) |
| Seychelles | 2.8 (2-3.8) | 14.3 (11.5-17.3) | 4.9 (3.5-6.5) | 14.4 (11.6-17.5) | 0.7% (0.7-0.8) | 0.05 (0.04-0.06) |
| Sierra Leone | 96 (67.9-129.9) | 14.7 (11.8-17.9) | 162 (115.4-218.4) | 14.6 (11.8-17.9) | 0.7% (0.6-0.7) | 0 (0-0.01) |
| Singapore | 115.7 (80.9-157.4) | 19 (15.4-23) | 462.3 (324.2-621.4) | 19.1 (15.5-23.1) | 3% (2.9-3.1) | 0.02 (0.02-0.03) |
| Slovakia | 331.9 (230.3-449.1) | 17.1 (13.8-20.8) | 523.9 (367.1-708.8) | 17.2 (13.8-20.8) | 0.6% (0.6-0.6) | -0.14 (-0.18 to -0.09) |
| Slovenia | 122.8 (85.6-165.1) | 15.7 (12.6-19) | 234 (163.7-317.2) | 15.9 (12.7-19.2) | 0.9% (0.9-1) | -0.01 (-0.03-0.02) |
| Solomon Islands | 5.4 (3.8-7.5) | 15.4 (12.5-18.7) | 11.5 (8-15.9) | 15.3 (12.3-18.5) | 1.1% (1.1-1.2) | -0.04 (-0.04 to -0.04) |
| Somalia | 93.5 (66.5-126.6) | 14.6 (11.7-17.8) | 263.6 (183.8-361.1) | 14.5 (11.6-17.7) | 1.8% (1.7-1.9) | -0.03 (-0.03 to -0.03) |
| South Africa | 1097.4 (792.3-1488.9) | 16.1 (13.1-19.6) | 2362.3 (1705.2-3201) | 16.1 (13.1-19.7) | 1.2% (1.1-1.2) | 0 (0-0.01) |
| South Sudan | 113.2 (80.7-152.9) | 14.8 (11.9-18.1) | 161 (115.8-215.4) | 14.7 (11.8-18) | 0.4% (0.4-0.4) | -0.03 (-0.03 to -0.02) |
| Spain | 3418.1 (2429.2-4655.1) | 17 (13.8-20.7) | 5924.4 (4141.4-7981.7) | 17.1 (13.9-20.9) | 0.7% (0.6-0.8) | 0.03 (0.03-0.03) |
| Sri Lanka | 473.3 (334.7-639.6) | 14.4 (11.6-17.5) | 1237.7 (876.4-1671.8) | 14.3 (11.4-17.2) | 1.6% (1.6-1.6) | -0.05 (-0.05 to -0.04) |
| Sudan | 348 (245.6-475) | 11.8 (9.5-14.4) | 650.9 (462.2-877.3) | 11.8 (9.6-14.4) | 0.9% (0.8-0.9) | 0.02 (0.02-0.02) |
| Suriname | 12.9 (9.2-17.3) | 14.2 (11.5-17.2) | 30.3 (21.5-40.8) | 14.2 (11.4-17.1) | 1.3% (1.3-1.4) | -0.02 (-0.02 to -0.01) |
| Sweden | 1132.1 (794.5-1533) | 20 (16.2-24.2) | 1546.3 (1069.6-2076.9) | 20.1 (16.3-24.3) | 0.4% (0.3-0.4) | -0.02 (-0.05-0.01) |
| Switzerland | 537.8 (376.7-725.4) | 13.8 (11.1-16.7) | 890.8 (623.4-1195.6) | 13.9 (11.2-16.8) | 0.7% (0.6-0.7) | -0.05 (-0.11-0) |
| Syrian Arab Republic | 185.4 (131.3-250.2) | 11.8 (9.6-14.4) | 453 (317.2-615.2) | 11.8 (9.5-14.3) | 1.4% (1.4-1.5) | -0.02 (-0.04 to -0.01) |
| Taiwan (Province of China) | 781.9 (555.5-1076) | 15.1 (12.2-18.1) | 2087.6 (1473.1-2803.7) | 14.5 (12.3-16.8) | 1.7% (1.5-1.9) | -0.15 (-0.19 to -0.12) |
| Tajikistan | 128.1 (90.6-171.4) | 14.9 (11.9-18) | 177 (124.7-242.9) | 14.9 (11.9-18) | 0.4% (0.2-0.5) | 0.01 (0.01-0.01) |
| Thailand | 1469.4 (1052.8-1976.9) | 14.3 (11.5-17.3) | 4953.3 (3540-6652) | 14.3 (11.5-17.3) | 2.4% (2.3-2.5) | 0.01 (0-0.01) |
| Timor-Leste | 9.7 (6.9-13.1) | 14.5 (11.6-17.5) | 38.6 (27.4-52.7) | 14.4 (11.6-17.5) | 3% (2.8-3.2) | -0.02 (-0.02 to -0.01) |
| Togo | 53.2 (38-71.6) | 14.5 (11.7-17.8) | 147.7 (104.8-199.4) | 14.4 (11.6-17.6) | 1.8% (1.7-1.8) | -0.03 (-0.03 to -0.03) |
| Tokelau | 0.1 (0.1-0.1) | 15.1 (12.2-18.3) | 0.1 (0-0.1) | 15.3 (12.3-18.5) | -0.1% (-0.2--0.1) | 0.04 (0.04-0.05) |
| Tonga | 2.6 (1.8-3.6) | 15.2 (12.2-18.3) | 3.9 (2.8-5.3) | 15.1 (12.2-18.3) | 0.5% (0.4-0.6) | -0.02 (-0.02 to -0.01) |
| Trinidad and Tobago | 45.4 (31.8-61.7) | 14.2 (11.5-17.2) | 102 (72.3-137.1) | 14.2 (11.5-17.2) | 1.2% (1.2-1.3) | 0.01 (0.01-0.01) |
| Tunisia | 194.6 (136.2-265.4) | 11.8 (9.5-14.4) | 503.2 (359-676) | 11.7 (9.5-14.3) | 1.6% (1.5-1.7) | -0.02 (-0.02 to -0.02) |
| Turkey | 1304 (924.6-1747.4) | 11.7 (9.5-14.3) | 3496.8 (2490.5-4710.2) | 11.7 (9.5-14.3) | 1.7% (1.6-1.7) | 0.03 (0.02-0.04) |
| Turkmenistan | 82 (58.7-111) | 14.8 (11.9-18) | 157.6 (112.7-210.6) | 14.9 (11.9-18.1) | 0.9% (0.9-1) | 0.03 (0.03-0.03) |
| Tuvalu | 0.3 (0.2-0.4) | 14.9 (12-18) | 0.5 (0.3-0.7) | 15.3 (12.3-18.4) | 0.6% (0.5-0.7) | 0.08 (0.07-0.08) |
| Uganda | 296.7 (211.1-401.2) | 14.6 (11.7-17.8) | 590.8 (423.7-793.5) | 14.5 (11.6-17.7) | 1% (1-1) | -0.03 (-0.03 to -0.02) |
| Ukraine | 4326.6 (3104.5-5877.1) | 17.8 (14.4-21.7) | 4824.7 (3450.5-6582.4) | 17.9 (14.4-21.7) | 0.1% (0.1-0.1) | 0.01 (0.01-0.01) |
| United Arab Emirates | 9.2 (6.4-12.7) | 12.3 (9.9-14.9) | 72.9 (48.8-102) | 12.4 (10-15.1) | 6.9% (6-7.6) | 0.07 (0.05-0.08) |
| United Kingdom | 6313.9 (4351-8636) | 18.6 (14.9-22.8) | 8645.2 (5947.6-11743.6) | 18.6 (15-22.7) | 0.4% (0.3-0.4) | -0.13 (-0.21 to -0.04) |
| United Republic of Tanzania | 503.7 (356.1-682.4) | 14.6 (11.7-17.8) | 1090.5 (779-1462.1) | 14.6 (11.7-17.8) | 1.2% (1.1-1.2) | 0 (0-0) |
| United States of America | 26038.7 (17914.1-35525.8) | 20 (16.1-24.4) | 38790.8 (29189.7-49361.5) | 18.1 (15.4-21.2) | 0.5% (0.3-0.7) | -0.93 (-1.12 to -0.74) |
| United States Virgin Islands | 4.1 (2.9-5.6) | 14.1 (11.4-17.1) | 11 (7.6-15) | 14.1 (11.4-17) | 1.7% (1.6-1.7) | -0.01 (-0.01-0) |
| Uruguay | 248.7 (175.1-334.3) | 15.3 (12.4-18.6) | 343.4 (241.8-460) | 15.3 (12.4-18.6) | 0.4% (0.3-0.4) | -0.01 (-0.01 to -0.01) |
| Uzbekistan | 509.6 (359.6-680.4) | 14.9 (11.9-18) | 729.3 (511-1010.6) | 14.9 (11.9-18) | 0.4% (0.3-0.6) | 0.01 (0-0.01) |
| Vanuatu | 2.9 (2-4) | 15.4 (12.4-18.6) | 8.1 (5.7-11) | 15.3 (12.3-18.5) | 1.8% (1.7-1.8) | -0.02 (-0.03 to -0.02) |
| Venezuela (Bolivarian Republic of) | 473.1 (336.5-637.3) | 14.2 (11.5-17.2) | 1501.4 (1067.4-2020.8) | 14.2 (11.5-17.1) | 2.2% (2.1-2.2) | 0 (0-0) |
| Viet Nam | 1837.4 (1307.8-2478) | 14.1 (11.4-17.1) | 3957.8 (2824.4-5283.9) | 14.2 (11.4-17.2) | 1.2% (1.1-1.2) | 0.02 (0.02-0.03) |
| Yemen | 164.8 (115.2-225) | 11.7 (9.4-14.2) | 455.6 (319.3-619.5) | 11.8 (9.5-14.3) | 1.8% (1.7-1.9) | 0.02 (0.02-0.02) |
| Zambia | 124.8 (89.6-167.4) | 14.7 (11.8-18) | 275.8 (197.6-371.1) | 14.6 (11.7-17.8) | 1.2% (1.2-1.2) | -0.03 (-0.03 to -0.03) |
| Zimbabwe | 185.4 (131.1-250.8) | 14.5 (11.7-17.7) | 302.4 (214.7-409.7) | 14.4 (11.6-17.6) | 0.6% (0.6-0.7) | -0.03 (-0.03 to -0.03) |

Supplementary Table2. The deaths of Cardiomyopathy and myocarditis between 1990 and 2019 at national level.

| location | Cases in 1990 | ASMR in 1990 | Cases in 2019 | ASMR in 2019 | Percentage change | EAPC (95%) |
| --- | --- | --- | --- | --- | --- | --- |
| Afghanistan | 138.1 (74.8-233.7) | 3.6 (1.9-6.5) | 159 (86-265.5) | 3 (1.6-5) | 0.2% (-0.2-0.6) | -0.89 (-1.07 to -0.71) |
| Albania | 154 (95.4-186.7) | 14.1 (9-16.8) | 220.9 (149.2-356.6) | 7.6 (5.2-12.3) | 0.4% (-0.1-1.8) | -2.27 (-2.57 to -1.96) |
| Algeria | 205 (146.8-302.1) | 3.7 (2.7-5.8) | 446.9 (310-675.5) | 2.6 (1.9-3.9) | 1.2% (0.5-2.1) | -1.32 (-1.35 to -1.29) |
| American Samoa | 0.8 (0.7-1.2) | 8.3 (6.7-11.5) | 1.7 (1.3-2.2) | 7.3 (5.7-9.7) | 1% (0.5-1.7) | -0.12 (-0.37-0.12) |
| Andorra | 2 (1.3-3.1) | 7.1 (4.4-10.1) | 5.2 (3.5-7.1) | 5.9 (4-7.7) | 1.6% (0.6-3) | -0.66 (-0.79 to -0.53) |
| Angola | 172.1 (90.4-258.4) | 10.1 (5.2-15.1) | 433.1 (254.2-631.5) | 8.8 (5.2-12.5) | 1.5% (0.7-2.7) | -0.56 (-0.61 to -0.51) |
| Antigua and Barbuda | 1.8 (1.5-2.1) | 5 (4.4-6) | 3.5 (2.6-4.3) | 6.1 (4.4-7.3) | 1% (0.3-1.6) | 0.9 (0.67-1.13) |
| Argentina | 3140.5 (2140.8-3767.5) | 15.2 (10.8-17.9) | 4243.7 (3348.9-4784.3) | 11.3 (8.9-12.6) | 0.4% (0.1-0.7) | -1.45 (-1.65 to -1.24) |
| Armenia | 89.1 (49.3-115.9) | 6.8 (4.4-8.5) | 101 (56.5-127.6) | 4 (2.7-4.9) | 0.1% (-0.1-0.5) | -2.35 (-2.97 to -1.72) |
| Australia | 853.9 (584.3-943.9) | 7.5 (5.3-8.3) | 909.9 (777.8-1131.9) | 3.5 (3.1-4.4) | 0.1% (-0.1-0.6) | -2.67 (-2.99 to -2.35) |
| Austria | 3266.3 (699.9-3933.5) | 36.3 (8.3-43.3) | 1067.2 (734.1-1240.8) | 8.1 (5.8-9.4) | -0.7% (-0.7-0.1) | -5.63 (-6.09 to -5.17) |
| Azerbaijan | 342.2 (264.3-459.6) | 16 (12.6-22.7) | 664.4 (486.4-943.6) | 17.3 (13.2-25.1) | 0.9% (0.4-1.6) | 0.16 (-0.15-0.47) |
| Bahamas | 6.8 (5.8-8.1) | 9.3 (7.9-11.1) | 17.3 (13.8-21.4) | 9.7 (7.7-12) | 1.6% (1-2.3) | 0.37 (0.27-0.47) |
| Bahrain | 4.5 (3.2-6.5) | 5.8 (4.5-8.3) | 17.1 (11.8-22.8) | 4.5 (3.2-5.9) | 2.8% (1.4-4.5) | -1.41 (-1.78 to -1.04) |
| Bangladesh | 172.7 (115.8-261.7) | 0.8 (0.5-1.1) | 510 (288.6-783.4) | 0.8 (0.4-1.2) | 2% (0.7-3.6) | 0.12 (-0.04-0.28) |
| Barbados | 12.5 (10.5-15.4) | 6.7 (5.8-7.9) | 22.3 (17.9-27) | 7.2 (5.8-8.7) | 0.8% (0.4-1.3) | 0.21 (0.15-0.26) |
| Belarus | 427 (335.8-535.4) | 9.7 (7.9-11.8) | 436.6 (300.7-783.7) | 10.4 (7.7-14.7) | 0% (-0.3-0.7) | -0.19 (-0.55-0.17) |
| Belgium | 865.7 (478.7-1023) | 7.6 (4.5-8.8) | 740 (553.5-865.2) | 4.3 (3.3-5) | -0.1% (-0.3-0.3) | -2.75 (-2.98 to -2.51) |
| Belize | 2.9 (2.1-3.8) | 5.7 (4.2-7.3) | 8.5 (6.6-10.1) | 6.5 (5.1-7.5) | 1.9% (1.1-3.3) | 0.56 (0.43-0.68) |
| Benin | 97.8 (53.6-138.2) | 8.9 (4.8-12.6) | 134.4 (95.6-177.8) | 5.8 (4.1-7.5) | 0.4% (-0.1-1.2) | -1.8 (-1.96 to -1.64) |
| Bermuda | 2.4 (1.9-3.3) | 6.4 (5.1-8.5) | 4.8 (3.3-6) | 5.4 (3.8-6.7) | 1% (0.2-1.8) | -0.41 (-0.57 to -0.26) |
| Bhutan | 0.7 (0.4-1.3) | 0.6 (0.4-1.1) | 2.3 (1.5-3.6) | 0.7 (0.5-1.1) | 2.2% (1.2-4) | 0.36 (0.3-0.42) |
| Bolivia (Plurinational State of) | 45.1 (28.5-63.4) | 3 (1.9-4.2) | 126.4 (83.9-174.7) | 2.7 (1.8-3.5) | 1.8% (1-3) | -0.48 (-0.51 to -0.45) |
| Bosnia and Herzegovina | 302 (180.6-425.7) | 14.4 (8.4-20.7) | 644.3 (354.7-924.2) | 15.8 (8.4-22.5) | 1.1% (0.5-2) | 0.31 (0.01-0.61) |
| Botswana | 51.4 (39.4-70.2) | 16.1 (12.3-21.7) | 83.9 (59.5-123) | 12.1 (8.6-18) | 0.6% (0.1-1.3) | -1.5 (-1.77 to -1.22) |
| Brazil | 7696.1 (5787.9-8282.8) | 15.9 (12.4-17.1) | 13201.2 (11566.2-15937.8) | 9.4 (8.3-11.1) | 0.7% (0.5-1.3) | -2.4 (-2.63 to -2.16) |
| Brunei Darussalam | 4.4 (3.3-6.2) | 10.8 (8.3-15.6) | 11.5 (8.6-13.9) | 9.6 (7.6-11.5) | 1.6% (0.8-2.7) | -0.18 (-0.28 to -0.07) |
| Bulgaria | 224.2 (140.2-663.2) | 3.5 (2.4-9.2) | 644.8 (486.1-983.8) | 6.7 (5.3-9.1) | 1.9% (0.3-3.8) | 1.89 (1.09-2.7) |
| Burkina Faso | 213.8 (108.9-334.1) | 9.7 (5-14.9) | 353.2 (248.3-492.1) | 7.7 (5.5-10.3) | 0.7% (0.1-1.6) | -0.76 (-0.82 to -0.71) |
| Burundi | 55.7 (37.1-80) | 5.6 (3.6-8.2) | 71 (48.8-96.2) | 3.9 (2.7-5.2) | 0.3% (-0.2-1) | -1.39 (-1.54 to -1.24) |
| Cabo Verde | 3.5 (2.6-4.6) | 2.5 (1.9-3.2) | 8.4 (6.2-11.1) | 3.3 (2.6-4.3) | 1.4% (0.7-2.1) | 0.05 (-0.29-0.38) |
| Cambodia | 71.3 (50-104) | 3.6 (2.4-5.3) | 214.8 (162.1-288.6) | 3.7 (2.8-4.9) | 2% (1.2-3.2) | 0.06 (0.02-0.09) |
| Cameroon | 190 (99.7-273.7) | 8.9 (4.7-12.4) | 329.4 (227-456.1) | 6.1 (4.3-8.5) | 0.7% (0.1-1.8) | -1.58 (-1.72 to -1.44) |
| Canada | 727.8 (631.5-888.7) | 3.6 (3.2-4.3) | 1044.8 (870.6-1460) | 2.5 (2.2-3.3) | 0.4% (0.2-1) | -1.78 (-2.02 to -1.53) |
| Central African Republic | 65.7 (33.9-105.9) | 12.1 (6-19.6) | 94.1 (50.1-154.7) | 10.3 (5.4-16.2) | 0.4% (0-1) | -0.61 (-0.65 to -0.57) |
| Chad | 133.7 (69-206.6) | 8.6 (4.5-13.2) | 171.9 (122.3-234.6) | 6.3 (4.5-8.5) | 0.3% (-0.1-1.1) | -1.25 (-1.37 to -1.14) |
| Chile | 318 (279.7-434.2) | 5.3 (4.6-7.1) | 569.6 (479.2-793.2) | 3.8 (3.3-4.9) | 0.8% (0.5-1.3) | -1.33 (-1.64 to -1.02) |
| China | 5744.5 (4401.1-9773.4) | 2 (1.5-3.4) | 16491 (10714.7-19888) | 1.8 (1.2-2.1) | 1.9% (0.5-3.2) | 0.09 (-0.22-0.4) |
| Colombia | 286.6 (246.1-315.8) | 3.1 (2.7-3.5) | 661.7 (492.4-853.1) | 2.1 (1.6-2.7) | 1.3% (0.8-2.1) | -2.28 (-2.82 to -1.75) |
| Comoros | 4.7 (2.9-6.7) | 4.6 (2.6-6.6) | 8 (5.2-11.1) | 3.5 (2.2-4.9) | 0.7% (0.2-1.7) | -1.09 (-1.29 to -0.89) |
| Congo | 61.8 (34.3-89.5) | 11.4 (6.1-15.9) | 103 (60.9-149.8) | 8.5 (5.2-12.2) | 0.7% (0.2-1.4) | -1.11 (-1.22 to -0.99) |
| Cook Islands | 0.1 (0.1-0.1) | 1.4 (1-1.9) | 0.1 (0.1-0.2) | 0.9 (0.6-1.1) | 0.4% (0-0.8) | -1.85 (-1.95 to -1.75) |
| Costa Rica | 54.4 (45.1-64.9) | 5.6 (4.7-6.4) | 122.8 (90.4-176.3) | 4.2 (3.1-5.9) | 1.3% (0.7-2.1) | -1.5 (-1.72 to -1.27) |
| Croatia | 333.1 (280.4-447.6) | 9.1 (7.9-11.2) | 484.9 (368.8-669.5) | 8.2 (6.5-10.5) | 0.5% (0.1-0.9) | -2.61 (-4.26 to -0.92) |
| Cuba | 152.3 (121.4-268.4) | 2.9 (2.4-4.6) | 583.9 (445.4-719.8) | 5.1 (4-6.3) | 2.8% (1-4.3) | 2.75 (2.47-3.02) |
| Cyprus | 34.4 (21.9-49.9) | 7.3 (4.5-10.9) | 53.9 (42.4-75.4) | 4.6 (3.6-6.3) | 0.6% (0.1-1.3) | -1.95 (-2.08 to -1.83) |
| Czechia | 308.8 (244.9-679.9) | 4 (3.5-7.2) | 486.9 (377.7-706.7) | 3.6 (2.9-4.7) | 0.6% (-0.1-1.1) | 0.91 (0.16-1.66) |
| C么te d'Ivoire | 144.1 (74.3-202.3) | 8.6 (4.5-11.9) | 247.1 (170.6-339.4) | 5.4 (3.8-7.3) | 0.7% (0.2-1.7) | -1.81 (-1.95 to -1.67) |
| Democratic People's Republic of Korea | 147.9 (103.7-230.2) | 2.6 (1.8-3.9) | 379.8 (262.7-538.1) | 2.5 (1.8-3.5) | 1.6% (0.8-2.7) | 0.02 (-0.15-0.18) |
| Democratic Republic of the Congo | 743.8 (414.8-1174.3) | 9.9 (5.5-15.2) | 1498.9 (847.2-2671.5) | 8.9 (5.2-15.3) | 1% (0.4-1.9) | -0.36 (-0.4 to -0.33) |
| Denmark | 133 (104.1-267.2) | 2.7 (2.3-4.7) | 166.5 (131-290.3) | 2.2 (1.9-3.5) | 0.3% (0-0.5) | -0.9 (-1.05 to -0.75) |
| Djibouti | 2.4 (1.4-3.5) | 4.9 (3-7) | 9.3 (5.3-14.1) | 4.2 (2.3-6.3) | 2.9% (1.6-4.8) | -0.63 (-0.73 to -0.54) |
| Dominica | 10.2 (8-13.9) | 20.3 (16.6-27.6) | 13.3 (9.7-16.9) | 22.7 (16.9-28.7) | 0.3% (-0.1-0.8) | 0.72 (0.52-0.93) |
| Dominican Republic | 35.5 (26.8-53.6) | 2.2 (1.8-3) | 133.9 (80.7-182.6) | 2.8 (1.7-3.8) | 2.8% (1.2-4.7) | 1.6 (1.32-1.87) |
| Ecuador | 46.9 (37-68.8) | 1.8 (1.4-2.6) | 142.7 (104.5-187.1) | 1.9 (1.4-2.4) | 2% (1.1-3.4) | 0.93 (0.41-1.45) |
| Egypt | 444.1 (302.7-640.8) | 4.8 (3.1-8.3) | 801.4 (511.9-1262.1) | 3.2 (2.2-4.9) | 0.8% (0.3-1.5) | -1.26 (-1.33 to -1.19) |
| El Salvador | 14.9 (10.8-18) | 1 (0.7-1.2) | 28.2 (19.4-38.2) | 0.8 (0.6-1.1) | 0.9% (0.4-1.7) | -0.97 (-1.11 to -0.83) |
| Equatorial Guinea | 10.5 (5.1-17.3) | 10.7 (5.1-17.3) | 17.2 (8.4-27.3) | 7.2 (3.6-11.4) | 0.6% (-0.1-2.1) | -1.65 (-1.86 to -1.45) |
| Eritrea | 15.2 (9.4-22.4) | 4.7 (2.9-6.8) | 37.9 (22.5-53.4) | 4 (2.4-5.7) | 1.5% (0.7-2.6) | -0.66 (-0.72 to -0.6) |
| Estonia | 85.2 (56-101.4) | 12.5 (7.9-15.1) | 112.5 (73.2-147.3) | 9.4 (6.7-12.2) | 0.3% (0-0.7) | -1.69 (-2.2 to -1.19) |
| Eswatini | 23.9 (17.7-31.9) | 15.2 (11.5-20.5) | 37.1 (25.8-56.5) | 12.2 (8.4-18.7) | 0.6% (0.1-1.2) | -0.69 (-0.91 to -0.47) |
| Ethiopia | 343.5 (208.6-513.3) | 4.6 (2.8-6.9) | 611.7 (404.5-828.9) | 3.2 (2.1-4.3) | 0.8% (0.1-2) | -1.35 (-1.43 to -1.26) |
| Fiji | 5.1 (3.9-6.9) | 3.7 (2.9-5) | 10.9 (7.5-14.3) | 3.6 (2.6-4.7) | 1.1% (0.5-2) | 0.2 (0-0.39) |
| Finland | 250 (181.6-504) | 7 (5.7-11) | 451 (381.1-644.5) | 6 (5.3-7.7) | 0.8% (0.2-1.4) | -0.18 (-0.37-0.02) |
| France | 4488.5 (3119.4-5093.8) | 7.4 (5.6-8.2) | 3844.6 (3067-4719.9) | 3.9 (3.2-4.7) | -0.1% (-0.3-0.2) | -2.7 (-2.89 to -2.51) |
| Gabon | 31.9 (16.9-47.2) | 10.3 (5.3-15) | 43.7 (24.2-61.6) | 8.1 (4.5-11.2) | 0.4% (-0.1-1) | -0.97 (-1.02 to -0.92) |
| Gambia | 13.5 (7.5-20) | 7.9 (4.4-11.7) | 29.6 (20.7-40.1) | 6 (4.3-8) | 1.2% (0.4-2.4) | -1.32 (-1.5 to -1.15) |
| Georgia | 123.2 (73.5-151.2) | 4 (2.8-4.7) | 157.9 (88.5-200) | 4.9 (3.1-6.1) | 0.3% (0-0.7) | 0.98 (0.77-1.19) |
| Germany | 8761.9 (7011.6-10214.2) | 10.1 (8.1-11.7) | 8322.7 (7280.9-9633) | 5.8 (5.1-6.8) | -0.1% (-0.2-0.2) | -2.22 (-2.4 to -2.05) |
| Ghana | 256.6 (194.8-361.3) | 9.1 (7-12.4) | 805.3 (512.5-1073.3) | 10.3 (6.8-13.4) | 2.1% (1-3.7) | 0.88 (0.66-1.1) |
| Greece | 671.5 (359.4-780.2) | 6.3 (3.6-7.2) | 643.2 (526.4-760.6) | 3.6 (2.8-4.1) | 0% (-0.2-0.6) | -2.24 (-2.46 to -2.02) |
| Greenland | 1.9 (1.4-2.4) | 13.9 (10.4-16.9) | 4.3 (2.9-5.4) | 11.2 (8-14.2) | 1.2% (0.5-2.1) | -0.89 (-1 to -0.79) |
| Grenada | 5.2 (4.5-6.1) | 10.9 (9.6-12.7) | 6.9 (5.2-8.1) | 11.2 (8.7-12.9) | 0.3% (0-0.6) | 0.41 (0.18-0.64) |
| Guam | 1.6 (1.2-2.1) | 4.7 (3.7-6.2) | 2.3 (1.6-3.6) | 2.8 (2.1-4.2) | 0.4% (0-1.3) | -1.9 (-2.08 to -1.72) |
| Guatemala | 20.2 (15.1-26.3) | 1.3 (1.1-1.6) | 73.4 (49.9-93.4) | 1.3 (0.9-1.7) | 2.6% (1.2-4.4) | 0.17 (-0.23-0.57) |
| Guinea | 160.6 (83.9-232.9) | 9 (4.6-12.8) | 182.2 (125.6-248) | 6.5 (4.5-8.6) | 0.1% (-0.3-0.8) | -1.22 (-1.3 to -1.14) |
| Guinea-Bissau | 19.1 (8.8-31.4) | 9.8 (4.4-15.8) | 20.5 (13.9-29.5) | 6.6 (4.5-9.2) | 0.1% (-0.3-0.9) | -1.51 (-1.61 to -1.4) |
| Guyana | 20.1 (16.5-25.5) | 11.2 (9.4-13.8) | 34.8 (25.4-44.2) | 12.2 (9-15.4) | 0.7% (0.2-1.4) | 0.74 (0.51-0.96) |
| Haiti | 191.4 (117.9-300.5) | 12.9 (7.7-20.1) | 323.7 (197.8-502.5) | 10.3 (6.1-15.6) | 0.7% (0.2-1.4) | -0.6 (-0.72 to -0.47) |
| Honduras | 59 (38.1-83.6) | 5.4 (3.6-7.5) | 216 (143.9-298.3) | 6.2 (4-8.5) | 2.7% (1.7-4) | 0.71 (0.52-0.9) |
| Hungary | 1907.5 (1422.8-2066.7) | 21.6 (16-23.4) | 1471.5 (1155.9-2147.6) | 11.8 (9.4-16.2) | -0.2% (-0.4-0.3) | -2.19 (-2.36 to -2.03) |
| Iceland | 5.3 (4-6.2) | 2.7 (2-3.1) | 5.9 (4.8-7.2) | 1.6 (1.3-1.8) | 0.1% (-0.1-0.4) | -2.28 (-2.43 to -2.14) |
| India | 1426.4 (987.9-2063.7) | 0.7 (0.5-1) | 3535.3 (2677.1-4635.5) | 0.6 (0.4-0.8) | 1.5% (0.9-2.2) | -0.85 (-0.91 to -0.79) |
| Indonesia | 1609 (1281.7-2251.1) | 3.8 (3-5.1) | 4551.7 (3194.8-6020.1) | 4.6 (3.1-6) | 1.8% (1.1-2.6) | 0.74 (0.68-0.8) |
| Iran (Islamic Republic of) | 297.3 (214-398.5) | 2.7 (2-3.5) | 751.1 (499.1-935.9) | 1.9 (1.3-2.4) | 1.5% (0.7-2.3) | -0.7 (-1.09 to -0.31) |
| Iraq | 220.7 (151.7-363.1) | 6.1 (4.4-9.3) | 571.2 (414.7-866.2) | 5.4 (4-7.9) | 1.6% (0.8-2.6) | -0.56 (-0.63 to -0.49) |
| Ireland | 351.5 (204.1-397.9) | 13.3 (7.4-15) | 221.6 (186-292.5) | 4.6 (3.9-5.7) | -0.4% (-0.5-0.1) | -3.85 (-4.37 to -3.32) |
| Israel | 102.5 (89.1-132.2) | 3.3 (2.9-4.2) | 121.8 (94.2-221) | 1.6 (1.3-2.8) | 0.2% (-0.1-0.8) | -3.31 (-3.64 to -2.99) |
| Italy | 15901.2 (4352.5-18512.9) | 26.6 (7.2-31.3) | 5071.4 (4292.8-5894.9) | 4.6 (3.8-5.4) | -0.7% (-0.7-0.2) | -6.81 (-7.22 to -6.4) |
| Jamaica | 41.8 (34.3-50.1) | 3.9 (3.2-4.6) | 62.5 (48.9-81.4) | 3.9 (3.1-5) | 0.5% (0.1-1) | -0.44 (-0.78 to -0.11) |
| Japan | 5107.8 (2458.4-5720.7) | 5.3 (2.9-5.9) | 5562.9 (3567.1-6377.4) | 2.3 (1.7-2.6) | 0.1% (-0.1-0.7) | -2.68 (-3.03 to -2.33) |
| Jordan | 7.9 (5.4-10.3) | 1.4 (0.9-1.8) | 19.8 (14.8-29.3) | 0.7 (0.5-1) | 1.5% (0.7-3.1) | -3.14 (-3.43 to -2.85) |
| Kazakhstan | 144 (81.6-450.8) | 3.4 (2-9.5) | 1722.8 (526.9-2209.2) | 26.2 (8.7-33.6) | 11% (0.4-23.6) | 10.31 (7.98-12.7) |
| Kenya | 123.9 (87.9-164.2) | 3.3 (2.4-4.4) | 323.8 (215-447.6) | 3.4 (2.3-4.7) | 1.6% (1.2-2.1) | 0.25 (0.16-0.34) |
| Kiribati | 0.5 (0.3-0.7) | 3.6 (1.9-4.8) | 0.7 (0.4-1) | 3.2 (1.5-4.5) | 0.4% (0-0.9) | -0.31 (-0.45 to -0.16) |
| Kuwait | 3.9 (3.2-4.8) | 1.8 (1.6-2.1) | 12.8 (10.1-16.3) | 1.2 (1-1.6) | 2.2% (1.4-3.3) | -1.41 (-1.83 to -0.99) |
| Kyrgyzstan | 43 (36.1-52.1) | 4.9 (3.7-5.7) | 100.4 (62.4-126.6) | 7.3 (4-9.1) | 1.3% (0.5-2.1) | 2.01 (1.56-2.47) |
| Lao People's Democratic Republic | 36.8 (24.2-57.1) | 3.9 (2.4-6.2) | 84.6 (62.4-112.9) | 4.3 (3.1-5.6) | 1.3% (0.6-2.4) | 0.31 (0.2-0.43) |
| Latvia | 155.1 (132.7-208.4) | 16.2 (13.5-18.6) | 331.3 (211.6-416.7) | 24.1 (17.2-30.7) | 1.1% (0.1-1.9) | 1.57 (1.04-2.12) |
| Lebanon | 42 (31.3-60.5) | 3.6 (2.7-5.2) | 72.7 (48.7-103.7) | 2.3 (1.6-3.3) | 0.7% (0.2-1.3) | -1.8 (-1.89 to -1.71) |
| Lesotho | 91.9 (67-128.9) | 15.3 (11.4-21.8) | 107.8 (75.7-166.5) | 15.4 (11-23.3) | 0.2% (-0.2-0.7) | 0.32 (0.19-0.44) |
| Liberia | 51.3 (27.9-73.5) | 8.6 (4.6-12.2) | 50.4 (34.5-73.1) | 5.5 (3.8-8.1) | 0% (-0.4-0.7) | -1.87 (-2.02 to -1.71) |
| Libya | 20 (13.5-30.9) | 3.8 (2.6-5.5) | 41.4 (27.6-62.3) | 2.2 (1.6-3.3) | 1.1% (0.4-2) | -1.84 (-1.93 to -1.75) |
| Lithuania | 116.3 (98.6-135.2) | 7.7 (6.6-8.9) | 193.9 (120.1-248.6) | 9.6 (6.6-12.2) | 0.7% (0.1-1.2) | 0.96 (0.22-1.71) |
| Luxembourg | 30.3 (22.3-34.4) | 8.2 (6.1-9.1) | 28.7 (22.8-36.2) | 4.1 (3.3-5.1) | -0.1% (-0.3-0.3) | -2.77 (-2.88 to -2.65) |
| Madagascar | 135 (93.1-176.7) | 7.2 (4.9-9) | 218.7 (144.6-310.3) | 5.9 (3.9-8.3) | 0.6% (0.1-1.2) | -0.79 (-0.84 to -0.73) |
| Malawi | 65.8 (46.2-85.5) | 4.4 (3-5.6) | 120.9 (81.5-162.6) | 3.9 (2.7-5.2) | 0.8% (0.3-1.6) | -0.39 (-0.44 to -0.35) |
| Malaysia | 164.3 (127.3-223.7) | 3.5 (2.8-4.7) | 418.1 (306.7-589.3) | 3 (2.3-4.2) | 1.5% (0.9-2.4) | -0.51 (-0.61 to -0.41) |
| Maldives | 0.7 (0.4-1.1) | 2.1 (1.3-3.4) | 2.4 (1.7-3.1) | 1.6 (1.2-2) | 2.4% (1-4.8) | -1.01 (-1.22 to -0.81) |
| Mali | 183.2 (96.5-283.8) | 9.2 (4.8-14.3) | 269.9 (189.2-369.4) | 6.6 (4.9-9) | 0.5% (0-1.5) | -1.41 (-1.55 to -1.28) |
| Malta | 33.9 (21.7-39.3) | 12.1 (8.2-13.9) | 25.9 (20.8-37.4) | 4.2 (3.4-6) | -0.2% (-0.4-0.5) | -3.81 (-4.08 to -3.54) |
| Marshall Islands | 0.4 (0.3-0.6) | 5.1 (3.2-7.3) | 0.6 (0.4-0.9) | 4.7 (2.7-6.8) | 0.5% (0.1-1.1) | -0.16 (-0.27 to -0.06) |
| Mauritania | 46.7 (24.4-63.6) | 8.8 (4.6-11.7) | 55.8 (38.5-74) | 5 (3.4-6.8) | 0.2% (-0.2-1.1) | -1.99 (-2.1 to -1.88) |
| Mauritius | 6.1 (4.9-10.8) | 2 (1.6-3.5) | 25.5 (16.4-32.7) | 3.4 (2.2-4.2) | 3.2% (0.8-5.2) | 2.24 (1.81-2.67) |
| Mexico | 263.1 (230.7-406.1) | 1.3 (1.1-1.9) | 747.2 (611-964.8) | 1.4 (1.2-1.6) | 1.8% (1.2-2.4) | 0.56 (0.47-0.65) |
| Micronesia (Federated States of) | 1.1 (0.7-1.5) | 4.9 (3-7) | 1.3 (0.7-2) | 4.6 (2.3-6.9) | 0.2% (-0.2-0.8) | -0.2 (-0.27 to -0.14) |
| Monaco | 6.9 (4.5-9) | 12.5 (8.2-16) | 6.4 (4.6-8.2) | 9.2 (6.7-11.5) | -0.1% (-0.4-0.3) | -1.03 (-1.12 to -0.94) |
| Mongolia | 50.4 (35.7-65.5) | 11.7 (8.9-15.1) | 79 (52.3-104.2) | 10.3 (7.4-13.6) | 0.6% (0.1-1.2) | -0.72 (-0.92 to -0.52) |
| Montenegro | 102.4 (55.7-153.2) | 26.6 (15.6-38.2) | 201.6 (114.9-280.6) | 30 (17-41) | 1% (0.4-2) | 0.52 (0.3-0.74) |
| Morocco | 225 (152.2-370) | 3.4 (2.3-5.4) | 475.6 (339.7-716.7) | 2.9 (2.1-4.4) | 1.1% (0.6-1.9) | -0.63 (-0.74 to -0.51) |
| Mozambique | 92 (68-128.7) | 3.7 (2.9-4.9) | 181.8 (131.5-258.6) | 4.1 (3-5.6) | 1% (0.4-1.7) | 0.47 (0.39-0.54) |
| Myanmar | 425.2 (280.8-657.2) | 4.2 (2.6-6.7) | 869.1 (668.3-1134.2) | 4 (3-5.2) | 1% (0.5-1.9) | -0.26 (-0.36 to -0.15) |
| Namibia | 69 (51.2-89.9) | 15.4 (11.5-20.1) | 105.1 (78.4-150) | 12.5 (9.4-17.8) | 0.5% (0.1-1.1) | -1.03 (-1.21 to -0.86) |
| Nauru | 0.1 (0-0.1) | 4.8 (2.8-7) | 0 (0-0.1) | 4.6 (2.6-6.9) | -0.2% (-0.4-0.2) | 0.01 (-0.29-0.32) |
| Nepal | 27.7 (18.2-43) | 0.6 (0.4-1) | 85.3 (55.4-125) | 0.7 (0.4-1) | 2.1% (1.2-3.4) | 0.26 (0.16-0.36) |
| Netherlands | 1343.7 (863.3-1524.7) | 10 (6.3-11.3) | 880.4 (707.5-1302.6) | 3.7 (3-5.3) | -0.3% (-0.5-0.3) | -3.74 (-4.02 to -3.46) |
| New Zealand | 140.8 (107.7-156.2) | 6.8 (5-7.5) | 167.9 (143.7-196.5) | 3.9 (3.2-4.3) | 0.2% (0-0.6) | -2.07 (-2.36 to -1.77) |
| Nicaragua | 16.7 (12.9-20.6) | 2.1 (1.7-2.5) | 61.1 (43.8-76.4) | 2.4 (1.8-3) | 2.7% (1.8-3.7) | 0.35 (0.14-0.56) |
| Niger | 114.5 (57.9-184.6) | 9 (4.5-14.9) | 222.2 (153.7-314.1) | 6.3 (4.4-8.9) | 0.9% (0.3-2.3) | -1.41 (-1.52 to -1.3) |
| Nigeria | 2068.6 (1162.6-3020.5) | 9.2 (5.1-13.4) | 2064 (1440.2-2676.9) | 5.1 (3.6-6.6) | 0% (-0.4-0.7) | -2.56 (-2.8 to -2.31) |
| Niue | 0 (0-0.1) | 3.7 (2.5-5) | 0 (0-0.1) | 3.3 (2-4.4) | -0.2% (-0.4-0.1) | -0.54 (-0.61 to -0.48) |
| North Macedonia | 250.6 (159.7-325.6) | 26 (16.2-34.1) | 414.9 (230.2-589.4) | 22.4 (11.9-32.4) | 0.7% (0.2-1.4) | -0.43 (-0.52 to -0.35) |
| Northern Mariana Islands | 0.3 (0.2-0.4) | 5.4 (4.1-8.1) | 1 (0.8-1.6) | 4.5 (3.3-6.9) | 2.5% (1.6-3.8) | -0.27 (-0.61-0.08) |
| Norway | 100.1 (75.5-237.4) | 2.5 (2-4.8) | 135 (111.5-210.1) | 2.2 (1.9-3.2) | 0.3% (-0.2-0.7) | -0.32 (-0.61 to -0.02) |
| Oman | 9.5 (6.5-14.9) | 3.3 (2.3-5.2) | 21 (11.1-29.1) | 3.3 (1.9-4.4) | 1.2% (0-2.9) | 1 (0.42-1.59) |
| Pakistan | 237.8 (162.2-366) | 0.7 (0.5-1.1) | 362.7 (263.8-491.4) | 0.7 (0.5-1) | 0.5% (0.2-1) | -0.27 (-0.4 to -0.15) |
| Palau | 0.1 (0-0.1) | 1.3 (1-1.8) | 0.1 (0.1-0.1) | 1.1 (0.8-1.4) | 0.5% (0.1-1.2) | -0.61 (-0.67 to -0.54) |
| Palestine | 29.5 (14.9-45.2) | 5.8 (3.1-8.7) | 39 (25.6-49.1) | 3.3 (2.3-4.1) | 0.3% (-0.1-1.1) | -2.16 (-2.28 to -2.05) |
| Panama | 25.2 (21.3-33.8) | 3.2 (2.8-4) | 103.3 (66.5-137.3) | 4.5 (2.8-6) | 3.1% (1.2-4.8) | 1.79 (1.43-2.16) |
| Papua New Guinea | 25.1 (14.4-39.8) | 3.3 (1.9-5.3) | 63.8 (39.3-91.9) | 3.7 (2.2-5.5) | 1.5% (0.9-2.7) | 0.5 (0.43-0.57) |
| Paraguay | 58.4 (34.4-75.1) | 4.5 (2.7-5.7) | 107.7 (78.3-148.9) | 3.2 (2.4-4.5) | 0.8% (0.2-2.2) | -1.47 (-1.65 to -1.29) |
| Peru | 117.5 (80.9-149.3) | 2.1 (1.4-2.6) | 196.9 (132.3-305.8) | 1.2 (0.8-1.7) | 0.7% (0-1.9) | -2.04 (-2.18 to -1.9) |
| Philippines | 550 (213.4-732.4) | 4.9 (1.6-6.7) | 1739.4 (735.6-2353.8) | 5.5 (2.1-7.5) | 2.2% (1.6-3.2) | 1.07 (0.72-1.43) |
| Poland | 5062.9 (3606.1-5462.6) | 16.4 (13.4-17.4) | 7380.3 (4830.5-8901.9) | 15.2 (10-18.3) | 0.5% (0.2-0.7) | -0.12 (-0.39-0.16) |
| Portugal | 379 (294.1-419) | 4.3 (3.5-4.7) | 479.5 (366-565.9) | 2.7 (2.1-3.1) | 0.3% (0.1-0.5) | -0.77 (-1.19 to -0.34) |
| Puerto Rico | 130.7 (71.5-155.2) | 5.6 (3.6-6.5) | 109.6 (81.6-147) | 2.3 (1.7-3.3) | -0.2% (-0.4-0.6) | -4.08 (-4.45 to -3.7) |
| Qatar | 1.5 (0.9-2.2) | 4.9 (2.9-7) | 6.7 (4.2-10) | 3.8 (2.4-5.3) | 3.5% (1.5-7) | -1.06 (-1.44 to -0.68) |
| Republic of Korea | 302.4 (190.2-384.2) | 2.4 (1.6-3.1) | 775.3 (471.3-966.2) | 1.4 (1-1.7) | 1.6% (0.7-2.7) | -1.68 (-1.95 to -1.41) |
| Republic of Moldova | 47.7 (38.3-78.6) | 3.3 (2.8-5) | 153.8 (114.7-183.6) | 6.3 (4.9-7.7) | 2.2% (0.7-3.4) | 2.84 (2.37-3.32) |
| Romania | 5026.6 (3204.3-6109.3) | 31.9 (19.9-38.5) | 6116.1 (4236.4-7468.9) | 22.9 (15.2-28.1) | 0.2% (-0.1-0.5) | -1.74 (-1.99 to -1.49) |
| Russian Federation | 8659.6 (7498.5-13780.6) | 13.7 (11.5-22.4) | 19916.8 (10960.5-24248.5) | 25.8 (16-31.7) | 1.3% (-0.1-2) | 2.31 (1.02-3.62) |
| Rwanda | 72.3 (48-99.7) | 6 (4-8.4) | 105.1 (70.9-146.3) | 4.1 (2.8-5.6) | 0.5% (0-1.2) | -1.66 (-1.9 to -1.42) |
| Saint Kitts and Nevis | 3.5 (3.1-4) | 14.5 (12.9-16.4) | 4.5 (3.5-5.2) | 12.4 (9.8-15.1) | 0.3% (0-0.5) | -0.24 (-0.53-0.04) |
| Saint Lucia | 6.1 (5-7.6) | 12.2 (10.5-15) | 13.3 (10.5-16.1) | 11.4 (9.1-13.6) | 1.2% (0.6-1.9) | -0.19 (-0.43-0.04) |
| Saint Vincent and the Grenadines | 1.6 (1.3-2.1) | 3.7 (3.3-4.9) | 3.6 (2.8-4.3) | 4.8 (3.8-5.7) | 1.3% (0.5-1.9) | 1.06 (0.83-1.3) |
| Samoa | 1.8 (1.2-2.3) | 4 (2.7-5.2) | 2.5 (1.6-3.4) | 3.6 (2.2-4.9) | 0.4% (0.1-0.9) | -0.24 (-0.31 to -0.17) |
| San Marino | 1.7 (1.1-2.3) | 7.6 (5-9.7) | 3.1 (1.9-4.4) | 6.2 (4-8.7) | 0.8% (0.1-1.8) | -0.81 (-0.9 to -0.73) |
| Sao Tome and Principe | 2.4 (1.3-3.4) | 6.8 (3.7-9.4) | 2.7 (2-3.6) | 5.5 (3.9-7.1) | 0.2% (-0.2-0.8) | -0.92 (-1.1 to -0.75) |
| Saudi Arabia | 251.6 (157.2-378.4) | 8.6 (5.7-12.7) | 375.9 (223.1-497.7) | 5.5 (3.5-6.8) | 0.5% (-0.1-1.3) | -1.66 (-1.73 to -1.59) |
| Senegal | 125.9 (67-185) | 7.6 (4.1-10.6) | 205.5 (141.7-273.4) | 5.5 (3.8-7.2) | 0.6% (0.1-1.7) | -1.23 (-1.33 to -1.14) |
| Serbia | 1448.2 (906.9-1910.5) | 20.9 (13.3-26.6) | 2162.9 (1231.8-3039.5) | 20.3 (11.1-27.7) | 0.5% (0.1-1.1) | -0.09 (-0.22-0.03) |
| Seychelles | 3.6 (2.8-4.5) | 10.6 (8.5-13.2) | 5 (3.9-6.3) | 8.8 (7.1-11.1) | 0.4% (0.1-0.7) | -0.94 (-1.12 to -0.76) |
| Sierra Leone | 84.9 (45.9-123.3) | 8.1 (4.4-11.6) | 98.2 (66.7-138.9) | 5.8 (4-8.3) | 0.2% (-0.2-0.9) | -1.28 (-1.37 to -1.18) |
| Singapore | 40.7 (35.6-57.2) | 5.3 (4.3-6) | 73.5 (58.6-114.6) | 2.1 (1.8-3) | 0.8% (0.5-1.6) | -3.48 (-3.61 to -3.35) |
| Slovakia | 173.5 (105.3-372.5) | 5.4 (3.9-9.6) | 305.6 (210.8-504.5) | 5.6 (4.2-8.2) | 0.8% (0.1-1.8) | 0.8 (0.54-1.06) |
| Slovenia | 1355.4 (273.1-1851.7) | 69.2 (16.5-93.7) | 505.3 (302.6-660.8) | 15 (8.8-19.3) | -0.6% (-0.7-0.3) | -5.11 (-5.46 to -4.75) |
| Solomon Islands | 2.4 (1.5-3.8) | 4.4 (2.5-6.9) | 4.9 (2.9-7.3) | 4.3 (2.4-6.7) | 1% (0.5-1.8) | 0.01 (-0.04-0.06) |
| Somalia | 47.3 (30.4-68.2) | 5.5 (3.5-7.8) | 107.3 (71-163.7) | 4.4 (2.9-6.5) | 1.3% (0.5-2.4) | -0.68 (-0.82 to -0.54) |
| South Africa | 1383.6 (998.1-1724.6) | 12 (9.1-14.6) | 2767.4 (2284.3-3534.6) | 10.7 (8.9-13.6) | 1% (0.7-1.4) | -0.57 (-0.93 to -0.21) |
| South Sudan | 48.3 (26-70.7) | 4.9 (2.6-7) | 49.1 (29.8-74.4) | 3.3 (2-4.9) | 0% (-0.3-0.5) | -1.33 (-1.5 to -1.16) |
| Spain | 3952.9 (2404-4543.5) | 10.8 (6.8-12.2) | 4033.7 (3155-4750) | 5.6 (4.5-6.3) | 0% (-0.1-0.5) | -2.52 (-2.66 to -2.38) |
| Sri Lanka | 888.4 (514-1115) | 16.3 (10.5-19.5) | 1307.7 (914.6-1871.2) | 8.6 (6-13) | 0.5% (-0.1-1.6) | -2.66 (-3.05 to -2.26) |
| Sudan | 177 (105.3-286.9) | 3.9 (2.2-6.7) | 250.1 (171-390.2) | 2.8 (1.9-4.5) | 0.4% (0-1) | -1.19 (-1.22 to -1.16) |
| Suriname | 7.1 (5.5-10.1) | 5.6 (4.6-7.9) | 17.1 (12.6-22) | 5.5 (4.2-7) | 1.4% (0.8-2.4) | -0.02 (-0.15-0.11) |
| Sweden | 465.7 (388.4-824.6) | 4.9 (4.4-7.4) | 474.7 (369.9-865) | 3.5 (2.9-5.7) | 0% (-0.1-0.3) | -1.06 (-1.14 to -0.98) |
| Switzerland | 365.7 (304.1-437.1) | 5 (4.2-5.8) | 345.2 (281-501.7) | 2.7 (2.3-3.9) | -0.1% (-0.3-0.3) | -2.73 (-3.01 to -2.45) |
| Syrian Arab Republic | 94 (65.5-144.3) | 4.1 (3-6.1) | 174.1 (120.6-273.9) | 2.9 (2.1-4.5) | 0.9% (0.2-1.7) | -1.42 (-1.59 to -1.26) |
| Taiwan (Province of China) | 259.1 (197.7-284.1) | 3 (2.4-3.2) | 356.4 (272.6-483.8) | 1.8 (1.4-2.3) | 0.4% (0-1.1) | -2.02 (-2.35 to -1.68) |
| Tajikistan | 12.2 (7.1-17.3) | 1 (0.7-1.2) | 21.2 (12.2-28.9) | 1.2 (0.7-1.5) | 0.7% (0.2-1.6) | 0.4 (0.29-0.52) |
| Thailand | 171.5 (127.4-245.6) | 1.2 (0.9-1.5) | 404.2 (265.3-669.7) | 0.8 (0.6-1.2) | 1.4% (0.6-2.5) | -1.82 (-2.05 to -1.58) |
| Timor-Leste | 3.4 (2.3-5.1) | 3.2 (2.2-4.6) | 18.1 (12.7-25.2) | 4.3 (3-5.9) | 4.3% (2.7-6.4) | 1.06 (1.01-1.12) |
| Togo | 49.8 (28-70) | 8.5 (4.7-11.8) | 87.7 (61.9-117.9) | 5.6 (4-7.6) | 0.8% (0.2-1.7) | -1.72 (-1.86 to -1.58) |
| Tokelau | 0 (0-0) | 3.8 (2.5-5.1) | 0 (0-0) | 3.2 (1.9-4.3) | -0.3% (-0.5-0) | -0.59 (-0.61 to -0.57) |
| Tonga | 0.6 (0.4-0.7) | 2 (1.5-2.5) | 0.8 (0.5-1) | 1.8 (1.2-2.4) | 0.4% (0-1) | -0.25 (-0.35 to -0.14) |
| Trinidad and Tobago | 26.4 (21.6-33.9) | 6.2 (5.2-7.3) | 46 (33.9-64.6) | 4.9 (3.6-6.5) | 0.7% (0.2-1.4) | -1.12 (-1.35 to -0.9) |
| Tunisia | 80.1 (56.7-119.9) | 3.1 (2.3-4.6) | 160.8 (108.8-251.2) | 2.2 (1.5-3.4) | 1% (0.4-1.9) | -1.21 (-1.24 to -1.19) |
| Turkey | 492.2 (329.4-844.7) | 2.9 (2.1-4.5) | 670 (428.7-1245.4) | 1.5 (1.1-2.4) | 0.4% (-0.1-0.9) | -2.67 (-2.83 to -2.5) |
| Turkmenistan | 155.1 (101.4-187.6) | 19.8 (14.5-23.3) | 217.8 (165.8-310.9) | 16.2 (12.5-23.2) | 0.4% (0-1.4) | -1.64 (-2.31 to -0.97) |
| Tuvalu | 0.2 (0.1-0.2) | 4.8 (2.9-6.6) | 0.2 (0.1-0.3) | 4 (2.4-5.5) | 0.2% (-0.1-0.8) | -0.61 (-0.66 to -0.56) |
| Uganda | 122.5 (83.7-159.5) | 4.3 (3-5.6) | 208.6 (133.6-274.9) | 3.7 (2.3-4.9) | 0.7% (0.2-1.3) | -0.72 (-0.8 to -0.64) |
| Ukraine | 3717.2 (3018.2-5346.1) | 17.3 (14.4-20.8) | 5095.5 (4160.4-6766.2) | 24.4 (19.7-31.4) | 0.4% (0.1-0.8) | 1.07 (0.78-1.36) |
| United Arab Emirates | 3.7 (1.7-6.8) | 3.2 (1.7-5.7) | 16.7 (7.6-34.3) | 2 (1-3.7) | 3.5% (1.9-5.6) | -1.9 (-2.15 to -1.64) |
| United Kingdom | 1755.4 (1399.8-3742.7) | 3.4 (2.9-5.8) | 2285.4 (1894.8-3670) | 3.3 (2.9-4.3) | 0.3% (-0.1-0.5) | -0.86 (-1.18 to -0.53) |
| United Republic of Tanzania | 261 (183.9-342.7) | 5.5 (3.8-6.8) | 466.9 (297.6-638.7) | 4.4 (2.8-6.1) | 0.8% (0.3-1.5) | -0.86 (-0.98 to -0.75) |
| United States of America | 17431.3 (13312-18681.3) | 8.5 (6.7-9) | 21372.4 (18923.9-24241) | 6.3 (5.6-7) | 0.2% (0.1-0.5) | -1.67 (-1.88 to -1.45) |
| United States Virgin Islands | 4.6 (3.6-6.2) | 10.7 (8.6-14.2) | 12.9 (9.7-15.9) | 10.9 (7.9-13.4) | 1.8% (1.1-2.8) | 0.56 (0.31-0.8) |
| Uruguay | 337.6 (223.7-387.7) | 12.1 (8.3-13.7) | 238 (199.7-330.5) | 5.9 (5-8.1) | -0.3% (-0.4-0.3) | -3.23 (-3.5 to -2.96) |
| Uzbekistan | 40 (21.3-73.3) | 0.9 (0.6-1.6) | 110.4 (83.1-181.1) | 1.9 (1.5-2.5) | 1.8% (0.7-4) | 3.29 (3.07-3.51) |
| Vanuatu | 1.1 (0.7-1.6) | 3.8 (2.4-5.5) | 3.1 (1.8-4.4) | 4.1 (2.3-5.9) | 1.7% (1-2.8) | 0.1 (0.02-0.18) |
| Venezuela (Bolivarian Republic of) | 303.5 (132.5-378.4) | 5.3 (2.6-6.4) | 528.3 (351.5-703) | 3 (2.1-3.9) | 0.7% (0.3-2.1) | -3.22 (-3.67 to -2.77) |
| Viet Nam | 1027.8 (734.5-1515.5) | 4.8 (3.5-6.9) | 2051.9 (1539.9-2747.4) | 4.5 (3.3-5.8) | 1% (0.4-1.9) | -0.23 (-0.33 to -0.14) |
| Yemen | 89.5 (52.9-167) | 3.8 (2.2-6.9) | 193.4 (126.2-322.1) | 3 (2-5) | 1.2% (0.4-2.2) | -0.92 (-0.97 to -0.87) |
| Zambia | 47.3 (29.6-65.7) | 4.2 (2.5-5.6) | 135 (82.4-187) | 4.9 (3-6.8) | 1.9% (1.1-3) | 0.61 (0.48-0.74) |
| Zimbabwe | 336.8 (253.2-416.2) | 15.4 (11.5-18.5) | 552 (406.5-714.5) | 15.8 (11.4-20.2) | 0.6% (0.2-1.2) | 0.34 (0.23-0.45) |

**Supplementary Table 3. The disability-adjusted life years (DALYs) of Cardiomyopathy and myocarditis between 1990 and 2019 at national level.**

| location | Cases in 1990 | ASDR in 1990 | Cases in 2019 | ASDR in 2019 | Percentage change | EAPC (95%) |
| --- | --- | --- | --- | --- | --- | --- |
| Afghanistan | 2710.2 (1454.5-4640) | 96.2 (48.5-200.2) | 2913.5 (1582.5-4912) | 73.5 (40.6-130.5) | 0.1% (-0.2-0.5) | -1.1 (-1.29 to -0.92) |
| Albania | 2452.5 (1597.7-2909.3) | 264.5 (191.5-303.9) | 3468.1 (2363.5-5603.2) | 158.8 (112.4-263) | 0.4% (-0.1-1.6) | -1.85 (-2.08 to -1.63) |
| Algeria | 3939.6 (2842.9-5767.1) | 104.7 (71.7-186) | 7973.2 (5372.2-12255.2) | 66 (46.8-100.7) | 1% (0.3-1.9) | -1.63 (-1.67 to -1.6) |
| American Samoa | 17.8 (13.8-24.1) | 240.7 (196.6-327.3) | 34.1 (26.9-43.9) | 219.5 (167.4-292.7) | 0.9% (0.5-1.6) | -0.02 (-0.23-0.2) |
| Andorra | 37.3 (24.5-54.8) | 143.8 (101.4-201.2) | 82.7 (57.9-110.2) | 110.4 (81.8-144.7) | 1.2% (0.4-2.3) | -0.98 (-1.09 to -0.88) |
| Angola | 3944 (2180.9-5687.4) | 295 (149.2-422.6) | 9832.5 (6279.7-13932) | 244.8 (157.3-331.9) | 1.5% (0.7-2.6) | -0.71 (-0.76 to -0.66) |
| Antigua and Barbuda | 31.3 (26.9-38.2) | 132 (117.8-159.4) | 65.5 (47.7-79.5) | 146.7 (109.8-175.9) | 1.1% (0.4-1.7) | 0.57 (0.38-0.76) |
| Argentina | 54477.1 (38801.9-64649) | 340.8 (274.9-387.8) | 69584.6 (57551.2-77834.4) | 226.6 (199.7-254.7) | 0.3% (0.1-0.6) | -1.83 (-2.01 to -1.65) |
| Armenia | 1589.6 (1083.8-1978.2) | 153.5 (125.8-184.7) | 1587.6 (1093.5-1957.9) | 81.9 (67-105.7) | 0% (-0.2-0.3) | -2.63 (-3.18 to -2.07) |
| Australia | 16539 (12406.6-18476.4) | 192.3 (154.8-206.8) | 17619.2 (15197.4-22075.1) | 96.1 (85.7-122.8) | 0.1% (-0.1-0.5) | -2.46 (-2.67 to -2.25) |
| Austria | 42726.7 (11707-50674.3) | 471.5 (174.6-544.7) | 15445.3 (11961-17595) | 122 (107.4-154.1) | -0.6% (-0.7-0.1) | -5.04 (-5.44 to -4.64) |
| Azerbaijan | 6597.9 (5062.5-9282) | 470.2 (352.7-712.2) | 12958 (9280-19731.6) | 449.3 (324.3-751.7) | 1% (0.5-1.5) | -0.44 (-0.72 to -0.15) |
| Bahamas | 132.1 (111.9-158.4) | 272.8 (233.4-327.4) | 338.9 (268.8-421) | 280.1 (219.8-349.4) | 1.6% (1-2.3) | 0.29 (0.19-0.4) |
| Bahrain | 94.9 (68.6-136.2) | 153 (124.1-212.4) | 372.1 (256.9-493.8) | 101.2 (74.6-136.9) | 2.9% (1.5-4.7) | -2 (-2.3 to -1.69) |
| Bangladesh | 3239.7 (2194.7-4841.6) | 20.5 (13.4-30.4) | 9111 (5188.4-13705.9) | 19.6 (11.3-29.5) | 1.8% (0.6-3.5) | 0 (-0.12-0.11) |
| Barbados | 221 (188.1-268.8) | 184.1 (161.6-214.7) | 414.7 (334.7-504.9) | 183.3 (145.3-225.5) | 0.9% (0.5-1.4) | -0.04 (-0.11-0.03) |
| Belarus | 8910.5 (6783.3-11575.4) | 341.4 (275.2-411.3) | 9688.3 (6577.1-17466.9) | 401.6 (296.6-541) | 0.1% (-0.3-0.7) | 0.26 (-0.09-0.6) |
| Belgium | 13233.7 (8194.9-15246.3) | 135.3 (99.8-149.5) | 11011.8 (8915.3-12716.7) | 74.5 (65.7-91.4) | -0.2% (-0.3-0.2) | -2.64 (-2.84 to -2.45) |
| Belize | 54.4 (39.5-70.9) | 167.3 (127.5-206.8) | 165.4 (129-196.7) | 179 (142.4-208.8) | 2% (1.2-3.4) | 0.25 (0.15-0.35) |
| Benin | 2070.1 (1245-2848.2) | 237.1 (136.8-319.7) | 3084.3 (2285.4-4048.3) | 163.6 (118.5-219.6) | 0.5% (0-1.2) | -1.56 (-1.71 to -1.41) |
| Bermuda | 44.6 (34.7-60) | 155.6 (124.6-212.2) | 80.8 (57.8-101) | 124.1 (91.7-152.6) | 0.8% (0.2-1.6) | -0.63 (-0.81 to -0.45) |
| Bhutan | 14.3 (8.6-24.8) | 16.3 (9.1-27.9) | 40.9 (26.5-62) | 16.8 (11.2-25.8) | 1.9% (1-3.4) | 0.06 (-0.03-0.15) |
| Bolivia (Plurinational State of) | 832.4 (528.7-1175.6) | 92.6 (52-138.4) | 2260.8 (1503.3-3167.7) | 68.3 (44.7-91.7) | 1.7% (0.9-3) | -1.08 (-1.11 to -1.05) |
| Bosnia and Herzegovina | 5046.4 (3315.7-6870.7) | 259.8 (177.5-349.2) | 9836.9 (5669.9-13909) | 272.6 (154.4-388.1) | 0.9% (0.4-1.7) | 0.18 (-0.03-0.4) |
| Botswana | 1078.6 (824.5-1447.6) | 361.1 (281.2-492.1) | 1775.1 (1257.1-2535.7) | 284.8 (203.3-420.9) | 0.6% (0.1-1.3) | -1.29 (-1.55 to -1.04) |
| Brazil | 145294.4 (110654.9-156527.3) | 399.4 (319.6-426.4) | 240524.7 (215071.1-288377) | 238.6 (213-272.3) | 0.7% (0.5-1.2) | -2.24 (-2.45 to -2.02) |
| Brunei Darussalam | 86.7 (66.4-123.7) | 315.2 (248.3-447.5) | 225.4 (168-271.7) | 248.4 (202.3-299.1) | 1.6% (0.8-2.6) | -0.69 (-0.85 to -0.53) |
| Bulgaria | 4037.8 (2626.7-11228.4) | 91.2 (70.8-187.9) | 9859.3 (7565-14563.6) | 152.3 (121.3-191.4) | 1.4% (0.2-3) | 1.36 (0.61-2.12) |
| Burkina Faso | 4715.3 (2617-7040.1) | 246.7 (132.2-364.6) | 7758.3 (5694.1-10485.3) | 202.1 (146.1-270.3) | 0.6% (0.1-1.4) | -0.75 (-0.86 to -0.64) |
| Burundi | 1464.3 (1039.6-2008) | 217.3 (134.3-313.4) | 2063.4 (1511.3-2737.1) | 148.4 (106.7-202.8) | 0.4% (0-1) | -1.34 (-1.49 to -1.2) |
| Cabo Verde | 103.5 (77.8-134.8) | 91.9 (72.3-113.6) | 215.2 (164.8-279) | 101.3 (81.2-127.7) | 1.1% (0.7-1.5) | -0.15 (-0.32-0.03) |
| Cambodia | 1372.5 (972.9-2086.9) | 96.7 (60.9-178.9) | 3868.3 (2913.6-5500.3) | 86.1 (65.3-126.9) | 1.8% (1.1-2.9) | -0.52 (-0.58 to -0.45) |
| Cameroon | 4061.8 (2315.1-5673.1) | 223.7 (122.2-304.9) | 7560.9 (5351.9-10344.2) | 169.5 (119.6-235) | 0.9% (0.2-1.9) | -1.18 (-1.29 to -1.07) |
| Canada | 18471.7 (15739.3-22222.8) | 124.5 (110.9-141.2) | 22120.2 (18702-29453.6) | 79 (70.4-95.6) | 0.2% (0-0.6) | -1.83 (-2.01 to -1.65) |
| Central African Republic | 1504.6 (803-2306.5) | 339.6 (162.9-518.1) | 2128.8 (1203.1-3392.2) | 285.7 (147-423.2) | 0.4% (0-1) | -0.62 (-0.66 to -0.58) |
| Chad | 2818.6 (1602.8-4145.7) | 221.8 (121.2-317.6) | 3908.9 (2849.3-5260.1) | 172.3 (124.4-232.6) | 0.4% (-0.1-1.2) | -1.01 (-1.11 to -0.91) |
| Chile | 6482.1 (5702.8-8323.9) | 140.3 (124.8-174.8) | 10834.7 (9337.5-14306.1) | 97.8 (87.6-116.9) | 0.7% (0.4-1.2) | -1.34 (-1.57 to -1.12) |
| China | 114691.6 (90137.9-185390.3) | 72.9 (57.1-125.1) | 284691.6 (199351.9-340756.5) | 49.3 (35.3-57.9) | 1.5% (0.4-2.4) | -1.14 (-1.38 to -0.89) |
| Colombia | 5493.8 (4713.5-6002.1) | 87.3 (77.3-98) | 11598.3 (8871.6-15017.3) | 57.8 (43.3-77.3) | 1.1% (0.6-1.8) | -2.03 (-2.42 to -1.64) |
| Comoros | 127.7 (86.2-174.3) | 183.3 (101.5-261.3) | 233 (164.7-311.5) | 134.4 (88.1-179.7) | 0.8% (0.3-1.5) | -1.17 (-1.36 to -0.97) |
| Congo | 1387.1 (808.8-1955.5) | 314 (168-425.9) | 2300.4 (1483.3-3249.7) | 232.1 (148-323.4) | 0.7% (0.2-1.3) | -1.15 (-1.27 to -1.03) |
| Cook Islands | 1.7 (1.2-2.3) | 45.3 (32.3-62.2) | 2.2 (1.7-2.9) | 25.5 (18.5-33.3) | 0.3% (-0.1-0.7) | -2.01 (-2.11 to -1.92) |
| Costa Rica | 995.6 (831.1-1169) | 148.9 (129.4-166.2) | 2274 (1707-3193.2) | 110.8 (84.2-147.3) | 1.3% (0.7-2.1) | -1.42 (-1.61 to -1.22) |
| Croatia | 4863.8 (4114.9-7080.8) | 151.9 (133-215.3) | 7034.3 (5352.3-9865.2) | 134.3 (104.3-184.2) | 0.4% (0.1-0.8) | -2.19 (-3.5 to -0.86) |
| Cuba | 3033.5 (2451.8-5021.7) | 96 (82-134.8) | 10868.9 (8398.2-13387.1) | 140.2 (109.5-171.4) | 2.6% (1.1-3.9) | 1.86 (1.67-2.05) |
| Cyprus | 598.8 (398.1-815.9) | 153.7 (101.5-193.8) | 872.7 (701.4-1138.4) | 89.6 (68.6-108.1) | 0.5% (0.1-1.1) | -2.22 (-2.35 to -2.1) |
| Czechia | 5733.6 (4737.5-10975.2) | 125.2 (105.1-151.7) | 9166.7 (7270-11600.6) | 98 (69.6-122.6) | 0.6% (-0.1-1.1) | 0.23 (-0.41-0.88) |
| C么te d'Ivoire | 3340.8 (1860.2-4599.9) | 223.6 (123-303.8) | 5986.3 (4248.8-8037.4) | 151.9 (107.9-199.3) | 0.8% (0.2-1.7) | -1.55 (-1.69 to -1.41) |
| Democratic People's Republic of Korea | 2869.5 (2039.1-4409.5) | 83.1 (52.7-142.6) | 6661.5 (4825.2-9288.1) | 69.4 (51.8-95.8) | 1.3% (0.7-2.3) | -0.43 (-0.54 to -0.31) |
| Democratic Republic of the Congo | 17082.3 (9997.1-25994.6) | 280.6 (155.7-400.2) | 32980.2 (20227-55638.7) | 240.3 (148.8-386) | 0.9% (0.3-1.8) | -0.51 (-0.54 to -0.48) |
| Denmark | 2491.9 (2030.3-4433) | 79.1 (69.6-103.5) | 2921.3 (2396.4-4499.3) | 55.5 (48.9-66.1) | 0.2% (0-0.4) | -1.5 (-1.68 to -1.31) |
| Djibouti | 66.6 (44.6-92.8) | 189.9 (119-276.5) | 276.9 (178-386.8) | 159.2 (97.4-244.1) | 3.2% (2-4.6) | -0.64 (-0.72 to -0.56) |
| Dominica | 177.9 (140.3-241.8) | 513.8 (417.5-689.5) | 234.3 (169.1-301) | 560.6 (410.3-718) | 0.3% (-0.1-0.8) | 0.57 (0.42-0.72) |
| Dominican Republic | 666.2 (504.8-996.2) | 69.4 (56.6-95.2) | 2416.8 (1501.1-3286.4) | 78.6 (51.4-105.9) | 2.6% (1.2-4.4) | 0.99 (0.7-1.29) |
| Ecuador | 826.5 (663-1188.7) | 48.5 (39.6-71.4) | 2501.9 (1863.3-3244.2) | 48.6 (36.7-63.5) | 2% (1.1-3.2) | 0.52 (0.03-1.01) |
| Egypt | 8658.2 (5938.2-12616.1) | 227 (124.5-464) | 16129.2 (10167.3-25569.6) | 122.2 (83.5-184.6) | 0.9% (0.3-1.6) | -2.07 (-2.15 to -1.99) |
| El Salvador | 283.2 (207.7-340.2) | 30.6 (22-38.8) | 510.5 (351.9-685.3) | 22.3 (15.7-30) | 0.8% (0.3-1.5) | -1.23 (-1.38 to -1.07) |
| Equatorial Guinea | 235.1 (122-366.2) | 304.2 (139.5-468.6) | 375.7 (205.6-576.6) | 190.2 (101-301.8) | 0.6% (-0.1-1.9) | -1.88 (-2.08 to -1.68) |
| Eritrea | 415.2 (273.4-594) | 177.6 (104.3-255.9) | 1047.2 (707.2-1419.1) | 145.9 (94.8-203.9) | 1.5% (0.8-2.5) | -0.74 (-0.79 to -0.69) |
| Estonia | 1988.6 (1245.7-2404.7) | 448.2 (281.5-546) | 2380.7 (1512.6-3139.2) | 303.6 (216.8-395.8) | 0.2% (-0.1-0.6) | -2.23 (-2.77 to -1.7) |
| Eswatini | 491.6 (367.7-653) | 345.1 (264.1-453.4) | 778.3 (551.7-1152.2) | 282 (197.2-417.7) | 0.6% (0.1-1.2) | -0.6 (-0.77 to -0.42) |
| Ethiopia | 9868.2 (6616.1-13971.9) | 184.3 (110.4-267.4) | 18267.7 (13412.2-23330.8) | 123.4 (89.6-155.9) | 0.9% (0.3-1.7) | -1.45 (-1.52 to -1.39) |
| Fiji | 105.7 (81-141.9) | 115.2 (91.1-154.1) | 224 (156.5-297.4) | 120.6 (85.8-157.4) | 1.1% (0.5-2.1) | 0.38 (0.22-0.54) |
| Finland | 5127.5 (3920.9-8683.3) | 221.1 (172.5-265.7) | 8450.3 (7363-10650.8) | 165.8 (134.3-184.2) | 0.6% (0.1-1.2) | -0.71 (-0.91 to -0.51) |
| France | 72286.6 (55073.1-80767.9) | 152.1 (128.2-164) | 59481.6 (49706.4-72575.6) | 77 (66.2-98.2) | -0.2% (-0.3-0.1) | -2.83 (-3.01 to -2.64) |
| Gabon | 702.6 (393.3-1007.1) | 289 (150.6-411.1) | 968.7 (568.9-1339.3) | 220.9 (128.3-306.2) | 0.4% (-0.1-0.9) | -1.02 (-1.07 to -0.96) |
| Gambia | 306.8 (185.6-432.2) | 206 (120.5-294) | 679.8 (497.9-903.4) | 164.5 (121.8-217.4) | 1.2% (0.5-2.3) | -1.09 (-1.27 to -0.91) |
| Georgia | 2307.4 (1500-2831.6) | 109.9 (89.2-129.1) | 3035.8 (1646-3926.7) | 143.2 (94.5-179.2) | 0.3% (-0.1-0.8) | 1.18 (1.02-1.33) |
| Germany | 146476.6 (118652.7-169077.3) | 224.7 (183.8-255.5) | 132201.7 (116174.9-151890) | 121.1 (109-139.1) | -0.1% (-0.3-0.1) | -2.44 (-2.59 to -2.28) |
| Ghana | 5463.9 (4197.6-7448.1) | 222.9 (173.7-297.6) | 16878.8 (11201.3-22450.3) | 253.7 (175.6-331.7) | 2.1% (1-3.6) | 0.86 (0.66-1.07) |
| Greece | 9924.9 (5856.8-11249.3) | 105.9 (71.1-116.9) | 9502.8 (7598.3-10864.4) | 74.9 (53-84.2) | 0% (-0.2-0.4) | -1.31 (-1.62 to -1.01) |
| Greenland | 41.5 (30.8-52.8) | 438.5 (340.4-530.1) | 89.4 (61.4-113.2) | 301.3 (218.1-387.5) | 1.2% (0.5-1.9) | -1.45 (-1.55 to -1.35) |
| Grenada | 91.3 (78.6-106.3) | 295.3 (260.8-346.1) | 135 (100.8-158.4) | 282.3 (213.7-326.1) | 0.5% (0.1-0.8) | 0.2 (-0.05-0.46) |
| Guam | 32.9 (25.2-44.4) | 135.3 (110.6-170.7) | 46.3 (33.2-70.6) | 101.2 (76.7-145.8) | 0.4% (0-1.2) | -0.97 (-1.1 to -0.84) |
| Guatemala | 394.5 (295.8-520.3) | 37.5 (28.2-52.1) | 1307.2 (901-1667.8) | 36.1 (25.9-46.6) | 2.3% (1-3.9) | 0.06 (-0.38-0.5) |
| Guinea | 3456.4 (1981.7-4908) | 237.9 (129.2-328.2) | 4161.5 (3021.7-5487.6) | 183.1 (130.3-244.6) | 0.2% (-0.2-0.8) | -0.94 (-1.01 to -0.86) |
| Guinea-Bissau | 427.8 (212.8-680.9) | 264.1 (120.5-431.3) | 487.9 (337.6-695.9) | 186.5 (129.1-260.8) | 0.1% (-0.3-0.9) | -1.27 (-1.35 to -1.19) |
| Guyana | 399.7 (324.2-512) | 334.3 (280.7-402.8) | 703.5 (510.8-903.4) | 352.6 (255.2-457.7) | 0.8% (0.2-1.5) | 0.7 (0.47-0.93) |
| Haiti | 3895 (2423.1-6066.2) | 393.4 (215.6-628.4) | 6453.3 (3964.8-10034.7) | 315.9 (178.9-514.2) | 0.7% (0.1-1.4) | -0.53 (-0.69 to -0.38) |
| Honduras | 1109.6 (737.7-1566.4) | 136.9 (96-178.2) | 4045.2 (2685.9-5621) | 137.9 (86.7-192.3) | 2.6% (1.7-4) | 0.15 (0.03-0.27) |
| Hungary | 30679.3 (23719.8-32974) | 450.7 (343.4-482.3) | 26906.9 (21487.9-36420.3) | 300.5 (241.9-383.1) | -0.1% (-0.3-0.3) | -1.61 (-1.74 to -1.49) |
| Iceland | 88.4 (67.6-101) | 59.7 (46.9-66.5) | 98.5 (82.8-115.1) | 32.9 (28.1-38.8) | 0.1% (-0.1-0.4) | -2.4 (-2.56 to -2.23) |
| India | 28905.9 (20329.6-41199.1) | 18.6 (12.8-26.7) | 66765.7 (50936.9-86933.2) | 15.3 (11.8-19.8) | 1.3% (0.8-2) | -0.75 (-0.8 to -0.7) |
| Indonesia | 31040.2 (24727.7-45259.9) | 94 (72.1-147.2) | 83302.6 (60046.6-116546.9) | 93.9 (68.5-130) | 1.7% (1.1-2.3) | 0.08 (0.04-0.13) |
| Iran (Islamic Republic of) | 6159.7 (4632.1-8231.4) | 77.8 (59.5-104.7) | 13525.1 (9366.5-16911.8) | 50.5 (37.2-62.7) | 1.2% (0.6-1.8) | -1.04 (-1.36 to -0.71) |
| Iraq | 4259.9 (3002.5-6708.9) | 199 (146.1-281.4) | 11067.5 (8129.1-16431.8) | 146.3 (111.8-199.5) | 1.6% (0.8-2.6) | -1.1 (-1.21 to -0.98) |
| Ireland | 5300.1 (3438.7-5920.9) | 222 (151.1-243.2) | 3629.3 (3109.9-4721.1) | 86.8 (75-109) | -0.3% (-0.4-0) | -3.61 (-3.97 to -3.25) |
| Israel | 1862.3 (1613-2172.5) | 88.7 (71.5-97.4) | 2121.3 (1735.2-3380.7) | 38.4 (33.1-53.8) | 0.1% (-0.1-0.7) | -3.7 (-4.02 to -3.39) |
| Italy | 216721.8 (72487.8-249458) | 351 (149.6-398.3) | 80742.9 (69958-95236.1) | 80.1 (69.4-107.4) | -0.6% (-0.7-0.1) | -5.73 (-6.09 to -5.37) |
| Jamaica | 780.4 (630.9-928.2) | 116.9 (94.5-135.7) | 1199.4 (940.7-1524) | 114.4 (87.5-145.1) | 0.5% (0.2-1.1) | -0.44 (-0.79 to -0.09) |
| Japan | 82724.1 (47421.4-92393.6) | 124.7 (96.7-133.4) | 90774 (65628-103478.6) | 64.7 (57.3-75.7) | 0.1% (0-0.6) | -2.38 (-2.52 to -2.24) |
| Jordan | 155.8 (109.3-204.9) | 38 (23.2-50.2) | 395.1 (299.7-577.6) | 18.8 (15.1-25.2) | 1.5% (0.7-3.1) | -3.23 (-3.57 to -2.89) |
| Kazakhstan | 3257.7 (1886.9-9703.1) | 119.4 (71.7-323) | 35530.3 (11132.5-46084.2) | 828.1 (306.1-1070.2) | 9.9% (0.4-21) | 9.59 (7.31-11.91) |
| Kenya | 3381.9 (2538.8-4454.4) | 123.2 (90.2-155) | 8740.7 (6254.3-11736.4) | 115.8 (78.6-157.9) | 1.6% (1.3-2) | -0.11 (-0.19 to -0.04) |
| Kiribati | 11.5 (6-15.6) | 128.1 (62.2-181.8) | 15.9 (8.1-22.2) | 114.1 (50.8-173.5) | 0.4% (0-0.9) | -0.26 (-0.4 to -0.12) |
| Kuwait | 82.8 (66.3-101.6) | 59.5 (51.1-68.2) | 252.7 (202.2-317.6) | 35.6 (28.8-44.3) | 2.1% (1.3-3.1) | -1.93 (-2.3 to -1.56) |
| Kyrgyzstan | 1043.9 (847-1261.4) | 185.1 (134.3-214.8) | 2466.3 (1420.6-3135.2) | 272.1 (137.7-338.9) | 1.4% (0.5-2.2) | 1.93 (1.48-2.38) |
| Lao People's Democratic Republic | 715.7 (477.3-1147.8) | 92.9 (55.5-198.9) | 1534.7 (1140.6-2151) | 98.6 (70.2-145.9) | 1.1% (0.5-2.2) | 0.25 (0.12-0.39) |
| Latvia | 3652.2 (3136.2-4608.4) | 623.6 (488.7-708.1) | 7093.1 (4575.1-8898.3) | 861.7 (628-1082.3) | 0.9% (0.1-1.6) | 1.13 (0.59-1.68) |
| Lebanon | 798.8 (598.7-1153) | 102.1 (75.1-156.7) | 1280.3 (889.7-1819) | 63.1 (43.4-96) | 0.6% (0.1-1.2) | -1.82 (-1.91 to -1.74) |
| Lesotho | 1860.4 (1364.7-2565.6) | 338.7 (258.6-468.4) | 2215.7 (1556.1-3334.8) | 340.6 (244.7-510.6) | 0.2% (-0.2-0.7) | 0.34 (0.22-0.45) |
| Liberia | 1129.3 (688.1-1571.5) | 228.5 (129.7-313.5) | 1175.6 (846.2-1658) | 150.5 (106.2-215.1) | 0% (-0.3-0.6) | -1.68 (-1.85 to -1.51) |
| Libya | 380.5 (264.4-577.5) | 205.1 (123.6-334.4) | 777.8 (535.4-1150) | 98.1 (67.8-147.1) | 1% (0.4-1.9) | -2.59 (-2.66 to -2.52) |
| Lithuania | 2680.5 (2192.1-3139.9) | 282.3 (242-329.8) | 4289 (2525.9-5543) | 337.2 (236.3-428.7) | 0.6% (0.1-1.1) | 0.75 (0.01-1.5) |
| Luxembourg | 496.4 (383.9-556.4) | 164.7 (131-179.5) | 462.3 (377.5-577.7) | 77.1 (64.2-99.5) | -0.1% (-0.2-0.3) | -2.99 (-3.11 to -2.87) |
| Madagascar | 3426.6 (2525.1-4374.1) | 293.3 (200.5-372.1) | 5942 (4054.3-7975) | 227.3 (156-333) | 0.7% (0.3-1.3) | -0.89 (-0.93 to -0.84) |
| Malawi | 1884.5 (1380.7-2452.3) | 180.7 (120.4-242.4) | 3500.2 (2537.4-4578.8) | 147.2 (103.2-193.4) | 0.9% (0.4-1.5) | -0.67 (-0.73 to -0.62) |
| Malaysia | 3477.4 (2669.1-4600.6) | 108.3 (86-134.7) | 8998 (6639.3-12254.4) | 89.9 (66.2-115.2) | 1.6% (0.9-2.4) | -0.5 (-0.59 to -0.4) |
| Maldives | 15.2 (10-24) | 71.1 (42-121.8) | 44 (31.9-55.8) | 42.5 (30.8-54.9) | 1.9% (0.7-3.8) | -1.84 (-2.07 to -1.61) |
| Mali | 3959 (2256.6-5827.8) | 233.7 (122.2-337.1) | 5868.9 (4429.9-7869.5) | 172.4 (127.6-233.5) | 0.5% (0-1.4) | -1.31 (-1.46 to -1.16) |
| Malta | 527.9 (367.4-599) | 218.9 (167.5-241.1) | 454.2 (374.5-605.3) | 93.3 (78-120.8) | -0.1% (-0.3-0.5) | -3.02 (-3.24 to -2.81) |
| Marshall Islands | 8.2 (5.2-11.8) | 143.3 (88.3-213.6) | 13 (8-19) | 140.7 (80.2-209.8) | 0.6% (0.1-1.2) | 0.03 (-0.1-0.15) |
| Mauritania | 1024.9 (586.6-1376) | 230.3 (129.2-302.4) | 1307.6 (916.4-1714.1) | 135.8 (91.2-187.5) | 0.3% (-0.1-1.1) | -1.81 (-1.92 to -1.71) |
| Mauritius | 130.9 (104.4-224) | 63.1 (52.4-107.6) | 501.1 (325-644.7) | 102.2 (67.2-130) | 2.8% (0.7-4.7) | 2.06 (1.66-2.46) |
| Mexico | 5016 (4439.7-7434.5) | 40.2 (36.9-53.4) | 14015.6 (11714.4-17959.1) | 45.8 (35.7-53.4) | 1.8% (1.2-2.3) | 0.87 (0.7-1.05) |
| Micronesia (Federated States of) | 22.4 (13.9-30.9) | 143.9 (82.4-218.1) | 27.9 (15.2-43.2) | 134.7 (61.5-210.6) | 0.2% (-0.2-0.8) | -0.2 (-0.24 to -0.16) |
| Monaco | 106.4 (71.1-136.1) | 256.2 (177.1-322.9) | 95.8 (70.4-119.6) | 171.9 (127.3-217.8) | -0.1% (-0.4-0.2) | -1.35 (-1.47 to -1.23) |
| Mongolia | 971.9 (719-1261) | 328 (244.2-470.3) | 1561.9 (1100.7-2099.5) | 287.1 (212.9-419.9) | 0.6% (0.1-1.3) | -0.81 (-1 to -0.62) |
| Montenegro | 1640.5 (961.8-2380.7) | 509.5 (339.4-684.8) | 3127.1 (1895.2-4286.9) | 517.4 (320.4-690.5) | 0.9% (0.4-1.8) | 0.1 (-0.13-0.32) |
| Morocco | 4258.5 (2895.9-6861.8) | 97 (62.5-167) | 8831.9 (6277.7-13366.2) | 72.2 (50.6-113.9) | 1.1% (0.5-1.8) | -1.05 (-1.11 to -1) |
| Mozambique | 2653.7 (1962.9-3547.9) | 148.9 (112.1-192.3) | 5365.5 (3920.6-7101.4) | 154.1 (116.9-200.3) | 1% (0.6-1.5) | 0.31 (0.21-0.4) |
| Myanmar | 8297.8 (5507-13231.4) | 115.9 (67.5-255.3) | 15491.5 (12184.1-21137) | 100.2 (72-153.4) | 0.9% (0.3-1.7) | -0.55 (-0.67 to -0.43) |
| Namibia | 1420.9 (1064.3-1830.3) | 341.9 (253.2-443.2) | 2035.6 (1529.7-2863.8) | 272.8 (201.9-392.8) | 0.4% (0-1) | -1.01 (-1.15 to -0.87) |
| Nauru | 1.2 (0.7-1.7) | 149.8 (84.5-236.2) | 1.1 (0.6-1.6) | 149.1 (84.4-236) | -0.1% (-0.3-0.3) | 0.08 (-0.24-0.41) |
| Nepal | 548.2 (363.1-846.6) | 15.9 (10.1-23.9) | 1573.8 (1033.7-2294.8) | 15.6 (10.4-22.8) | 1.9% (1-3.1) | -0.07 (-0.16-0.02) |
| Netherlands | 20118.6 (14363.4-22313.3) | 178.2 (133.5-193.4) | 13872.9 (11370-20764.7) | 67.6 (57-100.3) | -0.3% (-0.4-0.2) | -3.79 (-4.04 to -3.54) |
| New Zealand | 2895.1 (2138-3239.3) | 214.2 (148.4-236.7) | 3351.8 (2821.3-3819) | 118.2 (92.5-129.6) | 0.2% (0-0.5) | -2.21 (-2.49 to -1.94) |
| Nicaragua | 319.2 (247.8-385.5) | 61.3 (46.4-76.1) | 1102.8 (787.2-1386.1) | 55.9 (41.1-69.4) | 2.5% (1.7-3.4) | -0.34 (-0.43 to -0.25) |
| Niger | 2587 (1434.4-3904.6) | 236.6 (122.9-363.4) | 5183.9 (3664.1-7198) | 168.5 (118.2-234.6) | 1% (0.4-2.2) | -1.38 (-1.52 to -1.24) |
| Nigeria | 44624.5 (26690.4-63969) | 235.6 (134.6-343.8) | 47866.8 (35167.6-61473.6) | 137.1 (100.7-177.9) | 0.1% (-0.3-0.7) | -2.23 (-2.45 to -2.02) |
| Niue | 0.8 (0.6-1.1) | 117.5 (79.5-156.4) | 0.7 (0.5-0.9) | 104.8 (64.7-146.1) | -0.1% (-0.3-0.1) | -0.5 (-0.6 to -0.41) |
| North Macedonia | 3810 (2568.9-4809.1) | 414.4 (295.7-504.2) | 6366.1 (3632.7-8666.7) | 356 (205.1-479.5) | 0.7% (0.2-1.3) | -0.51 (-0.57 to -0.45) |
| Northern Mariana Islands | 6.6 (5-9.6) | 161 (122.3-237) | 23.4 (17.4-34.5) | 134.4 (99.9-204.2) | 2.5% (1.6-3.9) | -0.2 (-0.52-0.13) |
| Norway | 1867.2 (1467-3834.2) | 77.2 (69.8-108.2) | 2399.4 (2064-3377.4) | 55 (48.7-63.3) | 0.3% (-0.2-0.5) | -1.08 (-1.28 to -0.87) |
| Oman | 196.3 (136.5-313.6) | 100.9 (68.4-156.9) | 423.4 (234.5-593.7) | 94.4 (51.9-127.8) | 1.2% (0-2.9) | 0.74 (0.17-1.32) |
| Pakistan | 4339.9 (2991.3-6534.5) | 19.1 (12.3-27.6) | 6941.7 (5060.5-9475.5) | 19.9 (14.1-27.7) | 0.6% (0.2-1.1) | -0.03 (-0.15-0.09) |
| Palau | 1.3 (1-1.7) | 45.1 (30.8-64.4) | 2.1 (1.5-2.7) | 37.4 (26.2-48.5) | 0.6% (0.1-1.3) | -0.55 (-0.61 to -0.49) |
| Palestine | 543.9 (285.8-825.5) | 145.6 (82.4-210.7) | 745.2 (513.5-936.5) | 79.9 (60.2-97.4) | 0.4% (-0.1-1.2) | -2.2 (-2.3 to -2.11) |
| Panama | 483.2 (413-629) | 91.1 (82.1-112.4) | 1857.3 (1224-2449.5) | 121.8 (76-164.1) | 2.8% (1.2-4.4) | 1.6 (1.24-1.96) |
| Papua New Guinea | 540 (314.2-849) | 107.2 (56.1-181.1) | 1317.9 (825.3-1926.1) | 118.7 (65.6-191.4) | 1.4% (0.7-2.5) | 0.51 (0.41-0.61) |
| Paraguay | 1047.7 (649-1332) | 101.8 (68.7-123.9) | 1966.7 (1446.2-2713.1) | 73.5 (54.7-105) | 0.9% (0.3-2.2) | -1.42 (-1.57 to -1.27) |
| Peru | 2109 (1473.2-2654) | 67 (42.4-85.9) | 3620.4 (2591.1-5361.6) | 34.4 (24.6-49) | 0.7% (0.1-1.8) | -2.44 (-2.69 to -2.2) |
| Philippines | 8527.5 (4257-10810.8) | 69.9 (38.8-85.1) | 28501.6 (15165.8-36613.9) | 103.2 (60-129.9) | 2.3% (1.7-3.4) | 2.09 (1.61-2.57) |
| Poland | 83346.6 (61541.8-90040.5) | 332.9 (289.9-358) | 116151.7 (81240.2-138677.7) | 283.7 (207.2-338.1) | 0.4% (0.2-0.6) | -0.33 (-0.46 to -0.19) |
| Portugal | 6116.5 (5027.6-6709.4) | 91.5 (78.3-99) | 7414.4 (5564.6-8565.3) | 56.6 (41.8-64) | 0.2% (0-0.4) | -0.99 (-1.35 to -0.62) |
| Puerto Rico | 2349.4 (1376.5-2770.4) | 145.5 (103.4-163.5) | 1979.7 (1521.6-2663.4) | 63.2 (47-95.1) | -0.2% (-0.4-0.6) | -3.88 (-4.24 to -3.53) |
| Qatar | 31.8 (20.7-45.8) | 133.1 (91.6-175.9) | 161 (106.5-237.8) | 74.7 (52.3-100.2) | 4.1% (1.9-7.9) | -2.29 (-2.48 to -2.09) |
| Republic of Korea | 5754.4 (3888-7127.8) | 71.7 (53.2-104.9) | 13524.3 (8988.3-16231.8) | 38.3 (29.1-45) | 1.4% (0.6-2.3) | -2.12 (-2.33 to -1.91) |
| Republic of Moldova | 1109.5 (894-1800.8) | 123.8 (108-179.6) | 3502.5 (2627.6-4223.4) | 218.5 (172.7-266) | 2.2% (0.7-3.3) | 2.45 (2.01-2.88) |
| Romania | 78053.5 (53939.9-92026.3) | 555.8 (411-638.2) | 96178.7 (65149.5-118070.4) | 468.3 (291.8-583.8) | 0.2% (0-0.5) | -0.96 (-1.15 to -0.78) |
| Russian Federation | 181769.7 (152232.1-317202.3) | 461.7 (376.8-803.5) | 407533.9 (251541.5-495299.6) | 924 (607.2-1133.6) | 1.2% (-0.1-2.1) | 2.37 (0.96-3.79) |
| Rwanda | 1851.7 (1319.8-2471.1) | 220.1 (141.1-304.1) | 2837 (2002.2-3811) | 146.5 (101.5-199.4) | 0.5% (0.1-1.2) | -1.64 (-1.82 to -1.47) |
| Saint Kitts and Nevis | 64.7 (56.7-73.4) | 388 (339.9-441.4) | 90.1 (70.6-107.3) | 301.9 (228.3-384.4) | 0.4% (0.1-0.7) | -0.73 (-1.03 to -0.43) |
| Saint Lucia | 112.4 (92.2-140.4) | 308.9 (265.9-380) | 243.5 (193.2-294.8) | 290 (231.4-349.7) | 1.2% (0.6-1.8) | -0.08 (-0.28-0.13) |
| Saint Vincent and the Grenadines | 28.3 (24.4-38) | 105.5 (92.8-136.7) | 65.2 (52.3-78.8) | 128 (102.6-153.4) | 1.3% (0.6-1.9) | 0.75 (0.58-0.91) |
| Samoa | 34.8 (24.5-45.3) | 117.2 (79.4-159.4) | 47.1 (30.7-62.4) | 105.1 (63.8-145) | 0.4% (0-0.9) | -0.22 (-0.27 to -0.17) |
| San Marino | 28.8 (19.8-36.3) | 147.7 (107.7-181.3) | 46.3 (30.4-65) | 114 (76-163.8) | 0.6% (0-1.5) | -1.02 (-1.14 to -0.9) |
| Sao Tome and Principe | 53.7 (32.7-73.9) | 183.4 (110.1-247.3) | 65.6 (48.7-84.4) | 155.2 (113.7-200.9) | 0.2% (-0.1-0.8) | -0.83 (-1 to -0.66) |
| Saudi Arabia | 4900.5 (3104.3-7292.9) | 217.1 (155-310.2) | 8117.9 (5019.4-10850) | 132.9 (90.2-168) | 0.7% (0-1.5) | -1.85 (-1.92 to -1.77) |
| Senegal | 2882.9 (1726.7-3997.4) | 204.8 (120.1-275.2) | 4954.4 (3590.4-6405.6) | 154.9 (110.2-201.5) | 0.7% (0.2-1.6) | -1 (-1.12 to -0.88) |
| Serbia | 21529.6 (14461.3-27422.3) | 359.4 (256.2-434.2) | 31058.6 (18233.3-42221.7) | 305.7 (178.5-417.5) | 0.4% (0.1-0.9) | -0.63 (-0.75 to -0.51) |
| Seychelles | 67.4 (52-84.1) | 291.2 (237.4-360.9) | 96.2 (74.1-119.5) | 241.5 (196-303.4) | 0.4% (0.1-0.8) | -0.94 (-1.12 to -0.76) |
| Sierra Leone | 1848.7 (1116.7-2579.5) | 220.1 (124.1-308.5) | 2340.6 (1676.9-3188.4) | 170.6 (120-239.6) | 0.3% (-0.1-0.9) | -0.97 (-1.07 to -0.87) |
| Singapore | 839 (735.4-1110.5) | 212.9 (163.6-230.8) | 1478.2 (1203.3-2156.2) | 72.6 (62.8-92.5) | 0.8% (0.5-1.6) | -3.95 (-4.13 to -3.76) |
| Slovakia | 3109.7 (2071.8-6146.5) | 152.3 (129-214.7) | 5725.4 (4239.8-8347.5) | 150 (103.1-197.2) | 0.8% (0.1-1.9) | 0.61 (0.36-0.86) |
| Slovenia | 19055.3 (4396.1-26301.1) | 1017.1 (327.9-1391.5) | 7012.9 (4639-8982.5) | 208.9 (151.3-263.8) | -0.6% (-0.7-0.2) | -5.43 (-5.78 to -5.08) |
| Solomon Islands | 54 (32.1-85) | 138.2 (70.1-232.5) | 101.8 (60.5-153.8) | 132.4 (69.5-220.5) | 0.9% (0.3-1.6) | -0.06 (-0.12 to -0.01) |
| Somalia | 1232.7 (846-1696.6) | 209.3 (127.1-298.7) | 2993.7 (2122.2-4270.1) | 165.5 (115.6-228) | 1.4% (0.7-2.4) | -0.65 (-0.81 to -0.49) |
| South Africa | 27465.6 (20869.4-33635) | 296.2 (238.4-341.5) | 54891.2 (45556.6-69716.5) | 235.8 (196.7-295.5) | 1% (0.7-1.4) | -0.99 (-1.26 to -0.72) |
| South Sudan | 1296.5 (814.7-1802.9) | 199.3 (108.4-284) | 1444 (980.6-2044.8) | 134.9 (88.6-199) | 0.1% (-0.2-0.5) | -1.28 (-1.48 to -1.08) |
| Spain | 59176.8 (41226.2-66108.7) | 184.5 (149.6-201.5) | 60515 (47858.3-69368.4) | 106.6 (85.9-120.9) | 0% (-0.1-0.3) | -2.03 (-2.15 to -1.9) |
| Sri Lanka | 17058.1 (9991.5-21367.2) | 407.8 (278.7-479.5) | 26671.1 (18900.1-37202.7) | 235.4 (168.3-338.1) | 0.6% (0-1.6) | -2.37 (-2.77 to -1.97) |
| Sudan | 3429.1 (2068.1-5591.5) | 127.9 (64.9-276.2) | 4663.9 (3163.9-7273.4) | 86.5 (56.4-143.7) | 0.4% (-0.1-1) | -1.31 (-1.35 to -1.26) |
| Suriname | 134.4 (105.3-186.3) | 170.3 (137.9-223.5) | 324.9 (242.3-416.4) | 158.3 (118.4-200.6) | 1.4% (0.8-2.3) | -0.27 (-0.37 to -0.16) |
| Sweden | 8712.7 (7574.8-13146.5) | 149.4 (129.1-169.8) | 8988 (7335.5-13826.9) | 93.9 (84.5-116.2) | 0% (-0.1-0.3) | -1.5 (-1.58 to -1.42) |
| Switzerland | 6246.4 (5228.1-7373.3) | 115.5 (95-131.9) | 5516 (4673.2-7495.4) | 55.5 (48.3-72.1) | -0.1% (-0.3-0.2) | -3.17 (-3.43 to -2.9) |
| Syrian Arab Republic | 1798.9 (1275.1-2723.7) | 133.9 (94.5-205.8) | 3351.8 (2344.4-5325.9) | 83 (59.2-127.8) | 0.9% (0.2-1.7) | -1.97 (-2.25 to -1.7) |
| Taiwan (Province of China) | 5153 (4041.7-5665.7) | 76.7 (66.5-82.8) | 7024.9 (5503-9274.6) | 55.8 (42.4-70.6) | 0.4% (0.1-0.9) | -1.17 (-1.39 to -0.95) |
| Tajikistan | 244.5 (148.3-338.9) | 32.6 (24-39.8) | 450.8 (263.7-621.3) | 34.7 (23.6-44.6) | 0.8% (0.3-1.6) | 0.01 (-0.12-0.14) |
| Thailand | 3573.3 (2692.2-4932.1) | 36.3 (26.4-48.7) | 8692.3 (6007.2-13223.8) | 26.6 (20-35.7) | 1.4% (0.7-2.4) | -1.58 (-1.86 to -1.29) |
| Timor-Leste | 65.2 (45.2-100.8) | 72.6 (46-128.6) | 330.4 (234.8-474.6) | 92.1 (64.4-133.7) | 4.1% (2.6-5.9) | 0.86 (0.77-0.96) |
| Togo | 1114.5 (681.9-1542.4) | 225.4 (132.6-299.6) | 2151 (1591.1-2820.6) | 160.7 (117.3-213.5) | 0.9% (0.3-1.8) | -1.41 (-1.54 to -1.28) |
| Tokelau | 0.6 (0.4-0.8) | 112.5 (71.7-155) | 0.4 (0.3-0.5) | 93.9 (56.6-131.8) | -0.3% (-0.5-0) | -0.58 (-0.65 to -0.5) |
| Tonga | 11.6 (8.4-15.2) | 59.3 (46.3-75.3) | 15.5 (10.8-20.4) | 56.7 (39.6-77.6) | 0.3% (0-0.9) | -0.14 (-0.26 to -0.02) |
| Trinidad and Tobago | 483.8 (397.2-634.7) | 175.8 (152.5-201.6) | 881.1 (646.9-1245.5) | 145.1 (105.7-192.3) | 0.8% (0.3-1.5) | -1 (-1.23 to -0.77) |
| Tunisia | 1531.9 (1069.6-2310.5) | 93.2 (70.1-136.6) | 2956.8 (2027.3-4626.8) | 57.5 (39.1-91.7) | 0.9% (0.4-1.8) | -1.65 (-1.68 to -1.62) |
| Turkey | 9841.7 (6688.2-16438) | 101.1 (71.4-140.4) | 12720.8 (8347.7-22994.5) | 47.3 (37.6-62.6) | 0.3% (-0.1-0.8) | -2.77 (-2.88 to -2.66) |
| Turkmenistan | 3373.9 (2186.6-4106.6) | 696.1 (505.9-808.2) | 4889 (3722-6852.2) | 602.1 (469.9-802.9) | 0.4% (0-1.5) | -1.4 (-2.01 to -0.78) |
| Tuvalu | 3.3 (2.1-4.6) | 150.5 (85.2-228.2) | 3.8 (2.4-5.3) | 121.1 (73.2-169.7) | 0.1% (-0.2-0.6) | -0.7 (-0.75-0.65) |
| Uganda | 3073.2 (2201.4-3971.7) | 156.4 (107.8-203.4) | 5320.5 (3670.4-6886) | 133.1 (84.2-183.4) | 0.7% (0.3-1.2) | -0.69 (-0.79 to -0.59) |
| Ukraine | 74887.9 (58200.4-119194.5) | 563.8 (471-696.1) | 94086.2 (73512.5-144854.6) | 864.5 (674.1-1164.5) | 0.3% (0-0.6) | 1.33 (0.95-1.71) |
| United Arab Emirates | 80.1 (37.9-144.4) | 95 (55.3-157.6) | 405.7 (190.2-830.4) | 57.5 (33.4-99.4) | 4.1% (2.3-6.6) | -1.92 (-2.06 to -1.78) |
| United Kingdom | 31678.5 (26235.9-60669.9) | 102 (95.3-128.2) | 36694.6 (31787.7-55822.2) | 80.6 (70.5-89.6) | 0.2% (-0.2-0.3) | -1.39 (-1.62 to -1.16) |
| United Republic of Tanzania | 6857.6 (5076.2-8876.7) | 208.8 (147.8-263.8) | 12880.5 (8980.2-17198.4) | 169.8 (112.1-251.1) | 0.9% (0.4-1.5) | -0.68 (-0.78 to -0.58) |
| United States of America | 337775.4 (255388.4-367956.9) | 249.5 (197-265.9) | 407712 (370234-470164.9) | 176.4 (162.8-197.4) | 0.2% (0.1-0.6) | -1.67 (-1.83 to -1.51) |
| United States Virgin Islands | 89.6 (69.1-121.8) | 292.3 (235.8-387.9) | 247.7 (183.9-307.9) | 272.6 (196.3-346.2) | 1.8% (1-2.8) | 0.07 (-0.08-0.23) |
| Uruguay | 6223.3 (4256.9-7111.4) | 289.3 (206.9-324.1) | 4187.2 (3567.9-5727.5) | 139.2 (122.1-186.2) | -0.3% (-0.5-0.2) | -3.22 (-3.46 to -2.99) |
| Uzbekistan | 719.2 (394.4-1459.5) | 29.3 (20.9-53) | 2392.4 (1758.5-4108.2) | 62 (50.4-80.1) | 2.3% (1.1-4.8) | 3.35 (3.05-3.65) |
| Vanuatu | 23.2 (14.4-32.2) | 112.6 (67.7-168.3) | 61.7 (37.8-87.5) | 128 (71.5-186.7) | 1.7% (1-2.7) | 0.3 (0.2-0.39) |
| Venezuela (Bolivarian Republic of) | 5434.3 (2572.5-6698.2) | 119.7 (70.7-140.1) | 9392.6 (6504.3-12369.7) | 66 (49.4-85.6) | 0.7% (0.3-2) | -3.26 (-3.7 to -2.81) |
| Viet Nam | 18475 (13372.3-28715.6) | 111.6 (82.2-177.7) | 35726.4 (27169.4-49460.6) | 98.4 (72.8-146.1) | 0.9% (0.4-1.6) | -0.38 (-0.45 to -0.31) |
| Yemen | 1807.2 (1087.4-3285.9) | 109.2 (59-218.6) | 3729.7 (2444.4-6271.1) | 83 (54.8-141.8) | 1.1% (0.4-2) | -0.98 (-1.04 to -0.92) |
| Zambia | 1294.5 (898.3-1716.1) | 167.9 (101.7-227) | 3548 (2421.8-4795) | 169.2 (110.3-233.4) | 1.7% (1.1-2.6) | 0.12 (0.01-0.22) |
| Zimbabwe | 7021.1 (5361.9-8814.6) | 349 (257.2-418.3) | 11388 (8543.7-14556.4) | 374.5 (269.7-480) | 0.6% (0.2-1.1) | 0.54 (0.42-0.67) |
